# Smad-Independent BMP Signaling in Somatic Cells Limits the Size of the Germline Stem Cell Pool

**DOI:** 10.1016/j.stemcr.2018.07.008

**Published:** 2018-08-16

**Authors:** Chen-Yuan Tseng, Yu-Han Su, Shun-Min Yang, Kun-Yang Lin, Chun-Ming Lai, Elham Rastegari, Oyundari Amartuvshin, Yueh Cho, Yu Cai, Hwei-Jan Hsu

**Affiliations:** 1 Institute of Cellular and Organismic Biology, Academia Sinica, Taipei 11529, Taiwan; 2 Molecular and Biological Agricultural Sciences Program, Taiwan International Graduate Program, Academia Sinica and National Chung-Hsing University, Taipei 11529, Taiwan; 3 Graduate Institute of Biotechnology and Biotechnology Center, National Chung-Hsing University, Taichung 40227, Taiwan; 4 Temasek Life Science Laboratory, National University of Singapore, Singapore 117604, Singapore; 5 Department of Biological Sciences, National University of Singapore, Singapore 117543, Singapore

**Keywords:** PGC, GSC, niche, Egfr, Hh, BMP, soma-germline interaction, escort cells

## Abstract

In developing organisms, proper tuning of the number of stem cells within a niche is critical for the maintenance of adult tissues; however, the involved mechanisms remain largely unclear. Here, we demonstrate that Thickveins (Tkv), a type I bone morphogenetic protein (BMP) receptor, acts in the *Drosophila* developing ovarian soma through a Smad-independent pathway to shape the distribution of BMP signal within the niche, impacting germline stem cell (GSC) recruitment and maintenance. Somatic Tkv promotes Egfr signaling to silence transcription of Dally, which localizes BMP signals on the cell surface. In parallel, Tkv promotes Hh signaling, which promotes escort cell cellular protrusions and upregulates expression of the *Drosophila* BMP homolog, Dpp, forming a positive feedback loop that enhances Tkv signaling and strengthens the niche boundary. Our results reveal a role for non-canonical BMP signaling in the soma during GSC establishment and generally illustrate how complex, cell-specific BMP signaling mediates niche-stem cell interactions.

## Highlights

• Tkv, a BMP receptor, in the developing ovarian soma controls fertility
• Knockdown Tkv in the developing soma causes ectopic germline stem cell (GSC) accumulation
• Tkv in the soma controls GSC number by limiting BMPs within the GSC niche
• BMP-Tkv signaling in the soma limits GSC number via Egfr and Hh signaling

## Teaser

In this article, Hsu and her colleagues show that Tkv, a type I BMP receptor, functions in the developing ovarian soma to control the size of germline stem cell (GSC) pool via a Smad-independent pathway. BMP-Tkv signaling in the soma limits BMP signals within the GSC niche via Egfr and Hh signaling to ensure that germ cells outside of the niche undergo proper differentiation.

## Introduction

The stem cell niche recruits an appropriate number of stem cells during organogenesis, and maintains stem cell homeostasis throughout the lifespan of an organism (Morrison and Spradling, 2008). However, the mechanisms that regulate the number of stem cells recruited to a niche remain unclear. To further understand this, we used the *Drosophila* ovary as a model because of its relatively simple architecture during developmental and adult stages, as well as its well-characterized germline stem cells (GSCs) and stem cell niche (Fuller and Spradling, 2007, Li and Xie, 2005, Moore et al., 1998).

**Figure 1:**
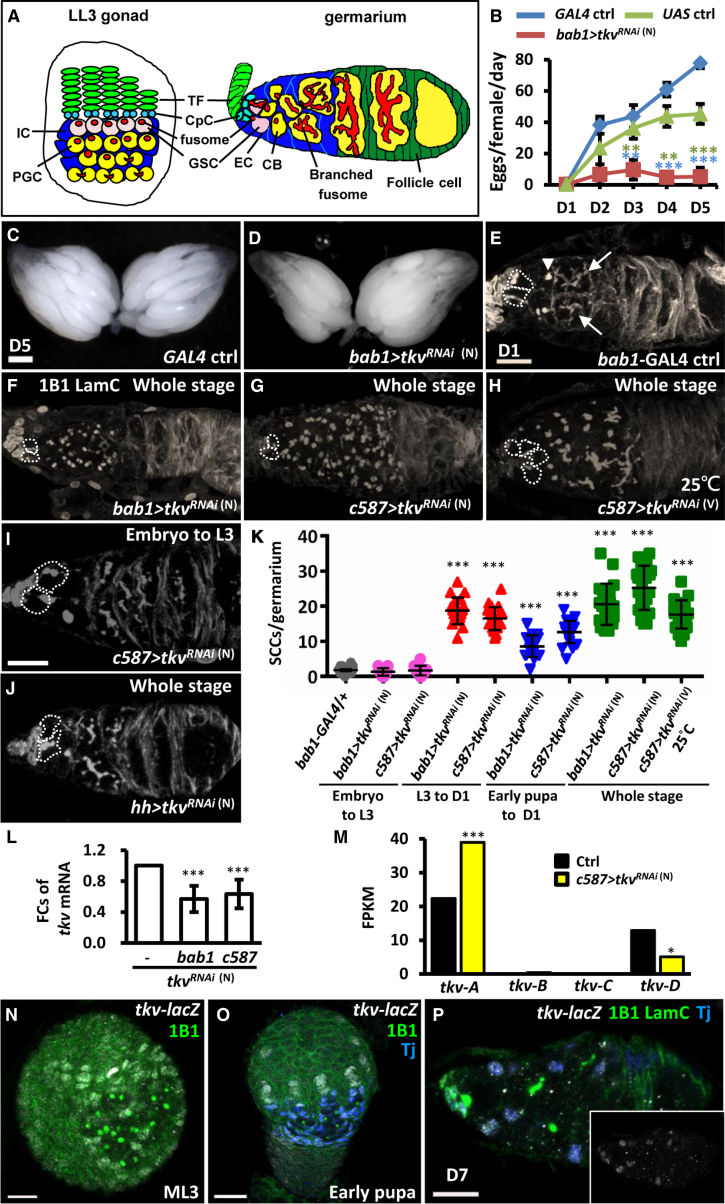
Tkv Expression in the Soma Controls Germ Cell Differentiation for Egg Production (A) Cross-sectional diagrams show a late-L3 (LL3) larval gonad (left) and an adult germarium (right). TF, terminal filament cells; PGC, primordial germ cell containing spectrosomes (round-shaped fusome); IC, intermingled cells; GSC, germline stem cell. PGCs in close proximity to the niche become GSCs, while those further away from the niche initiate differentiation programs (yellow). Dividing PGCs are identified by the presence bar-shaped fusomes. At the end of the LL3 stage, niche cap cells (CpCs, blue) begin to form. During the pupal stage, ICs are incorporated into the germarium and named ECs. GSC progeny, cystoblast (CB) undergoes four rounds of incomplete division to form 16-cell cysts; each cell within the cyst is interconnected with a branched fusome. (B) The average number of eggs produced in a day (D) is shown for newly eclosed *GAL4* control (ctrl), *UAS* control, and *bab1>tkv*^*RNAi*^^(N)^ females from days 1–5. (C and D) Day 5 control (C) and *bab1>tkv*^*RNAi*^^(N)^ (D) ovaries. (E–J) One-day-old *bab1-GAL4* control (E), *bab1>tkv*^*RNAi*^^(N)^ (whole-stage knockdown) (F), *c587>tkv*^*RNAi*^^(N)^ (whole-stage knockdown) (G), *c587>tkv*^*RNAi*^^(V)^ (25°C, whole-stage knockdown) (H), *c587>tkv*^*RNAi*^^(N)^ (embryo to mid-L3 [ML3] knockdown) (I), and *hh > tkv*^*RNAi*^^(N)^ (whole-stage knockdown) (J) germaria with 1B1 (gray, fusomes) and LamC (gray, TF and CpC nuclear envelopes). The arrowhead indicates a spectrosome (round-shaped fusome), and the arrows indicate branched fusomes. (K) Number of spectrosome-containing cells (SCCs) in the germaria of control and *tkvKD* flies driven by *bab1-GAL4* or *c587-GAL4* from embryo to ML3, ML3 to newly eclosed (D1), early pupal to D1 or whole stage. (L) qRT-PCR analysis (fold changes [FCs]) of total *tkv* mRNA in 1-day-old control, *bab1>tkv*^*RNAi*^^(N)^ and *c587>tkv*^*RNAi*^^(N)^ germaria. (M) RNA-seq-based gene expression values (fragments per kilobase of transcript per million mapped reads [FPKM]) for *tkv* isoforms, *tkv-A-D*, in 1-day-old control and *c587>tkv*^*RNAi*^^(N)^ germaria. Statistics analysis was from two biological replicates. (N–P) ML3 (N), early pupa (O), and 7-day-old germarium (P) with *tkv-lacZ* (gray), 1B1 (green), Tj (blue, ICs in O and ECs in P), and LamC (green) labeling. Dashed circles mark GSCs. The insert plane in (P) shows only the *tkv-lacZ* channel. Scale bars, 1 mm (C) and 10 μm (E, I, and N–P). Error bars are SE and in (B) and (L) were from three independent experiments; ^∗^p < 0.05, ^∗∗^p < 0.01, ^∗∗∗^p < 0.001. Knockdown experiments were carried out at 29°C, unless otherwise indicated. Genotypes of control flies are *bab1-GAL4/+* or *UAS-tkv*^*RNAi (N)*^/+.

Each adult ovary contains 16–20 ovarioles, which are the functional units of egg production. The anterior-most structure of the ovariole is called the germarium (Figure 1A, right panel). At the anterior tip of the germarium, a stem cell maintenance niche is formed by terminal filament (TF) cells, cap cells (the major component), and the anterior-most escort cells (ECs). This niche normally supports either two or three GSCs (Kirilly and Xie, 2007). Within each GSC is a special membrane-rich organelle, called the fusome, which is located adjacent to the interface between the GSC and cap cells. Each division of a GSC gives raise a cystoblast (CB), which undergoes four rounds of division to become 2-, 4-, 8-, and then 16-cell cysts. Each cell within the cyst is interconnected via a branched fusome. ECs that do not contact GSCs act as a differentiated cell niche that wraps germ cell cysts with long cellular processes to promote further germ cell differentiation (Kirilly et al., 2011, Morris and Spradling, 2011). Subsequently, cysts become surrounded by a monolayer of follicle cells, bud off from the germarium, and then develop into mature eggs (Margolis and Spradling, 1995).

In the late-third-instar larvae (LL3) stage (Figure 1A, left panel), the GSC niche becomes established within the gonad and recruits GSCs from a pool of virtually identical undifferentiated primordial germ cells (PGCs), which carry rounded fusomes and associate with somatic intermingled cells (ICs). These cells were previously shown to require Hh signaling to maintain interactions with somatic cells (Lai et al., 2017). The anterior-most ICs differentiate into cap cells (Hsu and Drummond-Barbosa, 2011, Lai et al., 2017, Song et al., 2007), which provide E-cadherin-mediated anchorage and stemness signals to select nearby PGCs for the GSC pool (Song et al., 2002). The remaining ICs become ECs that promote PGC differentiation (Lai et al., 2017).

Bone morphogenetic protein (BMP) signaling is highly conserved and controls a variety of developmental processes, as wells as stem cell maintenance during tissue homeostasis (Hamaratoglu et al., 2014, Wang et al., 2014). BMP signals may act through canonical, Smad-dependent, or non-canonical pathways. In the Smad-dependent signaling pathway, BMPs bind to a heterotetrameric complex of type I and type II receptors. After ligand binding, type II receptors transphosphorylate type I receptors, and the activated type I receptors phosphorylate receptor-regulated Smads (R-Smads). Phosphorylated R-Smads will then associate with a common partner (Co)-Smad, and the complex will translocate to the nucleus, where it regulates gene expression. In the *Drosophila* ovary, the BMP homolog, Decapentaplegic (Dpp), is the major niche-derived stemness factor for GSC recruitment and maintenance. GSCs express Saxophone (Sax) and Thickveins (Tkv) as type I receptors and Punt as a type II receptor. To restrict delivery of the Dpp signal to GSCs, niche cap cells also express Division abnormally delayed (Dally), which is a glypican protein that binds and stabilizes Dpp on the extracellular matrix. After binding to receptors on GSCs, the Dpp signal is transmitted to Mothers against Dpp (Mad, R-Smad), which forms a complex with Medea (Med, Co-Smad) to silence transcription of Bag of marbles (Bam), a differentiation factor. While the canonical signaling pathway is the only previously identified mechanism by which Dpp regulates GSCs in the *Drosophila* ovary, BMP signaling is widely known to modulate gene expression in other biological systems via various non-canonical pathways, including the mitogen-activated protein kinase cascade (Wang et al., 2014).

In this study, we found that, in addition to its known role in maintaining GSC identity via Smad signaling, Tkv plays a crucial role in gonadal somatic ICs to confine the Dpp signaling zone for GSC recruitment via a non-Smad pathway during ovary development. Tkv was present in TF and ICs of larval gonads, while in the adult ovary, Tkv was observed in TF, cap and ECs of the germarium. Silencing *tkv* expression in larval gonadal somatic cells resulted in the appearance of ectopic GSCs at the adult; however, gene silencing in TF cells did not. Instead of signaling through BMP canonical proteins, genetic and RNA sequencing (RNA-seq) analyses revealed that ICs expressing Tkv had activated Hh and Egfr signaling in parallel to limit GSC number. As such, evidence of Hh and Egfr signaling was absent in gonads carrying *tkv*-knockdown (*tkvKD*) somatic cells. Moreover, compensatory stimulation of either Hh or Egfr signaling partially prevented the formation of ectopic GSCs in *tkvKD* background, but disruption of either signaling pathway did not affect signaling through the other pathway. Based on these findings, we conclude that Dpp signaling in the GSC niche shapes the Dpp tissue distribution via non-canonical modulation of Hh and Egfr signaling to limit the number of GSCs recruited to the niche.

## Results

### Tkv-Expressing Somatic Cells Promote Germ Cell Differentiation for Functional Reproduction

To identify genes in the soma that control germ cell homeostasis during ovary development, we performed a genetic screen using transgenic *UAS-RNAi* lines from the National Institute of Genetics (N). *RNAi* expression was driven by *bab1-GAL4*, which is expressed in all somatic cells, but specifically in TF cells, cap cells, and anterior ECs at the late pupal and adult stages (Figures S1A, S1C, S1E, S1G, and S1I) (Lai et al., 2017). We found that somatic knockdown of Tkv impaired egg production in the adult (Figure 1B), without affecting overall ovary morphology (Figures 1C and 1D). These results indicate that Tkv-mediated signaling in the soma is required for functional reproduction.

Because no obvious morphological defects were observed in *tkvKD* ovaries, we more closely examined germ cells in somatic *tkvKD* germaria. Control germaria (n = 16) of newly eclosed (D1) flies carried two to three GSCs with anterior anchoring of fusomes that were directly adjacent to niche cap cells. Each germarium also carried 2 ± 1.1 CBs, the immediate daughter cells of GSCs, which were identified by the presence of a spherical fusome (spectrosome-containing cells), but were positioned distal to niche cap cells (Figure 1E). Interestingly, knockdown of *tkv* throughout development using *bab1-GAL4* caused an accumulation of SCCs (20 ± 5.6, n = 20 germaria) (Figures 1F and 1K), suggesting a failure of germ cell differentiation. Similar phenomena were observed with another somatic driver, *c587-GAL4* (Figures 1G and 1K), which was also expressed in almost all somatic cells but restricted in ECs (Figures S1B, S1D, S1F, and S1J), or by an independent *tkv*^*RNAi*^ line (Figures 1H and 1K). However, knockdown of *tkv* before the mid-L3 stage using *bab1* or *c587-GAL4*, or using the TF driver, *hh-GAL4* (Figures S1K and S1L), did not result in SCC accumulation (Figures 1I–1K). Notably, knockdown of *tkv* in the soma throughout development also did not affect GSC and niche cap cell numbers, and egg chamber morphology (Figure S2).

### Tkv-D Functions in ICs to Promote Germ Cell Differentiation

Although qRT-PCR results showed that *tkv* mRNA transcripts were reduced in 1-day-old *bab1>tkv*^*RNAi (N)*^ and *c587>tkv*^*RNAi (N)*^ ovaries (Figure 1L), RNA-seq results revealed that among four *tkv* transcript variants only the level of *tkv-D* transcripts was reduced in somatic *tkvKD* germaria (Figure 1M). Interestingly, *tkv-B* and *tkv-C* expression levels were extremely low, and *tkv-A* mRNA was increased approximately 2-fold in somatic *tkvKD* germaria compared with control (Figure 1M). This result suggests that *tkv-D* may be mainly expressed in the soma, while *tkv-A* may be predominately expressed in germ cells. Furthermore, the germ cell expression of *tkv-A* appears to have somehow been affected by *tkv-D* knockdown in somatic cells. The four different *tkv* transcripts share identical sequences in the coding region and 3′ UTR, while the 5′ UTRs were variable (see Supplemental Information), suggesting that transcription of *tkv-A* and *tkv-D* isoforms may be under the control of different regulatory elements. We further examined *tkv-D* expression during ovary development using a transcriptional reporter, *P2-LacZ*, in which the LacZ reporter is inserted behind the promoter of *Tkv-B*, *-C*, and -*D* (Luo et al., 2015). We found that *P2-lacZ* was highly expressed in TFs and ICs at mid-L3 and early pupal stages (Figures 1N and 1O), but expression was restricted in cap cells and ECs of adult germaria (Figure 1P). These results indicate that Tkv-D functions in ICs for proper germ cell differentiation.

### Disruption of Tkv in the Developing Soma Expands BMP Signaling Territory and Leads to Formation of Ectopic GSCs

**Figure 2:**
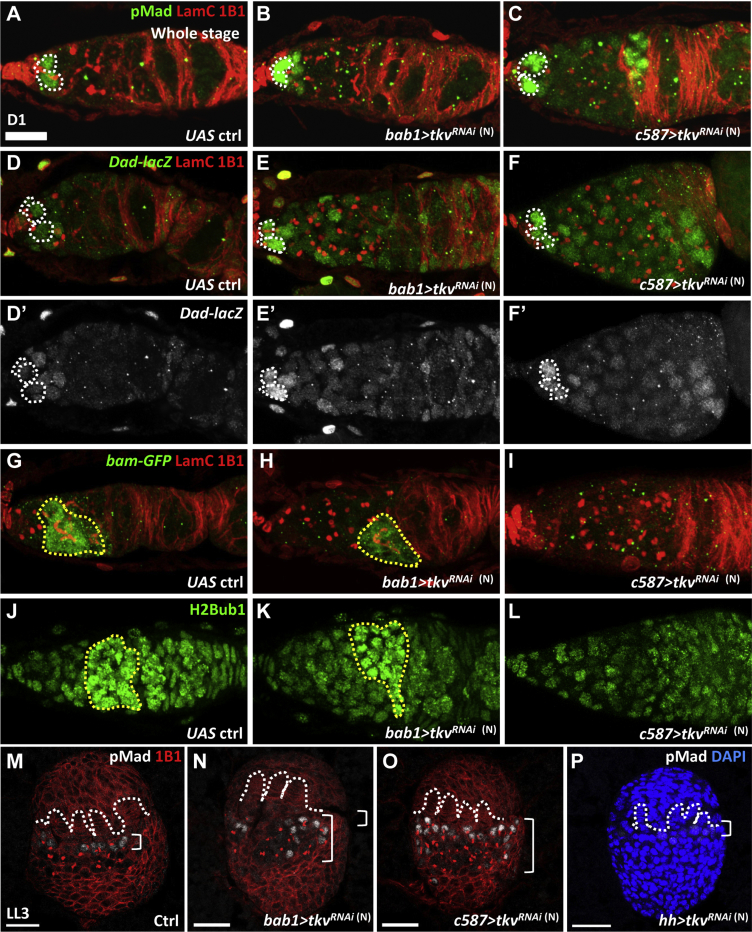
Somatic Tkv Constrains Canonical BMP Signaling to GSCs (A–F) One-day-old control (ctrl) (A and D), *bab1>tkv*^*RNAi*^^(N)^ (B and E), and *c587>tkv*^*RNAi*^^(N)^ germaria (C and F) with 1B1 (red, fusomes), LamC (red, TF and cap cell nuclear envelopes), pMad in (A)–(C) (green), and *Dad-lacZ* in (D)–(F) (green, a BMP signaling reporter). (D′)–(F′) show only *Dad-lacZ* expression in gray. Dashed white circles mark GSCs. (G–I) One-day-old control (G), *bab1>tkv*^*RNAi*^^(N)^ (H), and *c587>tkv*^*RNAi*^^(N)^ germaria (I) with 1B1 (red), LamC (red), and Bam-GFP (green, differentiating cysts marked by dashed yellow circles). (J–L) One-day-old control (J), *bab1>tkv*^*RNAi*^^(N)^ (K), and *c587>tkv*^*RNAi*^^(N)^ germaria (L) with histone H2B mono-ubiquitination (H2Bub1) (green, differentiating cysts marked by dashed yellow circles). (M–P) Late-L3 (LL3) control (M), *bab1>tkv*^*RNAi*^^(N)^ (N), *c587>tkv*^*RNAi*^^(N)^ (O), and *hh > tkv*^*RNAi*^^(N)^ larval gonads (P) with pMad (gray), 1B1 in (G)–(I) (red, fusomes), and DAPI in (J) (blue). Dashed lines mark TFs, brackets indicate the region containing GSCs. Scale bar, 10 μm. The genotype of controls in (A), (D), (G), (J), and (M) is *UAS-tkv*^*RNAi*^^(N)^/+.

We further investigated the fate of ectopic SCCs in somatic *tkvKD* germaria. In GSCs, the binding of BMP ligands induces the phosphorylation of Mad (pMad), which translocates to the nucleus, where it activates expression of *daughter against Dpp* (*dad*) and suppresses *bam* transcription (Harris and Ashe, 2011). Therefore, we examined the levels of pMad, Dad (revealed by *dad-lacZ*), and Bam (revealed by *bam::bam-GFP* [Chen and McKearin, 2003]) in 1-day-old control and *bab1>tkv*^RNAi (N)^ and *c587>tkv*^*RNAi*^ ^(N)^ germaria. In the control germaria (Figure 2A), pMad was observed in GSCs (Figure 2A), while, in somatic *tkvKD* germaria, pMad expression levels were increased in GSCs and also detected in germ cells outside the niche (Figures 2B and 2C). Similarly, *dad-lacZ* expression was high in GSCs and CBs in the control germarium (Figure 2D), but, in somatic *tkvKD* germaria, expression was greatly increased in GSCs and detected in most germ cells (Figures 2E and 2F). In both control and somatic *tkvKD* germaria, differentiated germ cells with branched fusomes exhibited *bam-GFP* expression, but this expression was absent in SCCs from somatic *tkvKD* germaria (Figures 2G–2I). It was previously reported that monoubiquitinated histone H2B (H2Bub1) is absent from GSCs and pre-CBs (König and Shcherbata, 2015). To further characterize the aberrant SCCs in somatic *tkvKD* germaria, we examined the expression of H2Bub1. Surprisingly, in control germaria, we detected H2Bub1 expression in GSCs, and its expression reached the highest levels in the differentiating germline (Figure 2J). In somatic *tkvKD* germaria (Figures 2K and 2L), GSCs and germ cells outside of the niche displayed similar H2Bub1 expression levels. These results indicate that the SCCs accumulated in *tkvKD* germaria may be GSCs or immediate GSC progeny with active BMP signaling and suppressed Bam expression maintaining them in an undifferentiated state.

We also observed an expansion of pMad-positive germ cells in *bab1>tkv*^RNAi (N)^ and *c587>tkv*^*RNAi*^ ^(N)^ ovaries at the LL3 stage (Figures 2N and 2O), compared with control or *hh > tkv*^*RNAi*^ gonads (Figures 2M and 2P). These results suggest that BMP signaling is elevated and spreads to germ cells outside of the niche when Tkv is eliminated from ovarian somatic cells during development.

### SCC Accumulation Is Not Due to Excessive Dpp Production

**Figure 3:**
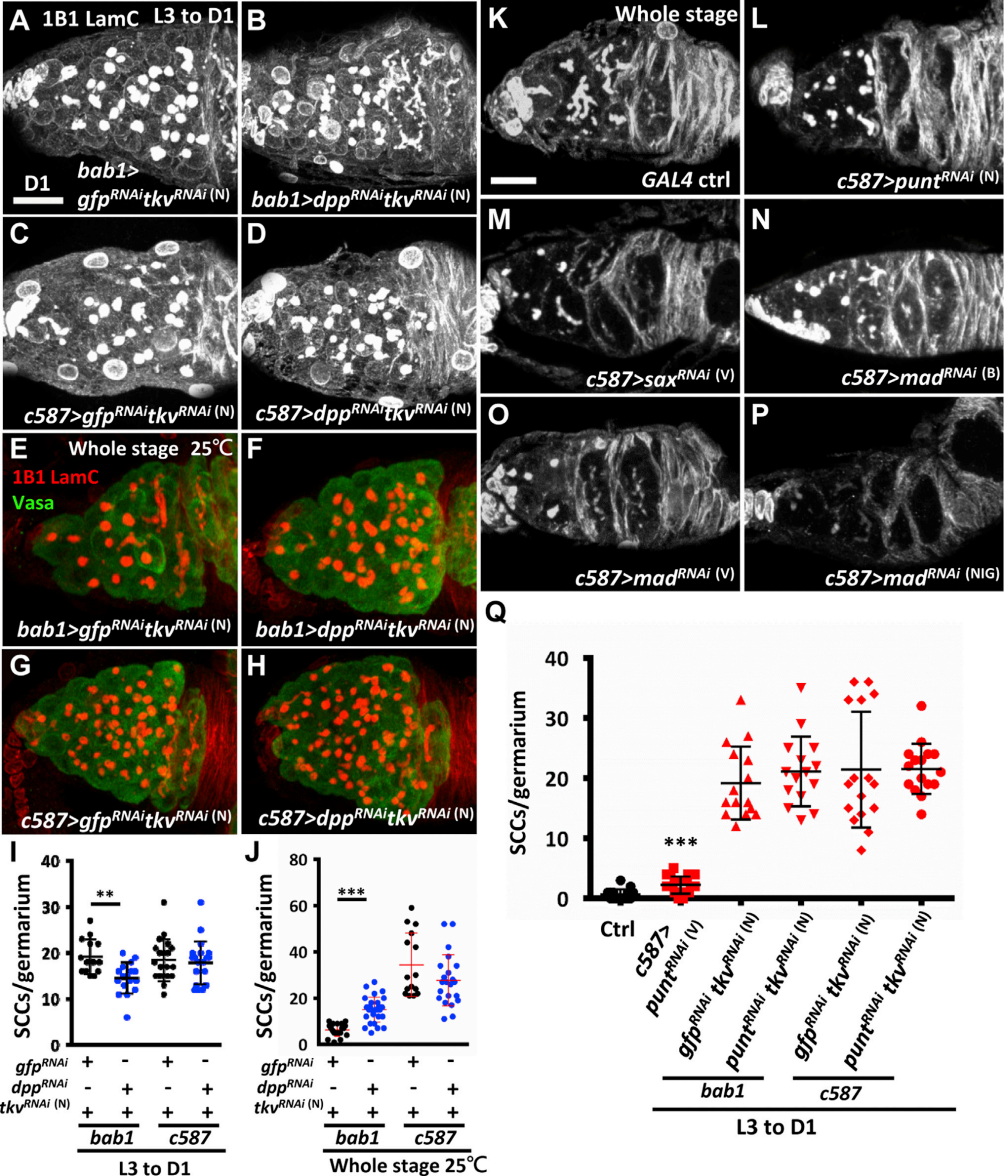
Dpp-Tkv-Mediated Signaling in the Soma Limits GSC Number via a Smad-Independent Pathway (A–D) One-day-old germaria with *gfp*^*RNAi*^*tkv*^*RNAi*^^(N)^ (A and C) and *dpp*^*RNAi*^*tkv*^*RNAi*^^(N)^ (B and D) knockdown by *bab1-GAL4* (A and B) or *c587-GAL4* (C and D) from ML3 to adult (D1) stage. Germaria were stained for 1B1 (gray, fusomes) and LamC (gray, TF and cap cell nuclear envelopes). Co-knockdown of *dpp* and *tkv* in the niche by *bab1-GAL4* reduced the number of SCCs. (E–H) One-day-old germaria with *gfp*^*RNAi*^*tkv*^*RNAi*^^(N)^ (E and G) and *dpp*^*RNAi*^*tkv*^*RNAi*^^(N)^ (F and H) knockdown by *bab1-GAL4* (E and F) or *c587-GAL4* (G and H) throughout development at 25°C. Germaria were stained for 1B1 (red), LamC (red), and Vasa (green, germ cells). (I and J) Number of SCCs per germarium in the indicated RNAi knockdown genotypes, either from ML3 to D1 (I) or throughout all developmental stages (25°C) (J). (K–P) One-day-old control (ctrl) germaria (K) and those with *punt*^*RNAi*^^(L)^ (L), *Sax*^*RNAi*^^(V)^ (M), and *mad*^*RNAi*^^(B, V, and NIG)^ (N–P) knockdown by *c587-GAL4* throughout all developmental stages. Germaria were stained for 1B1 and LamC as in (A)–(D). (Q) Number of SCCs per germarium in the indicated RNAi knockdown genotypes from ML3 to D1. Knockdown experiments were carried out at 29°C, except when otherwise indicated. The genotype of the control in (K) and (Q) is *c587-GAL4/+*. Scale bars, 10 μm. Error bars indicate SE; ^∗∗^p < 0.01, ^∗∗∗^p < 0.001.

Because of the widespread BMP signaling in the accumulated SCCs, we sought to test whether Dpp, a major BMP ligand, was upregulated in *tkvKD* ovaries. Therefore, we examined *dpp* expression in control and *c587>tkv*^*RNAi*^ ^(N)^ or *bab1>tkv*^*RNAi*^ ^(N)^ germaria by qRT-PCR, RNA-seq, and a *dpp* transcriptional reporter, *dpp2.0-lacZ* (Luo et al., 2017). By all three methods, we found that *dpp* expression was decreased in *tkvKD* ICs and adult cap cells (Figures S3A–S3H). We also disrupted *dpp* expression in somatic *tkvKD* germaria using a *dpp*^*RNAi*^ line. Functional *dpp* knockdown in this line was validated by pMad staining (Figures S3I and S3J). Co-knockdown of *dpp* and *tkv* by *c587-GAL4* did not reduce the number of SCCs, and co-knockdown of *dpp* and *tkv* by *bab1-GAL4* only slightly reduced SCC number (Figures 3A–3D and 3I). Thus, we conclude that elevation of Dpp levels is not a primary cause of SCC accumulation in somatic *tkvKD* germaria, although Dpp may be distributed outside of the niche. Further, the differential expression of cell types between the two *GAL4* lines to rescue SCC accumulation supports the previously proposed model that niche cap cells are the major source of Dpp production (Liu et al., 2015), regardless of whether the expression of *dpp 2.0-lacZ* is present in ICs of larval gonads (see Figure S3).

### Non-canonical Dpp-Tkv Signaling in the Soma Promotes Germ Cell Differentiation

In the canonical BMP signaling pathway, Punt or Sax forms a complex with Tkv and transmits Dpp signals to Mad by phosphorylation (Hamaratoglu et al., 2014). To dissect the Tkv-mediated signaling pathway that functions in the soma to promote germ cell differentiation, we first asked if Dpp is required. We moderately reduced *dpp* and *tkv* expression by driving *RNAi* expression at a lower temperature (25°C instead of 29°C). Knockdown of *tkv* alone, using *bab1-GAL4*, still resulted in few SCCs (6.2 ± 2.7, n = 20). Interestingly, simultaneous knockdown of *tkv* and *dpp* using *bab1-GAL4* dramatically increased the number of SCCs (15.1 ± 5.4, n = 31, p < 0.001) (Figures 3E, 3F, and 3J), which were p-Mad-positive germ cells (Figures S3K–S3M). This synergistic effect on SCC accumulation suggests that the two factors function in the same general pathway. In contrast, disruption of *tkv* with or without *dppKD* using *c587-GAL4* did not produce any differences in SCC number (Figures 3G, 3H, and 3J). This result indicates that Dpp is mainly produced by niche cap cells, and activates Tkv in the soma to promote germ cell differentiation. We also disrupted expressions of *punt*, *sax*, or *mad* in the soma. Knockdown of *punt* only resulted in a small increase of SCC number, compared with controls (Figures 3K, 3L, and 3Q); co-knockdown of *punt* and *tkv* in the soma also appeared to have slightly increased SCC number, but the difference did not reach statistical significance. In addition, knockdown of *sax* or *mad* did not produce observable SCC accumulation (Figures 3M–3P). Since the accumulation of SCCs was dependent on *dpp* expression, but not the expression of canonical signaling molecules, the results suggest that Dpp-Tkv signaling controls germ cell differentiation via a non-canonical BMP signaling mechanism.

### Tkv Restricts GSC Recruitment via the Egfr-Dally Regulatory Axis

**Figure 4:**
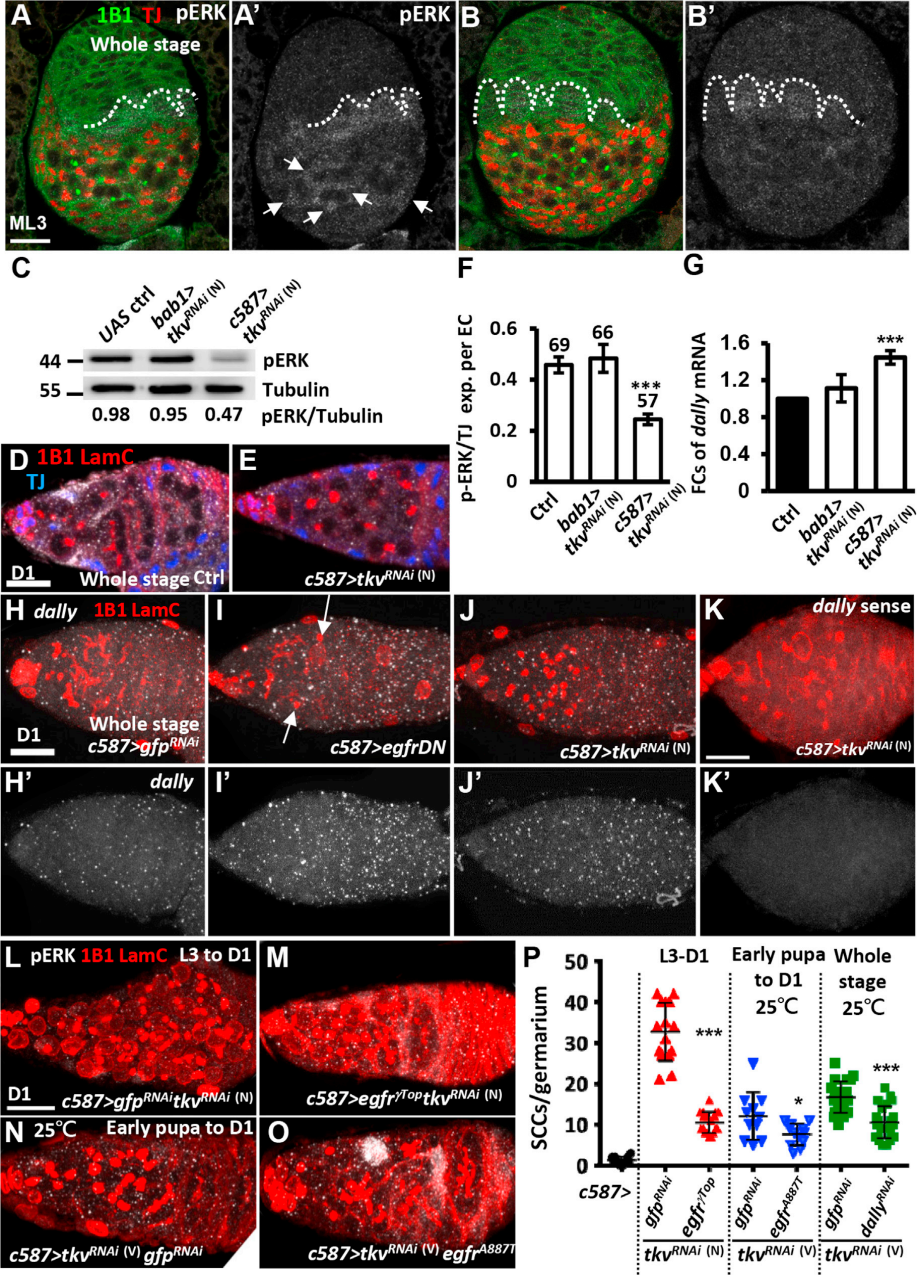
Dpp Signaling in the Soma Limits the GSC Number via Egfr Signaling (A and B) ML3 control (ctrl) (A) and *c587>tkv*^*RNAi*^^(N)^ (B) gonads with 1B1 (green, fusomes), Tj (red, ICs), and phospho (p)ERK (gray). Dashed line indicates TFs. (A′) and (B′) show only pERK channel. Arrows in (A) point to ICs with strong pERK signals. (C) Representative pERK (42/44 kDa) western blots of control, *bab1>tkv*^*RNAi*^^(N)^, and *c587>tkv*^*RNAi*^^(N)^ ML3 gonads. α-Tubulin (55 kDa) was used as an internal control. Molecular weight markers are indicated to the left of the blots. Ratio of pERK to α-tubulin expression is shown below the blot. (D and E) One-day-old control (ctrl) and *c587>tkv*^*RNAi*^^(N)^ germaria with 1B1 (red), LamC (red, cap cell nuclear envelopes), Tj (blue, ECs), and pERK (gray) labeling. (F) Ratio of pERK to Tj expression per EC is shown for 1-day-old control, *bab1>tkv*^*RNAi*^^(N)^, and *c587>tkv*^*RNAi*^^(N)^ flies. Number of ECs analyzed are shown above each bar. (G) qRT-PCR analysis revealed FCs of *dally* mRNA in 1-day-old control, *bab1>tkv*^*RNAi*^^(N)^, and *c587>tkv*^*RNAi*^^(N)^ germaria. (H–K) One-day-old *c587>gfp*^*RNAi*^ (H), *c587>egfrDN* (I), and *c587>*t*kv*^*RNAi*^^(N)^ germaria (J and K) with 1B1 (red), LamC (red), anti-sense *dally* RNA probe in (H)–(J) (gray), and sense *dally* probe in (K) (gray). Arrows point to spectrosomes. In (H′)–(K′) only the *in situ* staining for *dally* is shown. (L–O) One-day-old *c587>gfp*^*RNAi*^*tkv*^*RNAi*^^(N)^ (L), *c587>egfr*^*γTop*^*tkv*^*RNAi*^^(N)^ (M), *c587> tkv*^*RNAi*^^(V)^*gfp*^*RNAi*^ (N), and *c587> tkv*^*RNAi*^^(V)^*egfr*^*A887T*^ germaria (O) with 1B1 (red), LamC (red), and pERK (gray). (P) Number of SCCs in control germaria and those expressing *tkv*^*RNAi*^ plus gfp^RNAi^, *egfr*^*γTop*^, *egfr*^*A88T*^, or *dally*^*RNAi*^ driven by *c587-GAL4* from ML3 to D1, pupa to D1 stages, or throughout development (whole stage). Knockdown experiments were carried out at 29°C, except where otherwise indicated. The genotype of the control in (A) and (D) is *c587-GAL4/+*. Scale bars, 20 μm (A) and 10 μm (D, H, K, and L). Error bars show SE and in (G) is from at least three independent experiments: ^∗^p < 0.05, ^∗∗∗^p < 0.001.

Egfr signaling in ICs negatively regulates the number of PGCs in order to balance the soma and germ cell populations (Gilboa and Lehmann, 2006). In addition, Egfr signaling is known to suppress expression of *dally* (encodes a glypican that facilitates distribution of Dpp) to sharpen the BMP gradient in the larval gonad, such that the first row of PGCs (next to TFs) will be recruited and maintained as GSCs (Guo and Wang, 2009, Matsuoka et al., 2013). We found that somatic *tkvKD* gonads in mid-L3 exhibited a small increase in PGC number (Figures S4A–S4E), without obvious changes in the number of ICs (Tj-positive cells in Figures 4A and 4B). Egfr signaling, as indicated by phospho (p)ERK staining (Gabay et al., 1997), was reduced in ICs of mid-L3 *c587>tkv*^*RNAi*^ ^(N)^ gonads (Figures 4A–4C and S4F–S4H), and in ECs of the adult *c587>tkv*^*RNAi*^ ^(N)^ germarium (Figures 4D–4F). Surprisingly, pERK expression was not affected in *bab1>tkv*^*RNAi*^ ^(N)^ ovaries (Figures 4C, 4F, and S4F–S4H). We speculate that this difference was due to *GAL4* expression differences between the two lines, as expression of *c587-GAL4* was higher in ICs from late-L3 to adult stages when compared with *bab1-GAL4* (see Figure S1). In contrast, overexpressing a constitutively active form of *tkv* (*tkv*^*CA*^) in somatic gonadal precursors after the mid-L3 stage driven by either *bab1-* or *c587-GAL4* caused an elevation of Egfr signaling in adult ECs (Figures S5A–S5C). In addition, germaria with *tkv*^*CA*^ overexpression, driven by either *bab1-GAL4* or *c587-GAL4* from mid-L3 to adult (data not shown) or throughout development (Figures S5D–S5I), were devoid of GSCs and exhibited an associated loss of cap cells. This observation was in agreement with the idea that high Egfr signaling depletes the PGC pool (Matsuoka et al., 2013). However, the reduction of cap cells may also contribute to the observed loss of GSCs. Nevertheless, this result suggests that Tkv-mediated signaling restricts GSC number, at least partially through Egfr signaling.

As expected, *dally* transcript levels, which are negatively regulated by Egfr signaling (Liu et al., 2010), were increased in *c587>tkv*^*RNAi*^ ^(N)^ ovaries as compared with control and *bab1>tkv*^*RNAi*^ ^(N)^ (Figure 4G). We confirmed this qRT-PCR result with *in situ* hybridization in 1-day-old germaria. Compared with control (Figure 4H), *dally* mRNA was increased in the germaria that overexpressed a dominant-negative form of Egfr (*egfr*^*DN*^) or *tkvKD* driven by *c587-GAL4* (Figures 4I and 4J). Probing *c587>tkv*^*RNAi*^ ^(N)^ germaria with *dally* sense probes did not show any signal (Figures 4K), confirming the specificity of *dally* anti-sense probes used in this experiment.

To directly test if Egfr signaling acts downstream of Tkv to limit GSC number, we knocked down *tkv* with concurrent overexpression of a constitutively active form of Egfr, *egfr*^*λTop*^ (Queenan et al., 1997) with *c587-GAL4* from L3 to adult stages, and examined the SCC number in 1-day-old germaria (Figures 4L, 4M, and 4P). Our results showed that SCC accumulation was prevented by forcing Egfr signaling (Figure 4P). Knockdown of *tkv* with coincident overexpression of another constitutive active form of Egfr, *egfr*^*A887T*^ (Lesokhin et al., 1999), from pupal to adult stages (Figures 4N and 4O), or co-knockdown of *tkv* and *dally* throughout developmental stages by *c587-GAL4* also significantly reduced SCC number (Figure 4P). These results demonstrate that Tkv signaling in the ovarian soma promotes Egfr signaling to suppress Dally expression and thereby shape the localization of Dpp signals to limit GSC number.

### Somatic Tkv Signaling Promotes Germ Cell Differentiation via Hh Signaling

**Figure 5:**
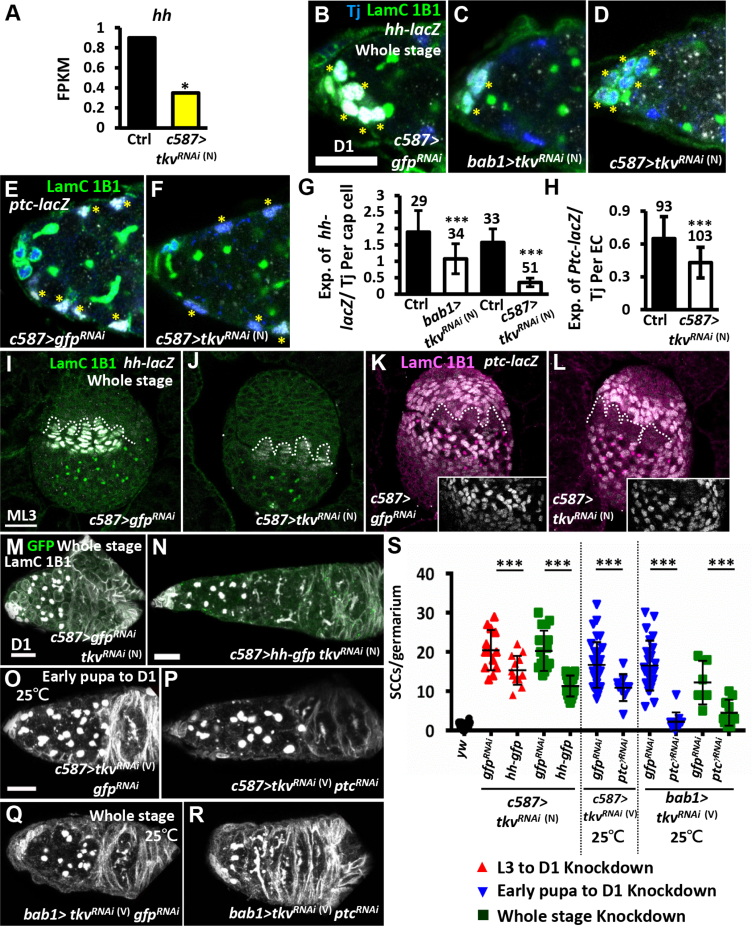
Dpp Signaling in the Soma Limits GSC Number via Hh Signaling (A) RNA-seq-based gene expression values (FPKM) for *hh* in the 1-day-old control and *c587>tkv*^*RNAi*^^(N)^ germaria. Statistics analysis was from two biological replicates. (B–F) One-day-old *c587>gfp*^*RNAi*^ (B and E), *bab1-tkv*^*RNAi*^^(N)^ (C), and *c587>tkv*^*RNAi*^^(N)^ germaria (D and F) with staining for 1B1 (green, fusomes), LamC (green, cap cell nuclear envelopes), Tj (blue, ECs), *hh-lacZ* in (B–D) (gray), and *ptc-lacZ* in (E and F) (gray). Asterisks in (B)–(D) indicate cap cells, and in (E) and (F) indicate ECs. (G) Ratio of *hh-lacZ* to Tj in cap cells of control, *bab1-tkv*^*RNAi*^^(N)^, and *c587>tkv*^*RNAi*^^(N)^ germaria. (H) Ratio of *ptc-lacZ* to Tj in ECs of control and *c587>tkv*^*RNAi*^^(N)^ germaria. Number of cells analyzed are shown above each bar. (I–L) ML3 *c587>gfp*^*RNAi*^ (I and K) and *c587>tkv*^*RNAi*^^(N)^ gonads (J and L) with 1B1 and LamC, green in (I and J), magenta in (K and J), *hh-lacZ* (gray) in (I) and (J), and *ptc-lacZ* (gray) in (K) and (L). Dashed line indicates TFts; inserts show intermingled cell regions with only the *ptc-lacZ* channel. (M–R) One-day-old germaria expressing *gfp*^*RNAi*^*tkv*^*RNAi (N)*^ (M), *hh-gfp tkv*^*RNAi*^^(N)^ (N), *tkv*^*RNAi (V)*^*gfp*^*RNAi*^ (O and Q), and *tkv*^*RNAi (V)*^*ptc*^*RNAi*^ (P and R) driven by *c587-GAL4* (M–P) or *bab1-GAL4* (Q and R). Germaria with GFP (green), 1B1, and LamC (gray) are shown. (S) Number of SCCs per germarium at day 1 of flies with indicated *RNAi* expression from ML3 to D1, early pupa to D1 stages, or throughout development (whole stage). Knockdown experiments were carried out at 29°C, except where otherwise indicated. The genotype of the control in (A) is *UAS-tkv*^*RNAi*^^(N)^*/+*. The genotypes of the controls in (E and H) are *bab1-GAL4/+* or *c587-GAL4/+*. Scale bars, 10 μm (B, M, N, and Q) and 20 μm (I). Error bars indicate SE; ^∗^p < 0.01, ^∗∗∗^p < 0.001.

Knockdown of *tkv* using *bab1-GAL4* results in SCC accumulation but does not alter Egfr signaling, suggesting that additional Tkv-downstream effectors independently control germ cell differentiation. By analyzing RNA-seq results, we found that transcripts of *hh* were significantly reduced in *c587>tkv*^*RNAi*^ ^(N)^ germaria (Figure 5A), reminiscent of a study that showed Hh signaling functions in ECs for germ cell differentiation (Lu et al., 2015). We verified that Hh signaling was reduced using *hh-lacZ*, a transcriptional reporter (Forbes et al., 1996, Lai et al., 2017), and *ptc-lacZ*, an Hh signaling reporter (Chen and Struhl, 1996, Lai et al., 2017). Results showed that *hh-lacZ* was significantly decreased in niche cap cells of somatic *tkvKD* germaria at day 1 (Figures 5B–5D and 5G). Consistently, *ptc-lacZ* was also dramatically reduced in ECs of somatic *tkvKD* germaria (Figures 5E, 5F, and 5H). Reductions of *hh-lacZ* and *ptc-lacZ* expression were also observed in TFs and ICs, respectively, of mid-L3 gonads from somatic *tkvKD* larvae (Figures 5I–5L). The reduction of Hh signaling was not due to decreased canonical Dpp signaling in the soma, as *ptc-lacZ* expression levels in ECs were comparable in control and the germaria with somatic *mad*-knockdown from the L3 to adult stages (Figures S6A and S6B). Disruption of Hh signaling from L3 to adult stages, by knockdown of *hh* or *smoothened* (*smo*, the Hh receptor) in the soma, also caused SCC accumulation (Figures S6C–S6L). These results suggest the involvement of Hh signaling in the developing soma for germ cell differentiation.

It has been reported that Hh signaling suppresses *dpp* transcription in adult ECs and promotes EC membrane extension to force germ cell differentiation (Huang et al., 2017, Lu et al., 2015). We observed defective membrane extension by ECs (derived from ICs) in germaria of newly eclosed flies (Figures S6M–S6O). However, we did not see increased *dpp* transcripts in ECs; instead, *dpp* transcript levels were reduced in cap cells of *bab1>tkv*^*RNAi*^ ^(N)^ ovaries (see Figures S3G and S3H), which was in agreement with studies that show that Hh signaling regulates *dpp* transcription in the wing disc (Aza-Blanc et al., 1997).

We directly tested the role of Hh signaling in this process by supplying Hh to somatic *tkvKD* gonads and subsequently examining SCC number in 1-day-old germaria. We found that expression of Hh-GFP fusion protein in the *tkvKD* gonads either from L3 to adult stages, or throughout development, significantly reduced SCC accumulation compared with germaria with *tkvKD* alone (Figures 5M, 5N, and 5S). Forcing Hh signaling in *c587>tkv*^*RNAi (N)*^ gonads by knockdown of *ptc* (encodes a Smo suppressor) from early pupal to adult stages, or during all developmental stages, also partially suppressed SCC accumulation (Figures 5O, 5P, and 5S). Notably, neither overexpression of Egfr nor activation of Hh signaling in *c587>tkv*^*RNAi*^ gonads could completely suppress SCC accumulation (see also Figure 4), implying that both Hh and Egfr signaling are required downstream of Tkv for germ cell differentiation. Some germaria with *bab1-GAL4*-driven *ptc* and *tkv* co-knockdown from early pupal to adult stages, or throughout all developmental stages, exhibited SCC numbers that were comparable with those of *gfpKD* controls (Figures 5Q–5S). This result implies that defective Hh signaling is primarily responsible for SCC accumulation in *bab1>tkv*^*RNAi*^ ^(V)^ ovaries, wherein Egfr signaling was not affected. We also noticed that the blunted EC membrane protrusions in somatic *bab1>tkv*^*RNAi*^ ^(V)^ germaria were rescued by *ptcKD* (Figures 5Q and 5R). Together, these results show that Hh signaling in the soma is controlled by Tkv and promotes membrane extension of ECs for germ cell differentiation. At the same time, Tkv-mediated Hh signaling upregulates somatic *dpp* expression in niche precursors, forming a positive feedback loop to enhance the Dpp signal.

### Egfr and Hh Signaling Are Regulated in Parallel by Tkv in the Soma

**Figure 6:**
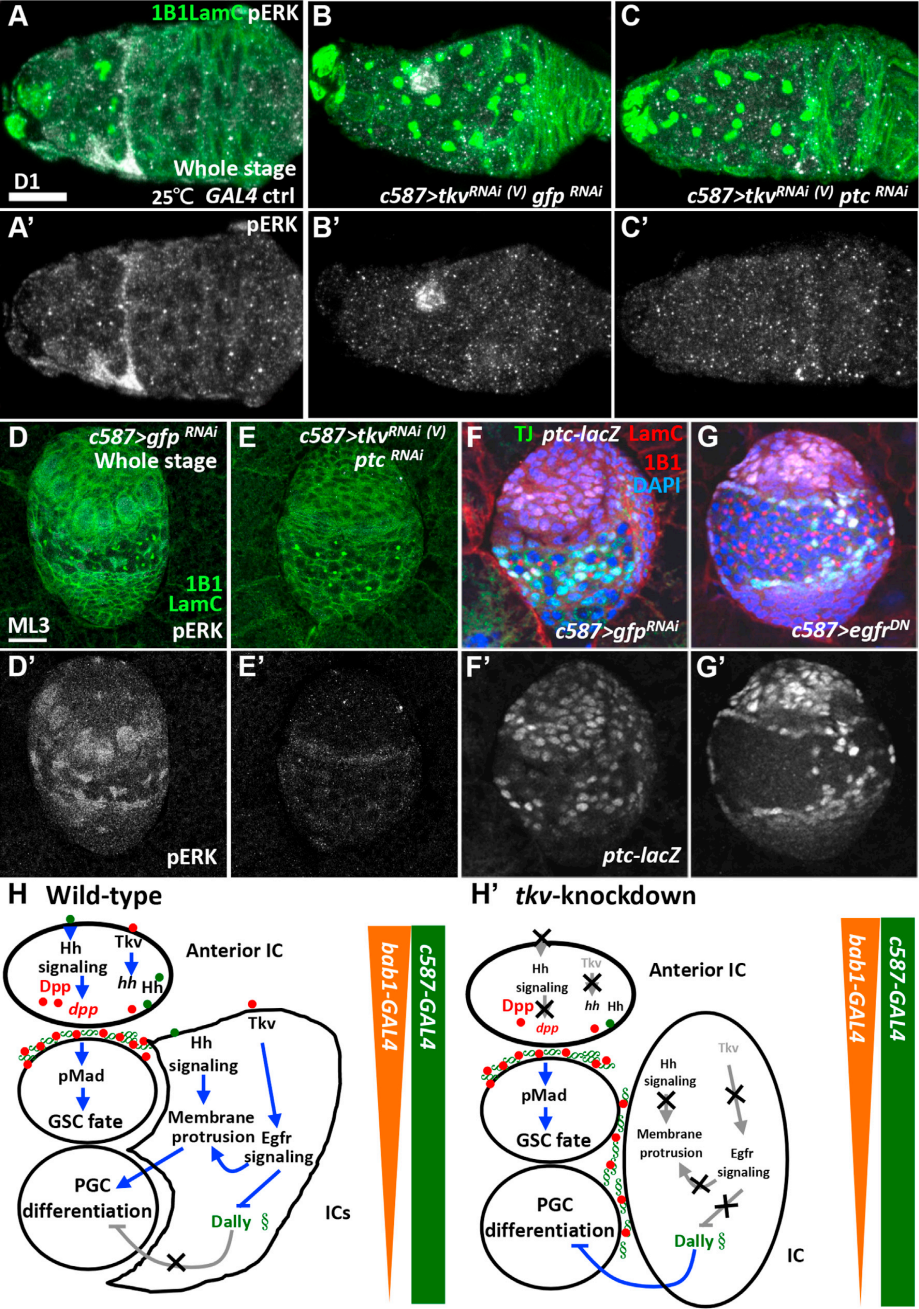
Somatic Tkv Controls Hh and Egfr in Parallel to Maintain GSC Number (A–C) One-day-old control (ctrl) (A), *c587>tkv*^*RNAi*^^(V)^*gfp*^*RNAi*^ (B) and *c587>tkv*^*RNAi*^^(V)^*ptc*^*RNAi*^ germaria (C) with 1B1 (green, fusomes), LamC (green, cap cell nuclear envelopes) and pERK (gray) labeling. Only pERK staining is shown in (A′)–(C′). (D and E) ML3 *c587>gfp*^*RNAi*^ (D) and *c587>tkv*^*RNAi*^^(V)^*ptc*^*RNAi*^ gonads (E) with 1B1 (green), LamC (green), and pERK (gray) labeling. (D′) and (E′) show pERK channel only. (F and G) ML3 *c587>gfp*^*RNAi*^ (F) and *c587>egfr*^*DN*^gonads (G) with staining for 1B1 (red), LamC (red), Tj (green, ICs), *ptc-lacZ* (gray), and DAPI (blue, DNA). (F′) and (G′) show *ptc-lacZ* channel only. Knockdown experiments were carried out at 29°C, except where otherwise indicated. Whole stage (throughout development) indicates the time window for transgene expression. The genotype of the control in (A) is *c587-GAL4/+*. Scale bars, 10 μm (A) and 20 μm (D). (H and H′) Model of somatic Tkv regulation of Hh and Egfr signaling to limit the GSC pool. In the wild-type larval ovary (H), Tkv-mediated signaling elevates *hh* transcripts and activates Hh signaling in anterior of ICs to enhance Dpp expression, which strengthens Tkv-mediated signaling in both GSCs and somatic cells. Hh signaling is also activated in posterior ICs to drive PGC differentiation, at least in part by promoting membrane extension of ICs. Tkv-mediated signaling in ICs also positively regulates Egfr signaling, which diminishes Dally transcripts to broaden Dpp distribution and promotes IC membrane extension. In the somatic *tkvKD* larval ovary (H′), Hh and Egfr signaling is attenuated, and Dally is upregulated, thereby expanding Dpp signal distribution and leading to overpopulation of GSCs. *bab1-GAL4* is strongly expressed in anterior of gonadal somatic cells but gradually restricted in the GSC niche; *c587-GAL4* is expressed in all somatic cells but gradually exclusively expressed in ECs.

We next asked if Egfr and Hh signaling crosstalk to control GSC number. Egfr signaling activity, as revealed by pERK staining, was strongly present in ECs of controls (Figure 6A) and dramatically reduced in the somatic *tkvKD* germarium (Figure 6B), but pERK levels were not rescued in germaria with *tkv* and *ptc* co-knockdown throughout developmental stages (Figure 6C). A similar set of observations were made in mid-L3 somatic *tkvKD* gonads with or without *ptcKD* (Figures 6D and 6E). Correspondingly, Hh signaling activity, as revealed by *ptc-lacZ*, was not altered in Egfr signaling-defective gonads (Figures 6F and 6G). Thus, we conclude that Tkv signaling in ICs independently regulates Hh and Egfr signaling to limit GSC number by stimulating differentiation in PGCs that are not selected as GSCs by the niche.

## Discussion

A properly sized stem cell pool is critical for maintaining tissue homeostasis, but the mechanisms that regulate the number of stem cells in a niche are not fully understood. Here, we report that non-canonical BMP signaling independently promotes Hh and Egfr signaling in the developing soma to restrict BMP signaling territory for GSC specification. In the developing ovary (Figure 6H), *dpp* mRNA is mainly produced by anterior somatic gonadal cells (Zhu and Xie, 2003), including niche precursors, such as TF cells and anterior ICs. Coincident expression of Dally is highly expressed in the extracellular matrix of niche precursors, and helps to enrich the Dpp signal within the niche (Matsuoka et al., 2013). Dpp signaling is then activated through a canonical Smad-dependent pathway in PGCs that are adjacent to the niche, allowing the cells to adopt a GSC fate. Dpp signal is also received in Tkv-expressing somatic cells, but coordinates with Egfr and Hh signaling to constrain Dpp signals located in the niche. In ICs, Tkv activates Egfr signaling to suppress Dally, limiting the spread of Dpp signals outside of the niche. In addition, Egfr signaling is also known to promote EC cellular protrusion, which contributes to germ cell differentiation (Banisch et al., 2017). On the other hand, Tkv-mediated signaling promotes *hh* transcription in anterior ICs (putative niche precursors [Lai et al., 2017]). This action promotes Hh signaling, which has the dual effects of controlling EC membrane extension to facilitate germ cell differentiation. In anterior ICs, Hh signaling promotes Dpp expression to strengthen Tkv signaling. When Tkv is eliminated from the soma (Figure 6H′), Egfr signaling in ICs is reduced, resulting in the upregulation of Dally and a subsequent loss of control over Dpp distribution. At the same time, Hh signaling is reduced in niche precursors and ICs, which diminishes Dpp expression in the niche precursors and blunts membrane extension in ICs (called ECs only after germarium is formed). These reductions of Egfr and Hh signaling in the developing soma result in the formation of ectopic GSCs in the germarium.

### The *Drosophila* Ovarian Soma Utilizes Non-canonical Dpp Signaling to Restrict the Boundary of Niche Activity

Stemness factors must be restricted to the niche during organ development to recruit and maintain an appropriate number of stem cells. Dpp can act as a long-range morphogen and is produced by the niche at high levels, but the signal is only intended for neighboring GSCs, located up to one-cell diameter from the niche. It has been previously shown that Egfr signaling in ICs diminishes Dpp signaling outside the niche (Matsuoka et al., 2013). In this process, PGCs produce Spitz, which activates Egfr signaling in ICs to suppress expression of Dally. Since Dally coordinates Dpp signals to localize on the cell surface, Dpp signaling is reduced outside of the niche when Dally is suppressed (Guo and Wang, 2009, Matsuoka et al., 2013). Here, we report that Tkv, encoded by the *tkv-A* isoform, induces canonical Dpp signaling in PGCs within the niche to specify a GSC fate, while *tkv-D*-encoded Tkv mediates non-canonical Dpp signaling through Egfr and Hh in the soma to prevent Dpp leaking from the niche. Although we do not know how Tkv affects *hh* transcription, our genetic data suggest that Tkv acts upstream of Egfr, since overexpressing a constitutively active form of Egfr suppressed *tkvKD*-induced SCC accumulation. However, our RNA-seq results revealed that transcript levels of *egfr* and genes encoding Egfr ligand (Spitz) (Tio et al., 1994) and modulators (Argos and Gone early) (Klein et al., 2004, Matsuoka et al., 2014) were not affected in somatic *tkvKD* ovaries. We speculate that non-canonical Dpp signaling may control Egfr at a post-transcriptional level, or it may influence the competence of ICs to respond to the Spitz signal.

Luo et al. (2015) previously reported that Tkv acts as a receptor sink in ECs of the adult germaria (derived from ICs) to sequester excess Dpp that is found outside the niche. Similar to our findings, components of canonical Dpp signaling were reported to be unnecessary in this process. However, there are several key differences that make the two studies complementary, providing a more nuanced understanding of the role of Tkv in defining the niche. First, the timing of *tkv* knockdown was different. Luo et al. suppressed *tkv* expression in ECs at the adult stage, while we manipulated *tkv* expression in the soma during developmental stages. Second, the molecular action of Tkv in limiting GSC number is different. In the previous study, knockdown of *tkv* in adult ECs did not affect Egfr signaling or *dally* expression, and overexpressing the extracellular domain of Tkv significantly reduced SCC number in *tkv*-knockdown germaria. Therefore, the authors concluded that expression of Tkv in adult ECs serves as a sink to sequester Dpp signals that may spread outside of the niche. In contrast, our results showed that Egfr signaling is dramatically reduced, and that *dally* becomes ectopically expressed in ICs/ECs when *tkv* expression is eliminated in the ovarian soma during development. These events were shown to be functionally important, because activation of Egfr signaling or knockdown of *dally* expression in the *tkvKD* ovarian soma during development were sufficient to reduce SCC number. Lastly, we also found that, in the developing soma, Tkv promoted Hh signaling, which may control membrane extension of ECs for germ cell differentiation. Knockdown of *tkv* in the soma of larval ovaries resulted in decreased Hh signaling, and forcing Hh signaling in *tkvKD* somatic cells reduced SCC accumulation. Therefore, we conclude that, during developmental stages and at the adult stage, ovarian somatic cells most likely use different strategies to prevent Dpp leakage from the niche in order to maintain a proper number of GSCs. However, we could not rule out the possibility that Tkv may promote IC or EC proper differentiation, which is required for GSC progeny differentiation. In this case, SCC accumulation in the germaria developed from the somatic *tkvKD* gonad is a consequence of failed differentiation of ICs/or ECs.

### Hh and BMP Signaling in the Soma Are Intertwined in Determining GSC Fate

In this study, we have shown that somatic knockdown of *tkv* in developing ovaries results in formation of ectopic GSCs. In addition, somatic *tkvKD* ovaries exhibit a reduction of *hh* transcript level, which may lead to a reduction of *dpp* transcript levels. These results suggest that Hh signaling is sandwiched by upstream and downstream Dpp signaling in the control of GSC number; as such, Dpp signaling activates transcription of *hh* to promote *dpp* transcription, which in turn activates Dpp signaling in the soma. In adult female flies, both promotion and suppression of Dpp have been reported as effects of Hh signaling in ECs (Huang et al., 2017, Lu et al., 2015, Rojas-Rios et al., 2012). In male flies, Hh is mainly produced in the testes by hub cells in the GSC niche (Zhang et al., 2013). This signal is received by adjacent cyst somatic stem cells, which are also in the GSC niche (Amoyel et al., 2013, Zhang et al., 2013), and further enhances transcription of *dpp*, leading to the activation of Dpp signaling in GSCs to maintain an undifferentiated state. However, the role of Hh signaling in somatic gonadal precursors of the developing testis has not been reported.

In mammals, testicular GSCs directly adhere to the Sertoli cells that constitute the GSC niche (Payne et al., 2010). The Sertoli cells express Desert hedgehog (Dhh), which controls spermatogenesis (Bitgood et al., 1996). During development, Dhh signaling specifies the fetal Leydig cell lineage that produces testosterone for masculinization of a male fetus (Svechnikov et al., 2010, Yao et al., 2002). Despite the known role of Dhh, it is not clear if somatic cells regulate GSCs via BMP and Hh signaling pathways in mammalian testes and ovaries. However, a previous report suggests that cancer stem cells in ovarian cancer may be derived from GSCs (Kim et al., 2014), and cancer-associated mesenchymal stem cells (the niche for cancer stem cells) express a high level of BMP4, which promotes tumor growth by increasing the number of cancer stem cells. The BMP4 signals from cancer-associated mesenchymal stem cells further activate expression of *HH* in cancer stem cells, and that action drives more BMP4 production from the mesenchymal cells, forming a positive feedback loop that confers resistance to chemotherapeutics (Coffman et al., 2016). Together, these studies show that in the niche, complex interactions between BMP and Hh signaling govern GSC and cancer stem cell numbers, and therefore further studies on BMP-Hh regulation may allow us to understand how both GSCs and cancer stem cells are formed, eventually benefiting cancer therapy.

## Experimental Procedures

Fly stocks were maintained at 22°C–25°C on standard medium, unless otherwise indicated. *yw* was used as a wild-type control. *hh-lacZ* and *ptc-lacZ* were used to monitor *hh* transcription and Hh signaling activity (Chen and Struhl, 1996, Forbes et al., 1996). *dpp2.0-lacZ* was used to examine transcriptional activity of *dpp* (Luo et al., 2017). *P2-lacZ* consists of a fragment of the *tkv* promoter (2L 5,237,025–5,245,570 base pairs) containing the first exons of *tkv-B*, -*C*, and –*D* transcripts, followed by a *lacZ* reporter. This construct was used to monitor *tkv* expression (Luo et al., 2015). *UAS-RNAi* lines against *tkv* (N no. 14026-R3 and V no. 3059), *punt* (V no. 107071), *mad* (B no. 31315 and V no. 12635), *hh* (N no. 4637-R2), *smo* (B no. 43134), *dally* (N no. 4974-R1), *dpp* (N no. 9885-R2), *ptc* (N no. 2411R1), and *GFP* (B no. 9331 and B no. 9330) were obtained from National Institute of Genetics (N), Vienna Drosophila Resource Center (V), or Bloomington Drosophila Stock Center (B). The efficiency of the *dpp*^*RNAi*^ line was examined in this study (see Figures S4I and S4J), and the efficiencies of other *RNAi* lines have been reported previously (Figeac et al., 2010, Lai et al., 2017, Lesokhin et al., 1999, Okano et al., 1994). *UASp-mCD8-gfp*, *UAS-hh-GFP*, *UAS-tkv*^*CA*^ (B no. 36537), *UAS-egfr*^*DN*^ (B no. 5364), *UAS-egfr*^*A887T*^ (B no. 9533), and *UAS-egfr*^*λTop 3.1*^ (a gift from Dr. Henry Sun, Academia Sinica, Taiwan) have been described previously (Bolivar et al., 2006, Guo and Wang, 2009, Kao et al., 2015, Lai et al., 2017, Li et al., 2003, Queenan et al., 1997, Torroja et al., 2004, Zhu and Xie, 2003). Flies expressing RNAi or other transgenes driven by *bab1-GAL4* or *c587-GAL4* also carried *tub-GAL80*^*ts*^ to control GAL4 expression; those flies were cultured at 18°C to silence GAL4 expression and were cultured at 29°C to allow GAL4 expression (McGuire et al., 2004). Other genetic tools are described in flybase (http://flybase.org).

Other detail experimental procedures are shown in Supplemental Information.

## Author Contributions

C.-Y.T., H.J.H., and Y.-H.S. conceived and designed the experiments. C.-M.L., C.-Y.T., Y. Cho, S.-M.Y, K.-Y.L., and Y.-H.S. performed the experiments. Y. Cai provided fly lines and reagents, discussed and interpreted results, and troubleshot experiments. C.-Y.T. and H.-J.H. analyzed the data and wrote the manuscript. O.A., K.-Y.L., and E.R. finalized the revised experiments.

## Supplemental Information

Document S1. Supplemental Experimental Procedures and Figures S1–S6

Document S2. Article plus Supplemental Information
