# Supplementary material for: Smad-Independent BMP Signaling in Somatic Cells Limits the Size of the Germline Stem Cell Pool
Source: Stem Cell Reports. 2018 Aug 16;11(3):811–27. doi: 10.1016/j.stemcr.2018.07.008 (PMC6135924; doi:10.1016/j.stemcr.2018.07.008)
Supplement: Document S1. Supplemental Experimental Procedures and Figures S1–S6 [file mmc1.pdf]

Stem Cell Reports, Volume *11*

Supplemental Information

Smad-Independent BMP Signaling in Somatic Cells Limits the Size of the Germline Stem Cell Pool

Chen-Yuan Tseng, Yu-Han Su, Shun-Min Yang, Kun-Yang Lin, Chun-Ming Lai, Elham Rastegari, Oyundari Amartuvshin, Yueh Cho, Yu Cai, and Hwei-Jan Hsu

Supplementary information

Four *tkv* transcripts share identical coding region sequences.

Black squares mark the translational start codon of *tkv* transcripts.

```
              10        20        30        40        50        60        70        80        90       100
      ....|....|....|....|....|....|....|....|....|....|....|....|....|....|....|....|....|....|....|....|
tkv A ---------------------------------------------------------------------------------------------------- 1
tkv B ---------------------------------------------------------------------------------------------------- 1
tkv C ---------------------------------------------------------------------------------------------------- 1
tkv D TTGCACATTCGTCGATACGTTCTTCGCGAGTCGTGACGTATCCACCGGATTGGAAAAATAAACCATATTTTGAGATACAATCCAAAGAATCGTCGATTTT 100

             110       120       130       140       150       160       170       180       190       200
      ....|....|....|....|....|....|....|....|....|....|....|....|....|....|....|....|....|....|....|....|
tkv A ---------------------------------------------------------------------------------------------------- 1
tkv B ---------------------------------------------------------------------------------------------------- 1
tkv C ---------------------------------------------------------------------------------------------------- 1
tkv D GTTTACGCGTGAAATATTTTCGAGTTTCAACCAAAAACCTGTGTGCCTAAGCTGAAATATTGTTAACTAATGAAAAACGAATAATAAAAGTGCATCAACG 200

             210       220       230       240       250       260       270       280       290       300
      ....|....|....|....|....|....|....|....|....|....|....|....|....|....|....|....|....|....|....|....|
tkv A ---------------------------------------------------------------------------------------------------- 1
tkv B ---------------------------------------------------------------------------------------------------- 1
tkv C ---------------------------------------------------------------------------------------------------- 1
tkv D GCAATCTATAGTTAATGATTTAAATGGGAAATTGCACATATATTTCTATGCTTAAACAAGTGCCAGTGCGAATAAACAAAATAGTGTAAACACTGGGGAA 300

             310       320       330       340       350       360       370       380       390       400
      ....|....|....|....|....|....|....|....|....|....|....|....|....|....|....|....|....|....|....|....|
tkv A ---------------------------------------------------------------------------------------------------- 1
tkv B ---------------------------------------------------------------------------------------------------- 1
tkv C ---------------------------------------------------------------------------------------------------- 1
tkv D AAACAACCAGAAACACACAGGATATGCCTTAACTGCGGATTGAGTTTCACCTGATTTGTATTGCCACCTTTCGTATATAATTATAATCAACGTGATCACG 400

             410       420       430       440       450       460       470       480       490       500
      ....|....|....|....|....|....|....|....|....|....|....|....|....|....|....|....|....|....|....|....|
tkv A ---------------------------------------------------------------------------------------------------- 1
tkv B ----------------------------------TGTATAGCGGTTTC-CCATAATTTACACCACTCATGTTACGCTAAAGTTTT-ATTGAGTTCCATTA 64
tkv C ---------------------------------------------------------------------------------------------------- 1
tkv D CGCTCACACCAAGCAAAAGCAAAAGCACATCGGCAGCAGAGCACCAACGCCACCACCGCCAGCAACAGCAGCAGGATCAAGTTTTTGGCGAAACCCATAA 500

             510       520       530       540       550       560       570       580       590       600
      ....|....|....|....|....|....|....|....|....|....|....|....|....|....|....|....|....|....|....|....|
tkv A ---------------------------------------------------------------------------------------------------- 1
tkv B ATTTACGACTTTCTATGGTCGTAATTAAGATTATCCTTTGCTCGCGCTCCGCAATTGCCGCCGATTTTCCATGAATGGCAGCTTTTTATCGGACCT---- 160
tkv C ---------------------------------------------------------------------------------------------------- 1
tkv D TTTCGCTCATCTTCCTGCTCCTCTTCTTCTTCTTTTTCTTCGCGGGCTCCATGGTGTCGCCGGCTTGACCGCCGCCGCCTGCCTATAAACAAACCCAAGA 600

             610       620       630       640       650       660       670       680       690       700
      ....|....|....|....|....|....|....|....|....|....|....|....|....|....|....|....|....|....|....|....|
tkv A ---------------------------------------------------------------------------------------------------- 1
tkv B ---GCACTCGCATAATTCATTTTATCATTTTGACAGATTCGGAA----TGGAATAGCGACTTTCCTCGCCCACCCAACACGCGTACGAGATTTCAATTGC 253
tkv C -----------------------------------------------------------------------ATTTAATGTATGGTTCGTATA--AGGTGC 27
tkv D GGAGTCCAACCCCAACTCCAGCTCCAACTTCGACTGCGACGGCGACTCCATAACAACGCGAAGACTCGACGAGATACAACGAATCCAGTTCTCCTGGTGC 700

             710       720       730       740       750       760       770       780       790       800
      ....|....|....|....|....|....|....|....|....|....|....|....|....|....|....|....|....|....|....|....|
tkv A ----------------------------------------------CAGC------TCT--ATCGGGCACACGCTGTTAAAGTTTATTTGTACTGT---- 42
tkv B -------------------CCGTCGAGTGGCACACATATATTATACCAGC------TCTCGAAATGAGACGCATCATAAATAATAACTAATGTTTTGATA 328
tkv C -------------------CGTCCTAGTGCTTCT----TGCGACCCTGGC------AGAGAGATTGATGCGCCTT-CAGCTGGTGGCCCTTGAACT--CC 95
tkv D TTTTGTTTTGCTTTCGTTGCTGCTGGCTGCCTCGCGTTTATTGTTTTGCTGCATAGCCCGGAAAAAATAAACGTGGCAACGAGGGGATATCGAAGGGGAA 800

             810       820       830       840       850       860       870       880       890       900
      ....|....|....|....|....|....|....|....|....|....|....|....|....|....|....|....|....|....|....|....|
tkv A T--AACGCGAA------TTGGATTAAAATGTTTTGTTTTTTGTTAGTTTTCGGTGTAAATGTGGTTTTAGTGCACTTAACAACTGGTGAATTCAGTGCGA 134
tkv B A--GACCCCAA------TGAGCGCCAACCACTTTTTCTGTTTATCAAATTACGTATGAA-GTTTGCTCATTAATTTTGGCAA--AGTGAATTCAGTGCGA 417
tkv C A--GGCCACA-------TCTGGGACAGCCGACGGTCTGTTTGAAGAGATTGCACTAGAG-G------CGCTGAC----GCGG--AGTGAATTCAGTGCGA 173
tkv D ACTGGCCCCAAAACACCCGGGATCCCGATACCCTCTCTATATAGAGAGGAAGGCCAACAAAAACAACCAAGAACGATCGGTTGGGGCAACAACAACTTAG 900

             910       920       930       940       950       960       970       980       990      1000
      ....|....|....|....|....|....|....|....|....|....|....|....|....|....|....|....|....|....|....|....|
tkv A ATGTTCAGCCAGACGTCGAAGC--TATGGCCGCCGCCG--CTTTGAGTGGCATGGAAATGGGATCTGGACCAGGATCAGAGGGATATGAGGATGCCGACA 230
tkv B ATGTTCAGCCAGACGTCGAAGC--TATGGCCGCCGCCG--CTTTGAGTGGCATGGAAATGGGATCTGGACCAGGATCAGAGGGATATGAGGATGCCGACA 513
tkv C ATGTTCAGCCAGACGTCGAAGC--TATGGCCGCCGCCG--CTTTGAGTGGCATGGAAATGGGATCTGGACCAGGATCAGAGGGATATGAGGATGCCGACA 269
tkv D TTGCATTGC-AGTTGTTGCCGCCGTATAAAAATCATCAGCCTTTGGCCCACGTCGTTGCTGTTGTTGTTGTTGCTCTTGTTGTAGCCATCAGACATGGCG 999

            1010      1020      1030      1040      1050      1060      1070      1080      1090      1100
      ....|....|....|....|....|....|....|....|....|....|....|....|....|....|....|....|....|....|....|....|
tkv A ACGAGAAA-AGCAAGACCGTGGAAAATGCCCGCTCCCTAACCTGCTACTGCGATGGCAGTTGTCCGGACAATGTAAGCAATGGAACCTGCGAGACCAGAC 329
tkv B ACGAGAAA-AGCAAGACCGTGGAAAATGCCCGCTCCCTAACCTGCTACTGCGATGGCAGTTGTCCGGACAATGTAAGCAATGGAACCTGCGAGACCAGAC 612
tkv C ACGAGAAA-AGCAAGACCGTGGAAAATGCCCGCTCCCTAACCTGCTACTGCGATGGCAGTTGTCCGGACAATGTAAGCAATGGAACCTGCGAGACCAGAC 368
tkv D CCGAAATCCAGAAAGAAGAAGGCTCATGCCCGCTCCCTAACCTGCTACTGCGATGGCAGTTGTCCGGACAATGTAAGCAATGGAACCTGCGAGACCAGAC 1099

            1110      1120      1130      1140      1150      1160      1170      1180      1190      1200
```

```
      ....|....|....|....|....|....|....|....|....|....|....|....|....|....|....|....|....|....|....|....|
tkv A  CCGGTGGCAGTTGCTTCAGCGCAGTCCAACAGCTTTACGATGAGACGACCGGGATGTACGAGGAGGAGCGTACATATGGATGCATGCCTCCCGAAGACAA 429
tkv B  CCGGTGGCAGTTGCTTCAGCGCAGTCCAACAGCTTTACGATGAGACGACCGGGATGTACGAGGAGGAGCGTACATATGGATGCATGCCTCCCGAAGACAA 712
tkv C  CCGGTGGCAGTTGCTTCAGCGCAGTCCAACAGCTTTACGATGAGACGACCGGGATGTACGAGGAGGAGCGTACATATGGATGCATGCCTCCCGAAGACAA 468
tkv D  CCGGTGGCAGTTGCTTCAGCGCAGTCCAACAGCTTTACGATGAGACGACCGGGATGTACGAGGAGGAGCGTACATATGGATGCATGCCTCCCGAAGACAA 1199

             1210      1220      1230      1240      1250      1260      1270      1280      1290      1300
      ....|....|....|....|....|....|....|....|....|....|....|....|....|....|....|....|....|....|....|....|
tkv A  CGGTGGTTTTCTCATGTGCAAGGTAGCCGCTGTACCCCACCTGCATGGCAAGAACATTGTCTGCTGCGACAAGGAGGACTTCTGCAACCGTGACCTGTAC 529
tkv B  CGGTGGTTTTCTCATGTGCAAGGTAGCCGCTGTACCCCACCTGCATGGCAAGAACATTGTCTGCTGCGACAAGGAGGACTTCTGCAACCGTGACCTGTAC 812
tkv C  CGGTGGTTTTCTCATGTGCAAGGTAGCCGCTGTACCCCACCTGCATGGCAAGAACATTGTCTGCTGCGACAAGGAGGACTTCTGCAACCGTGACCTGTAC 568
tkv D  CGGTGGTTTTCTCATGTGCAAGGTAGCCGCTGTACCCCACCTGCATGGCAAGAACATTGTCTGCTGCGACAAGGAGGACTTCTGCAACCGTGACCTGTAC 1299

             1310      1320      1330      1340      1350      1360      1370      1380      1390      1400
      ....|....|....|....|....|....|....|....|....|....|....|....|....|....|....|....|....|....|....|....|
tkv A  CCCACCTACACACCCAAGCTGACCACACCAGCGCCGGATTTGCCCGTGAGCAGCGAGTCCCTACACACGCTGGCCGTCTTTGGCTCCATCATCATCTCCC 629
tkv B  CCCACCTACACACCCAAGCTGACCACACCAGCGCCGGATTTGCCCGTGAGCAGCGAGTCCCTACACACGCTGGCCGTCTTTGGCTCCATCATCATCTCCC 912
tkv C  CCCACCTACACACCCAAGCTGACCACACCAGCGCCGGATTTGCCCGTGAGCAGCGAGTCCCTACACACGCTGGCCGTCTTTGGCTCCATCATCATCTCCC 668
tkv D  CCCACCTACACACCCAAGCTGACCACACCAGCGCCGGATTTGCCCGTGAGCAGCGAGTCCCTACACACGCTGGCCGTCTTTGGCTCCATCATCATCTCCC 1399

             1410      1420      1430      1440      1450      1460      1470      1480      1490      1500
      ....|....|....|....|....|....|....|....|....|....|....|....|....|....|....|....|....|....|....|....|
tkv A  TGTCCGTGTTTATGCTGATCGTGGCTAGCTTATGTTTCACCTACAAGCGACGCGAGAAGCTGCGCAAGCAGCCACGTCTCATCAACTCAATGTGCAACTC 729
tkv B  TGTCCGTGTTTATGCTGATCGTGGCTAGCTTATGTTTCACCTACAAGCGACGCGAGAAGCTGCGCAAGCAGCCACGTCTCATCAACTCAATGTGCAACTC 1012
tkv C  TGTCCGTGTTTATGCTGATCGTGGCTAGCTTATGTTTCACCTACAAGCGACGCGAGAAGCTGCGCAAGCAGCCACGTCTCATCAACTCAATGTGCAACTC 768
tkv D  TGTCCGTGTTTATGCTGATCGTGGCTAGCTTATGTTTCACCTACAAGCGACGCGAGAAGCTGCGCAAGCAGCCACGTCTCATCAACTCAATGTGCAACTC 1499

             1510      1520      1530      1540      1550      1560      1570      1580      1590      1600
      ....|....|....|....|....|....|....|....|....|....|....|....|....|....|....|....|....|....|....|....|
tkv A  ACAGCTGTCGCCTTTGTCACAACTGGTGGAACAGAGTTCGGGCTCCGGATCGGGATTACCATTGCTGGTGCAAAGAACCATTGCCAAGCAGATTCAGATG 829
tkv B  ACAGCTGTCGCCTTTGTCACAACTGGTGGAACAGAGTTCGGGCTCCGGATCGGGATTACCATTGCTGGTGCAAAGAACCATTGCCAAGCAGATTCAGATG 1112
tkv C  ACAGCTGTCGCCTTTGTCACAACTGGTGGAACAGAGTTCGGGCTCCGGATCGGGATTACCATTGCTGGTGCAAAGAACCATTGCCAAGCAGATTCAGATG 868
tkv D  ACAGCTGTCGCCTTTGTCACAACTGGTGGAACAGAGTTCGGGCTCCGGATCGGGATTACCATTGCTGGTGCAAAGAACCATTGCCAAGCAGATTCAGATG 1599

             1610      1620      1630      1640      1650      1660      1670      1680      1690      1700
      ....|....|....|....|....|....|....|....|....|....|....|....|....|....|....|....|....|....|....|....|
tkv A  GTGCGACTGGTGGGCAAAGGACGATATGGCGAGGTCTGGCTGGCCAAATGGCGCGATGAGCGGGTGGCCGTCAAGACCTTCTTTACGACCGAAGAGGCTT 929
tkv B  GTGCGACTGGTGGGCAAAGGACGATATGGCGAGGTCTGGCTGGCCAAATGGCGCGATGAGCGGGTGGCCGTCAAGACCTTCTTTACGACCGAAGAGGCTT 1212
tkv C  GTGCGACTGGTGGGCAAAGGACGATATGGCGAGGTCTGGCTGGCCAAATGGCGCGATGAGCGGGTGGCCGTCAAGACCTTCTTTACGACCGAAGAGGCTT 968
tkv D  GTGCGACTGGTGGGCAAAGGACGATATGGCGAGGTCTGGCTGGCCAAATGGCGCGATGAGCGGGTGGCCGTCAAGACCTTCTTTACGACCGAAGAGGCTT 1699

             1710      1720      1730      1740      1750      1760      1770      1780      1790      1800
      ....|....|....|....|....|....|....|....|....|....|....|....|....|....|....|....|....|....|....|....|
tkv A  CTTGGTTCCGCGAGACTGAAATCTATCAGACAGTGCTGATGCGACACGACAATATCTTGGGCTTCATTGCCGCCGATATCAAGGGTAATGGTAGCTGGAC 1029
tkv B  CTTGGTTCCGCGAGACTGAAATCTATCAGACAGTGCTGATGCGACACGACAATATCTTGGGCTTCATTGCCGCCGATATCAAGGGTAATGGTAGCTGGAC 1312
tkv C  CTTGGTTCCGCGAGACTGAAATCTATCAGACAGTGCTGATGCGACACGACAATATCTTGGGCTTCATTGCCGCCGATATCAAGGGTAATGGTAGCTGGAC 1068
tkv D  CTTGGTTCCGCGAGACTGAAATCTATCAGACAGTGCTGATGCGACACGACAATATCTTGGGCTTCATTGCCGCCGATATCAAGGGTAATGGTAGCTGGAC 1799

             1810      1820      1830      1840      1850      1860      1870      1880      1890      1900
      ....|....|....|....|....|....|....|....|....|....|....|....|....|....|....|....|....|....|....|....|
tkv A  ACAGATGTTGCTGATCACCGACTACCACGAGATGGGCAGCCTACACGATTACCTCTCAATGTCGGTGATCAATCCGCAGAAGCTGCAATTGCTGGCGTTT 1129
tkv B  ACAGATGTTGCTGATCACCGACTACCACGAGATGGGCAGCCTACACGATTACCTCTCAATGTCGGTGATCAATCCGCAGAAGCTGCAATTGCTGGCGTTT 1412
tkv C  ACAGATGTTGCTGATCACCGACTACCACGAGATGGGCAGCCTACACGATTACCTCTCAATGTCGGTGATCAATCCGCAGAAGCTGCAATTGCTGGCGTTT 1168
tkv D  ACAGATGTTGCTGATCACCGACTACCACGAGATGGGCAGCCTACACGATTACCTCTCAATGTCGGTGATCAATCCGCAGAAGCTGCAATTGCTGGCGTTT 1899

             1910      1920      1930      1940      1950      1960      1970      1980      1990      2000
      ....|....|....|....|....|....|....|....|....|....|....|....|....|....|....|....|....|....|....|....|
tkv A  TCGCTGGCCTCCGGATTGGCCCACCTGCACGACGAGATTTTCGGAACCCCTGGCAAACCAGCTATCGCTCATCGCGATATCAAGAGCAAGAACATTTTGG 1229
tkv B  TCGCTGGCCTCCGGATTGGCCCACCTGCACGACGAGATTTTCGGAACCCCTGGCAAACCAGCTATCGCTCATCGCGATATCAAGAGCAAGAACATTTTGG 1512
tkv C  TCGCTGGCCTCCGGATTGGCCCACCTGCACGACGAGATTTTCGGAACCCCTGGCAAACCAGCTATCGCTCATCGCGATATCAAGAGCAAGAACATTTTGG 1268
tkv D  TCGCTGGCCTCCGGATTGGCCCACCTGCACGACGAGATTTTCGGAACCCCTGGCAAACCAGCTATCGCTCATCGCGATATCAAGAGCAAGAACATTTTGG 1999

             2010      2020      2030      2040      2050      2060      2070      2080      2090      2100
      ....|....|....|....|....|....|....|....|....|....|....|....|....|....|....|....|....|....|....|....|
tkv A  TCAAGCGGAATGGGCAGTGCGCTATTGCTGACTTCGGGCTGGCAGTGAAGTACAACTCGGAACTGGATGTCATTCACATTGCACAGAATCCACGTGTCGG 1329
tkv B  TCAAGCGGAATGGGCAGTGCGCTATTGCTGACTTCGGGCTGGCAGTGAAGTACAACTCGGAACTGGATGTCATTCACATTGCACAGAATCCACGTGTCGG 1612
tkv C  TCAAGCGGAATGGGCAGTGCGCTATTGCTGACTTCGGGCTGGCAGTGAAGTACAACTCGGAACTGGATGTCATTCACATTGCACAGAATCCACGTGTCGG 1368
tkv D  TCAAGCGGAATGGGCAGTGCGCTATTGCTGACTTCGGGCTGGCAGTGAAGTACAACTCGGAACTGGATGTCATTCACATTGCACAGAATCCACGTGTCGG 2099

             2110      2120      2130      2140      2150      2160      2170      2180      2190      2200
      ....|....|....|....|....|....|....|....|....|....|....|....|....|....|....|....|....|....|....|....|
tkv A  CACTCGACGCTACATGGCTCCAGAAGTATTGAGTCAGCAGCTGGATCCCAAGCAGTTTGAAGAGTTCAAACGGGCTGATATGTATTCAGTGGGTCTCGTT 1429
tkv B  CACTCGACGCTACATGGCTCCAGAAGTATTGAGTCAGCAGCTGGATCCCAAGCAGTTTGAAGAGTTCAAACGGGCTGATATGTATTCAGTGGGTCTCGTT 1712
tkv C  CACTCGACGCTACATGGCTCCAGAAGTATTGAGTCAGCAGCTGGATCCCAAGCAGTTTGAAGAGTTCAAACGGGCTGATATGTATTCAGTGGGTCTCGTT 1468
tkv D  CACTCGACGCTACATGGCTCCAGAAGTATTGAGTCAGCAGCTGGATCCCAAGCAGTTTGAAGAGTTCAAACGGGCTGATATGTATTCAGTGGGTCTCGTT 2199

             2210      2220      2230      2240      2250      2260      2270      2280      2290      2300
      ....|....|....|....|....|....|....|....|....|....|....|....|....|....|....|....|....|....|....|....|
tkv A  CTGTGGGAGATGACCCGTCGCTGCTACACACCCGTATCGGGCACCAAGACGACCACCTGCGAGGACTACGCCCTGCCCTATCACGATGTGGTGCCCTCGG 1529
tkv B  CTGTGGGAGATGACCCGTCGCTGCTACACACCCGTATCGGGCACCAAGACGACCACCTGCGAGGACTACGCCCTGCCCTATCACGATGTGGTGCCCTCGG 1812
tkv C  CTGTGGGAGATGACCCGTCGCTGCTACACACCCGTATCGGGCACCAAGACGACCACCTGCGAGGACTACGCCCTGCCCTATCACGATGTGGTGCCCTCGG 1568
tkv D  CTGTGGGAGATGACCCGTCGCTGCTACACACCCGTATCGGGCACCAAGACGACCACCTGCGAGGACTACGCCCTGCCCTATCACGATGTGGTGCCCTCGG 2299

             2310      2320      2330      2340      2350      2360      2370      2380      2390      2400
      ....|....|....|....|....|....|....|....|....|....|....|....|....|....|....|....|....|....|....|....|
tkv A  ATCCCACGTTCGAGGACATGCACGCTGTTGTGTGCGTAAAGGGTTTCCGGCCGCCGATACCATCACGCTGGCAGGAGGATGATGTACTCGCCACCGTATC 1629
```

```
tkv B  ATCCCACGTTCGAGGACATGCACGCTGTTGTGTGCGTAAAGGGTTTCCGGCCGCCGATACCATCACGCTGGCAGGAGGATGATGTACTCGCCACCGTATC 1912
tkv C  ATCCCACGTTCGAGGACATGCACGCTGTTGTGTGCGTAAAGGGTTTCCGGCCGCCGATACCATCACGCTGGCAGGAGGATGATGTACTCGCCACCGTATC 1668
tkv D  ATCCCACGTTCGAGGACATGCACGCTGTTGTGTGCGTAAAGGGTTTCCGGCCGCCGATACCATCACGCTGGCAGGAGGATGATGTACTCGCCACCGTATC 2399

               2410      2420      2430      2440      2450      2460      2470      2480      2490      2500
       ....|....|....|....|....|....|....|....|....|....|....|....|....|....|....|....|....|....|....|....|
tkv A  CAAGATCATGCAGGAGTGCTGGCACCCGAATCCCACCGTTCGGCTGACTGCCCTGCGCGTAAAGAAGACGCTGGGGCGACTGGAAACAGACTGTCTAATC 1729
tkv B  CAAGATCATGCAGGAGTGCTGGCACCCGAATCCCACCGTTCGGCTGACTGCCCTGCGCGTAAAGAAGACGCTGGGGCGACTGGAAACAGACTGTCTAATC 2012
tkv C  CAAGATCATGCAGGAGTGCTGGCACCCGAATCCCACCGTTCGGCTGACTGCCCTGCGCGTAAAGAAGACGCTGGGGCGACTGGAAACAGACTGTCTAATC 1768
tkv D  CAAGATCATGCAGGAGTGCTGGCACCCGAATCCCACCGTTCGGCTGACTGCCCTGCGCGTAAAGAAGACGCTGGGGCGACTGGAAACAGACTGTCTAATC 2499

               2510      2520      2530      2540      2550      2560      2570      2580      2590      2600
       ....|....|....|....|....|....|....|....|....|....|....|....|....|....|....|....|....|....|....|....|
tkv A  GATGTGCCCATTAAGATTGTCTAAGCAGCTGTTTGTAGTCTCGTTTTAGGTTTAACCTAAGTTGCATTTAGTTTTCTGTTTTTGTTCAACTTCTGTTGAA 1829
tkv B  GATGTGCCCATTAAGATTGTCTAAGCAGCTGTTTGTAGTCTCGTTTTAGGTTTAACCTAAGTTGCATTTAGTTTTCTGTTTTTGTTCAACTTCTGTTGAA 2112
tkv C  GATGTGCCCATTAAGATTGTCTAAGCAGCTGTTTGTAGTCTCGTTTTAGGTTTAACCTAAGTTGCATTTAGTTTTCTGTTTTTGTTCAACTTCTGTTGAA 1868
tkv D  GATGTGCCCATTAAGATTGTCTAAGCAGCTGTTTGTAGTCTCGTTTTAGGTTTAACCTAAGTTGCATTTAGTTTTCTGTTTTTGTTCAACTTCTGTTGAA 2599

               2610      2620      2630      2640      2650      2660      2670      2680      2690      2700
       ....|....|....|....|....|....|....|....|....|....|....|....|....|....|....|....|....|....|....|....|
tkv A  TACGCAGTTCCGACCTTTTGTCCGGAAATTCATAGTTAGGTTCTAATCGTAAGTTGCTTGTACGTGAGCGGAGTAAAGCAGAGGATCCTTCAGAGGTGCC 1929
tkv B  TACGCAGTTCCGACCTTTTGTCCGGAAATTCATAGTTAGGTTCTAATCGTAAGTTGCTTGTACGTGAGCGGAGTAAAGCAGAGGATCCTTCAGAGGTGCC 2212
tkv C  TACGCAGTTCCGACCTTTTGTCCGGAAATTCATAGTTAGGTTCTAATCGTAAGTTGCTTGTACGTGAGCGGAGTAAAGCAGAGGATCCTTCAGAGGTGCC 1968
tkv D  TACGCAGTTCCGACCTTTTGTCCGGAAATTCATAGTTAGGTTCTAATCGTAAGTTGCTTGTACGTGAGCGGAGTAAAGCAGAGGATCCTTCAGAGGTGCC 2699

               2710      2720      2730      2740      2750      2760      2770      2780      2790      2800
       ....|....|....|....|....|....|....|....|....|....|....|....|....|....|....|....|....|....|....|....|
tkv A  TATACTACGGTTACTTAAACCGTCGCACTGATTGTAAGCTAGAGTTTAGATATAAGTTGGCAGGAGCTAGTTACTAAGGCCCCTTGCAAAATCTTTGGTT 2029
tkv B  TATACTACGGTTACTTAAACCGTCGCACTGATTGTAAGCTAGAGTTTAGATATAAGTTGGCAGGAGCTAGTTACTAAGGCCCCTTGCAAAATCTTTGGTT 2312
tkv C  TATACTACGGTTACTTAAACCGTCGCACTGATTGTAAGCTAGAGTTTAGATATAAGTTGGCAGGAGCTAGTTACTAAGGCCCCTTGCAAAATCTTTGGTT 2068
tkv D  TATACTACGGTTACTTAAACCGTCGCACTGATTGTAAGCTAGAGTTTAGATATAAGTTGGCAGGAGCTAGTTACTAAGGCCCCTTGCAAAATCTTTGGTT 2799

               2810      2820      2830      2840      2850      2860      2870      2880      2890      2900
       ....|....|....|....|....|....|....|....|....|....|....|....|....|....|....|....|....|....|....|....|
tkv A  AGAAAAATGCTTTAACTATTTGAGAAATATAGGGAGTATTTTGGATGACACCTATTCATGTCGATGTACATAAATATGAGATCAAAACAAAAAATCAATC 2129
tkv B  AGAAAAATGCTTTAACTATTTGAGAAATATAGGGAGTATTTTGGATGACACCTATTCATGTCGATGTACATAAATATGAGATCAAAACAAAAAATCAATC 2412
tkv C  AGAAAAATGCTTTAACTATTTGAGAAATATAGGGAGTATTTTGGATGACACCTATTCATGTCGATGTACATAAATATGAGATCAAAACAAAAAATCAATC 2168
tkv D  AGAAAAATGCTTTAACTATTTGAGAAATATAGGGAGTATTTTGGATGACACCTATTCATGTCGATGTACATAAATATGAGATCAAAACAAAAAATCAATC 2899

               2910      2920      2930      2940      2950      2960      2970      2980      2990      3000
       ....|....|....|....|....|....|....|....|....|....|....|....|....|....|....|....|....|....|....|....|
tkv A  GAAAGAATCGAAATTTATATGTAACATACATTAATTTTTGTGCCAGCTTTTGTACGCTTTAACTAAACGAGTTAAGTCGCCCATTCTTTGACCCCCGAGG 2229
tkv B  GAAAGAATCGAAATTTATATGTAACATACATTAATTTTTGTGCCAGCTTTTGTACGCTTTAACTAAACGAGTTAAGTCGCCCATTCTTTGACCCCCGAGG 2512
tkv C  GAAAGAATCGAAATTTATATGTAACATACATTAATTTTTGTGCCAGCTTTTGTACGCTTTAACTAAACGAGTTAAGTCGCCCATTCTTTGACCCCCGAGG 2268
tkv D  GAAAGAATCGAAATTTATATGTAACATACATTAATTTTTGTGCCAGCTTTTGTACGCTTTAACTAAACGAGTTAAGTCGCCCATTCTTTGACCCCCGAGG 2999

               3010      3020      3030      3040      3050      3060      3070      3080      3090      3100
       ....|....|....|....|....|....|....|....|....|....|....|....|....|....|....|....|....|....|....|....|
tkv A  CTTTAGCCTCAACATAGAACATGTCCTTATATGTTTTGTTTGTATCTAGTGTTTAAGTGTAAACTTTGCAGACTTTGTGCAAGTAAATGCTGCCTTTTGT 2329
tkv B  CTTTAGCCTCAACATAGAACATGTCCTTATATGTTTTGTTTGTATCTAGTGTTTAAGTGTAAACTTTGCAGACTTTGTGCAAGTAAATGCTGCCTTTTGT 2612
tkv C  CTTTAGCCTCAACATAGAACATGTCCTTATATGTTTTGTTTGTATCTAGTGTTTAAGTGTAAACTTTGCAGACTTTGTGCAAGTAAATGCTGCCTTTTGT 2368
tkv D  CTTTAGCCTCAACATAGAACATGTCCTTATATGTTTTGTTTGTATCTAGTGTTTAAGTGTAAACTTTGCAGACTTTGTGCAAGTAAATGCTGCCTTTTGT 3099

               3110      3120      3130      3140      3150      3160      3170      3180      3190      3200
       ....|....|....|....|....|....|....|....|....|....|....|....|....|....|....|....|....|....|....|....|
tkv A  AAAATCCACGGAATTAGTTGCCTTAAGACAAAGTGTAAAGACAGGAGCCAACAGACAGGAGCCCGACTCCCTTCCTCCCCTTTTAGTAGCACATATTTTT 2429
tkv B  AAAATCCACGGAATTAGTTGCCTTAAGACAAAGTGTAAAGACAGGAGCCAACAGACAGGAGCCCGACTCCCTTCCTCCCCTTTTAGTAGCACATATTTTT 2712
tkv C  AAAATCCACGGAATTAGTTGCCTTAAGACAAAGTGTAAAGACAGGAGCCAACAGACAGGAGCCCGACTCCCTTCCTCCCCTTTTAGTAGCACATATTTTT 2468
tkv D  AAAATCCACGGAATTAGTTGCCTTAAGACAAAGTGTAAAGACAGGAGCCAACAGACAGGAGCCCGACTCCCTTCCTCCCCTTTTAGTAGCACATATTTTT 3199

               3210      3220      3230      3240      3250      3260      3270      3280      3290      3300
       ....|....|....|....|....|....|....|....|....|....|....|....|....|....|....|....|....|....|....|....|
tkv A  GTATTTATAACCAGAGATATATAGAACTGGCAGTTGCGTATCTCGAGAAGTGCACTTGTGTCGGCTCTTTTGTTTTGTGTAAATAGCAAAGATGTTTATA 2529
tkv B  GTATTTATAACCAGAGATATATAGAACTGGCAGTTGCGTATCTCGAGAAGTGCACTTGTGTCGGCTCTTTTGTTTTGTGTAAATAGCAAAGATGTTTATA 2812
tkv C  GTATTTATAACCAGAGATATATAGAACTGGCAGTTGCGTATCTCGAGAAGTGCACTTGTGTCGGCTCTTTTGTTTTGTGTAAATAGCAAAGATGTTTATA 2568
tkv D  GTATTTATAACCAGAGATATATAGAACTGGCAGTTGCGTATCTCGAGAAGTGCACTTGTGTCGGCTCTTTTGTTTTGTGTAAATAGCAAAGATGTTTATA 3299

               3310      3320      3330      3340      3350      3360      3370      3380      3390      3400
       ....|....|....|....|....|....|....|....|....|....|....|....|....|....|....|....|....|....|....|....|
tkv A  GGCCGCTAACCATACATATATATAGTATATATATTATATATTATCGTTATAAGCGATTCTGTAAATACTATATATGTATCTTTATCTGTAACCGTTTAAC 2629
tkv B  GGCCGCTAACCATACATATATATAGTATATATATTATATATTATCGTTATAAGCGATTCTGTAAATACTATATATGTATCTTTATCTGTAACCGTTTAAC 2912
tkv C  GGCCGCTAACCATACATATATATAGTATATATATTATATATTATCGTTATAAGCGATTCTGTAAATACTATATATGTATCTTTATCTGTAACCGTTTAAC 2668
tkv D  GGCCGCTAACCATACATATATATAGTATATATATTATATATTATCGTTATAAGCGATTCTGTAAATACTATATATGTATCTTTATCTGTAACCGTTTAAC 3399

               3410      3420      3430      3440      3450      3460      3470      3480      3490      3500
       ....|....|....|....|....|....|....|....|....|....|....|....|....|....|....|....|....|....|....|....|
tkv A  TTATATCTTATCGTTCTTATCATGTAACCTTGTTTAAGCTAGAGTTAGATGACTAGCCTGTAAGAGCAACAAGTAAACTATATGAACTAGATGGATATGT 2729
tkv B  TTATATCTTATCGTTCTTATCATGTAACCTTGTTTAAGCTAGAGTTAGATGACTAGCCTGTAAGAGCAACAAGTAAACTATATGAACTAGATGGATATGT 3012
tkv C  TTATATCTTATCGTTCTTATCATGTAACCTTGTTTAAGCTAGAGTTAGATGACTAGCCTGTAAGAGCAACAAGTAAACTATATGAACTAGATGGATATGT 2768
tkv D  TTATATCTTATCGTTCTTATCATGTAACCTTGTTTAAGCTAGAGTTAGATGACTAGCCTGTAAGAGCAACAAGTAAACTATATGAACTAGATGGATATGT 3499

               3510      3520      3530      3540      3550      3560      3570      3580      3590      3600
       ....|....|....|....|....|....|....|....|....|....|....|....|....|....|....|....|....|....|....|....|
tkv A  ATCTTTCAGTTTTTTTTCTGTTTCATTATTACTGCTCGCAGACCACAAGAACTAAAGTTTTGTATTAAAGCGGTTAGTTAATTTATTTAAAAATAATAAA 2829
tkv B  ATCTTTCAGTTTTTTTTCTGTTTCATTATTACTGCTCGCAGACCACAAGAACTAAAGTTTTGTATTAAAGCGGTTAGTTAATTTATTTAAAAATAATAAA 3112
tkv C  ATCTTTCAGTTTTTTTTCTGTTTCATTATTACTGCTCGCAGACCACAAGAACTAAAGTTTTGTATTAAAGCGGTTAGTTAATTTATTTAAAAATAATAAA 2868
```

```
tkv D  ATCTTTCAGTTTTTTTTCTGTTTCATTATTACTGCTCGCAGACCACAAGAACTAAAGTTTTGTATTAAAGCGGTTAGTTAATTTATTTAAAAATAATAAA 3599

             3610
      ....|....|....|..
tkv A  AAATTATAATTAAACCC 2846
tkv B  AAATTATAATTAAACCC 3129
tkv C  AAATTATAATTAAACCC 2885
tkv D  AAATTATAATTAAACCC 3616
```

Experimental Procedures

Developmental staging of larvae and pupae

The developmental stages of *Drosophila* were morphologically defined as previously described (Ashburner, 2005). Flies were transferred in a fresh vial to lay eggs at 25°C for 6 h and were then removed. The vial was kept at 25°C, and newly hatched larvae were collected as first-instar larvae [~20 h after egg laying (AEL)]. Larvae climbing up and down from food were considered mid-third instar larvae (ML3, ~96 h AEL); at this stage, the GSC niche is forming and recruiting GSCs (Song et al., 2007). Larvae that moved out of food, but for which pupation had not started were considered late-L3 wandering larvae (LL3, ~108 h AEL). When wandering larvae stopped moving, they turning to white pupae were considered to be early pupae (~120 h AEL).

For functional studies of Tkv in the ovarian soma, *tkv* was first knocked down throughout development. To further dissect the developmental timing at which functional Tkv is required in the soma, *tkv* was knocked down either before ML3, after ML3, or after early pupal stage. These stages were easily identified according to the above criteria. Phenotypes were examined in adult germaria of newly eclosed flies in order to avoid any further effects that may occur in adults.

Western blot

Immunobloting was performed as previously described (Tseng et al., 2014). Thirty pairs of LL3 female gonads were dissected and lysed in RIPA buffer (20 mM Tris-HCl pH 7.5, 150 mM NaCl, 1 mM EGTA, 1% NP-40) supplemented with 2X Complete Proteinase Inhibitor Cocktail (Roche) on ice for 1 h. Protein lysates were collected from the supernatant after centrifugation at 4°C; for each sample, 25 μg of lysate was used for analysis. Rabbit anti-phospho-ERK1/2 (Thr202/Thr204) (Cell Signaling Technology, Cat#4370, 1:1000) and rabbit anti-α-tubulin (Sigma T9026, 1:5000) were used as primary antibodies; horseradish peroxidase (HRP)-conjugated goat anti-rabbit IgG (Jackson ImmunoResearch, 1:10,000) was used as secondary antibody. Signals were detected by chemiluminescence with a Western LightningTM Plus-ECL kit (PerkinElmer).

RNA extraction and Quantitative real-time PCR

Total RNA was extracted from 20 pairs of one-day-old ovaries with the GENEzol™ TriRNA Pure kit (Geneaid), according to the manufacturer's manual. Total RNA (1 μg) was reverse transcribed with the Transcriptor First Strand cDNA Synthesis kit (Roche). Steady-state mRNA levels were determined using the LightCycler 480 Probes Master combined with a Universal ProbeLibrary (Roche).

The primer set and probe for each gene were designed from the Roche Universal ProbeLibrary assay design center (https://lifescience.roche.com/global_en.message.html#reference-gene-assays) and are listed below. *tkv*: probe: # 121, 5′-GCCGAAATCCAGAAAGAAGA-3′, 5′-GCAGGTTCCATTGCTTACATT-3′ *dpp*: probe #77, 5′-GTGCGAAGTTTTACACACAAAGA-3′ and 5′-CGCCTTCAGCTTCTCGTC-3′
gbb: probe #59, 5′-CGCTGGAACTCTCGAAATAAA-3′ and 5′-CCACTTGCGATAGCTTCAGA-3′
dally: probe #78, 5′-AGTGGGACTTACAGCGAA
AAAG-3′ and 5′-AGCAGTTGCTCGAACATTCC-3′

RpL19: probe #128, 5′-GAGCGTATTGCCACCAGGA-3′ and 5′- CGATCTCGTCCTCCTTAGCA-3′;

RpL32: probe #117, 5′-CGGATCGATATGCTAAGCTGT-3′ and 5′- CGACGCACTCTGTTGTCG-3′.

RNA sequencing analysis

Forty pairs of ovaries were collected and dissected from one-day-old female flies that were cultured at 29°C throughout all developmental stages. The genotypes of the flies were *c587-GAL4/+; UAS-tkv$^{RNAi(NIG)}$/+*, *UAS-tkv$^{RNAi(NIG)}$/+; bab1-Gal4/+*, or *UAS-tkv$^{RNAi(NIG)}$/+*. Two biological replicate were used in this analysis. Total RNA was extracted by Trizol reagent (Invitrogen) according to the manufacturer's instructions. RNA was quantified by absorbance at 260 nm using a ND-1000 spectrophotometer (Nanodrop Techonology) and quality was assessed using a Bioanalyzer 2100 (Agilent Technology) with a RNA 6000 labchip kit (Agilent Technology). All RNA-seq procedures were carried out according to the manufacturer's protocols from Illumina. Library construction for all samples was accomplished with Agilent's SureSelect Strand Specific RNA library Preparation Kit for 75SE (Paired-End) sequencing on a Solexa platform. The sequence was directly determined using sequencing-by-synthesis technology with a TruSeq SBS kit. Raw sequences were obtained from the Illumina Pipeline software bcl2fastq v2.0 and expected to generate 12.5M (million reads) per sample. The sequences were then filtered to obtain qualified reads. Trimmomatic software was implemented to trim or remove the reads according to the quality score. The gene expression level was calculated as FPKM (Fragment Per Kilobase of transcript per Million mapped reads). For differential expression analysis, CummeRbund was used to perform statistical analysis of gene expression profiles. The reference gene annotations were retrieved from Flybase. Data was deposited in the NCBI GEO under the accession number GSE117251.

Immunohistochemistry and fluorescence microscopy

For immunostaining, gonads and ovaries were dissected, fixed and immunostained at designated stages as described previously (Lai et al., 2017; Tseng et al., 2014). In brief, ovaries were dissected in Grace's insect medium (Lonza) and fixed with 5.3% paraformaldehyde/Grace's insect medium for 13 min with gentle agitation at room temperature. Ovaries were washed in PBST (0.1% Triton X-100 in PBS) 20 min three times, and teased apart in PBST, after which the tissue was incubated with blocking solution (GOAL Bio) for 3 h at room temperature or 4°C overnight. Ovaries were incubated with primary antibodies (diluted in blocking solution) for 3 h at room temperature or 4°C overnight, followed by three or four PBST washes for 30 min each. Then ovaries were incubated with secondary antibodies (diluted in blocking solution) for 3 h at room temperature or 4°C overnight, followed by three to four washes with PBST for 30 min each. The primary antibodies were as follows: mouse anti-Hts (1B1) (Drosophila Studies Hybridoma bank, DSHB, 7H9, 1:50), mouse anti-Lamin (Lam) C (DSHB LC28.26, 1:50), guinea pig anti-Traffic Jam (1:5000; a gift from Dr. Dorothea Godt, University of Toronto, Canada), rabbit anti-Fax (gift from Dr. Eric C. Liebl, 1:1500), mouse anti-β-gal (Promega #Z3783, 1:500), rabbit anti-Vasa (Santa Cruz Sc-30210, 1:500), rabbit anti-GFP (Torry Pines GTX113617, 1:1000), rabbit anti-phospho (p)-ERK (Cell Signaling #4370, 1:200), and rabbit anti-pMad (phospho S423+S425) (Abcam #52903, 1:500). Alexa Fluor 488-, 568- or 633-conjugated goat species-specific secondary antibodies (Molecular Probes, 1:1000) were subsequently used. Samples were stained with 0.5 μg/ml DAPI (Sigma), mounted in 80% glycerol containing 20 μg/mL N-propyl gallate (Sigma), and analyzed with a Zeiss LSM 700 confocal microscope.

GSCs were identified as those cells with a fusome, which was labeled with 1B1 and was juxtaposed to the direct interface between the GSC and the cap cell, which was labeled by LamC (Tseng et al., 2014). Germ cells with round-shaped fusomes and without direct cap cell contacts were counted as SCCs. For measuring ptc-lacZ and hh-lacZ expression, Image J was used to calculate the average fluorescence intensity (arbitrary units) in confocal Z-sections at the largest EC or cap cell nuclear diameter. Statistical analysis was performed using Student's t-test.

***In situ* hybridization**

Fluorescent RNA *in situ* was performed as previously reported (Luo et al., 2015), with slight modifications. In brief, ovaries were dissected in Graces insect medium (Lonza) and fixed with 4% formaldehyde (FA) in PBS-DEPC with 1% DMSO at 4°C overnight. On the second day, ovaries were washed with PBST (0.1% Tween 20 in PBS) for 5 min four times, then dehydrated through a series of ethanol solutions (25%, 50%, 75% and 100%) diluted in PBS for 5 mins in each solution. The dehydrated ovaries were stored at -20°C for at least one overnight period. Ovaries were then rehydrated through a series of ethanol solutions, and treated with proteinase K (50 μg/ml in PBST; Sigma-Aldrich) for 5 min at room temperature. After inactivation of proteinase K by 0.2 M glycine, ovaries were refixed in 4% PFA for 30 min, followed by prehybridization in hybridization solution (50% formamide, 5x SSC, 0.1% Tween-20, 50 μg/μl heparin, 100 μg/ml yeast t-RNA and 10 μg/ml salmon sperm DNA) for 1 h at 60°C. Ovaries were then hybridized with Dig-labeled probes overnight at 60°C. Hybridized tissues were washed with 50% and 25% hybridization solution diluted in 2x SSC, each for 5 mins at 65°C. Then the tissues were rinsed with PBST for 10 min two times, incubated with 3% H_2O_2 in PBST to inactivate endogenous perioxidase (POD), and blocked with 2X blocking buffer in maleic acid (Roche) 1 h at room temperature. Ovaries were incubated with anti-Dig-POD (1: 500, Roche # 11207733910) in blocking buffer at 4°C overnight. After washing with PBST for 10 min six times, signals were developed using a TSA plus fluorescence kit (Perkin Elmer). After washing, ovaries were blocked with blocking solution (GOAL Bio) and then further processed using the immunostaining procedure described above. In order to produce *dally* RNA probes, the 3′ UTR region (~758 bp) of *dally* transcripts was amplified from adult fly ovary cDNA (described above) using a primer set that consisted of 5′-CCAGCCCAAGGGATTGAACA-3′ and 5′-GCATTTCGCATTTCGATTACCC-3′. This fragment was subcloned into a pGEM-T Easy vector (Promega) with two different orientations. Sense and antisense RNA probes labeled with digoxigenin-UTP (Roche) were synthesized from 1 μg of PGEM-T-*dally* plasmids digested with NcoI using the ampliCap^{TM} SP6 high yield message marker kit (Cell Script).

Supplementary Figure legends

Supplementary Fig. 1. Expression of *bab1*-, *c587*- and *hh-Gal4* in the larval gonad and adult germarium. Related to Fig. 1. (A-H) *bab1> gfp* (A, C, E and G) and *c587> gfp* ovaries (B, D, F and H) of late-L3 (LL3) (A and B), 2 hours after pupation (APF) **(**C and D), 48 APF (E and F), and 96 APF (G and H) with signals from GFP (green), Tj (red, ICs), Vasa (blue, germ cells) and LamC in C, G and H (red, nuclear envelopes of TF, cap and stalk cells). **(I and J)** One-day (D)-old *bab1>mCD8-gfp* (I) and c*587> mCD8-gfp* germaria (J) with signals from GFP (green), LamC (red) and 1B1 (red, fusomes). **(K and L)** The mid-L3 (ML3) ovary (K) and one-day-old *hh>mCD8-gfp* germarium (L) with GFP (green), DAPI (blue, DAPI), LamC in L (red), and 1B1 in L (red, fusomes) and Tj in L (gray, escort cell, EC). I', J' and L' only show the mCD8-GFP channel. Scale bar in A-F, 20μm. Scale bar in H-J, 10μm.

Supplementary Fig. 2. Knockdown of somatic Tkv does not affect th number of GSCs, niche cap cells, and egg chamber morphology. Related to Fig. 1. (A and B) Number of cap cells (CpCs) **(A)** and GSCs **(B)** in one-day-old *bab1-GAL4* control (ctrl), *UAS* control, *bab-1>tkv^{RNAi}* $^{(N)}$, and *c587>tkv^{RNAi}* $^{(N)}$ germaria. RNAi was expressed throughout developmental stages at 29°C. The number of analyzed germaria is shown above each bar. The genotype of *bab1-GAL4* control is *bab1-GAL4/+*, and *UAS*

control is *UAS-tkv*$^{RNAi\ (N)}$/+. **(C and D)** One-week-old control (ctrl) (C) and *bab1>tkv*^{RNAi} (D) ovarioles were labeled with LamC (gray, TF and cap cell nuclear envelopes) and 1B1 (gray, fusomes and follicle cell membrane). The control ovariole typically contains a string of five to seven progressively older egg chambers, with the youngest cysts in the apical germarial region and more mature chambers near the oviduct. Knockdown of *tkv* causes spectrosome-containing cell accumulation in the germarium, and fewer egg chambers with normal morphology. These *tkvKD* egg chambers were directly followed by one or two nearly mature eggs, implying that these egg chambers and eggs represent escaped germ cells that have undergone differentiation. However, it is not clear why *bab1>tkv*$^{RNAi\ (N)}$ eggs are not laid. One possibility is that *bab1-GAL4* is also is also expressed in the fat body, which controls vitellogenesis (Yan and Postlethwait, 1990), or the ovarian muscle, which controls ovary contraction for egg laying (Ritsick et al., 2007). *RNAi* was expressed throughout developmental stages at 29°C. The genotype in A is *bab1-GAL4*/+; scale bar, 50 μm.

Supplementary Fig. 3. *dpp* transcripts are not increased in *tkvKD* ovaries, *dpp*^{RNAi} efficiency, and somatic Dpp and Tkv are in the same pathway to limit Bmp signals in the niche. Related to Fig. 3. (A) RNA-seq-based gene expression values (FPKM) and **(B)** qRT-PCR analysis yeild fold changes of *dpp* mRNA in one-day (D)-old control (ctrl) and *c587>tkv*^{RNAi} germaria. Statistics analysis in A is from two biological replicate, and the error bar in B denotes standard error from three independent experiments. **(C and D)** Late-L3 (LL3) *c587>gfp*^{RNAi} and *c587>tkv*$^{RNAi\ (N)}$ gonads with *dpp 2.0-lacZ* (green), Tj (red, ICs) and DAPI (blue, DNA). Dashed lines mark terminal filaments. C' and D' only show *dpp 2.0-lacZ* (gray) channel. **(E and F)** One-day-old *c587>gfp*^{RNAi} and *c587>tkv*$^{RNAi\ (N)}$ germaria with *dpp 2.0-lacZ* (gray), Tj (blue, ICs), 1B1 (red, fusomes) and LamC (red, TF and cap cell nuclear envelopes). Inserts show the indicated region of germaria with only the *dpp 2.0-lacZ* (gray) channel. **(G and H)** *dpp 2.0-lacZ* expression in cap cells of one-day-old *bab-1>gfp*^{RNAi} and *bab-1>tkv*$^{RNAi\ (N)}$ germaria. G' and H' show only the *dpp 2.0-lacZ* channel. **(I and J)** One-day-old control (ctrl) (I) and *bab1>dpp*^{RNAi} germaria (J) with staining for phospho (p)-Mad (gray, Dpp signaling), 1B1 (red, fusomes), LamC (red, TF and cap cell nuclear envelopes) and DAPI (blue, DNA). **(K-M)** One-day-old control (K), *bab1>gfp*^{RNAi} *tkv*^{RNAi} (L) and *bab1>dpp*^{RNAi} *tkv*^{RNAi} germaria (M) with p-Mad (green), 1B1 (red), LamC (red), and *bam-gfp* (blue) labeling. RNAi was expressed from late-L3 to adult (I and J) or throughout developmental stages (K-M) at 25°C. Scale bars, 10 μm. The genotype of the control in A and B is *UAS-tkv*$^{RNAi\ (N)}$/+, in I is *bab1-GAL4/+*, and in K is *bab1-GAL4bam-gfp/+*. ***P < 0.001. Noted that SCC accumulation was milder when the *bab1-GAL4* driver bears a copy of *bam-gfp*, which carries the *bam* coding region fused with GFP under the control of the *bam* promoter (Chen and McKearin, 2003). Perhaps this extra copy of *bam* can lead to some minor compensatory effect.

Supplementary Fig. 4. Knockdown of somatic Tkv slightly increases PGC number, but only *c587>tkv*^{RNAi} gonads exhibited decreased pERK expression. Related to Fig. 4. (A-D) Mid-L3 (ML3) control (A and C), *bab-1>tkv*$^{RNAi\ (N)}$ (B) and *c587>tkv*$^{RNAi\ (N)}$ larval ovaries (D) with 1B1 (green, fusomes) and DAPI (blue, DNA). RNAi was expressed throughout developmental stages at 29°C. **(E)** Average number of PGCs per ovary (gonad) of the indicated genotype. The number of analyzed ovaries is shown above each bar. Error bar indicates standard error; *P < 0.05, ***P < 0.001. The genotype of *bab1-GAL4* control is *bab1-GAL4/+*, and *c587-GAL4* control is *c587-GAL4/+*. **(F and G)** Mid-L3 (ML3) control (ctrl) (F) and *bab1>tkv*$^{RNAi\ (N)}$ gonads (G) with 1B1 (green, fusomes), Tj (red, ICs) and phospho (p)-ERK (gray) labeling. Dashed line indicates terminal filaments. F' and G' only show the pERK channel. Plane A is the same data as shown in the main text, Fig. 4A. Dashed line represent the area used for measuring average (avg.) of pERK signals. **(H)** Average intensity of pERK expression in the region of the control, *c587>tkv*$^{RNAi\ (N)}$ and *bab1>tkv*$^{RNAi\ (N)}$ ML3 gonads containing PGCs and ICs. Number of analyzed gonads are shown above each bar. Error bars, S.D. **, P<0.01. The genotype of the control in A and C is *c587-GAL4/+*.

Supplementary Fig. 5. Forcing Tkv-mediated signaling in the soma activates Egfr signaling in ECs and causes GSC loss. Related to Fig. 4. (A-C) One-day (D)-old control (ctrl), and germaria expressing a constitutively active form of *tkv*, *tkv*^{AC}, driven by *bab1-GAL4* (B) and *c587-GAL4* (C) from mid-L3 to D1. Germaria are labeled with 1B1 (red, fusomes), LamC (red, TF and cap cell nuclear envelopes), and phospho (p)-ERK (gray). A'-C' only show the pERK channel; the asterisks in C and C' indicate a germ cell cyst. **(D-G)** One-day-old control (D), *bab1>tkv*^{CA} (whole stage overexpression) (E), and *c587>tkv*^{CA} germaria (whole stage overexpression) (F and G) with 1B1 (gray) and LamC (gray) labeling. **(H and I)** Number of cap cells (CpCs) (H) and GSCs (I) in one-day-old, *UAS* control, *bab-1>tkv*^{CA}, and *c587>tkv*^{CA} germaria. The number of analyzed germaria is shown above each bar. The genotype of *UAS* control is *UAS-tkv*^{CA}/+. Scale bars, 10 μm. ***P < 0.001.

Supplementary Fig. 6. Hh signaling in the developing ovarian soma, not regulated by Med, promotes PGC differentiation, Tkv controls membrane extension of ECs. Related to Fig. 5. (A and B) Knockdown mad in ovarian somatic cells during development does not affect Hh signaling in adult ECs. One-day (D)-old c587>*gfp*^{RNAi} (A) and *c587>mad*$^{RNAi\ (B)}$ (B) with *ptc-lacZ* (gray, Hh signaling reporter) and DAPI. *RNAi* was expressed from L3 to Day1 at 29 °C. Asterisks mark sheath cells. Scale bar is 10 μm. **(C-K)** Knockdown Hh signaling in the developing ovarian soma results in spectrosom-containing cell (SCC) accumulation. One-day (D)-old control (ctrl) (C and I), *en>hh*^{RNAi} (D), *hh>hh*^{RNAi} (E), *bab1>hh*^{RNAi} (F), *c587>hh*^{RNAi} (G), *bab1& c587>hh*^{RNAi} (H), *bab1>smo*^{RNAi} (J) and *c587>smo*^{RNAi} germaria (K) with staining for 1B1 (green, fusomes), LamC (green, terminal filament and cap cell nuclear envelopes), and Tj (in D-I, magenta, EC nuclei), and DAPI (blue, DNA). *RNAi* was expressed from Late-L3 (LL3) to Day1 at 29 °C. Scale bar is 10 μm. The genotype of C is *UAS-hh*^{RNAi}/+, and of I is *UAS-*

smo^{RNAi}/+. **(L)** Average number of SCC in control, *bab1>hh*^{RNAi}, *c587>hh*^{RNAi} and *bab1& c587>hh*^{RNA} geramaria. Error bars indicate standard error; ****P* < 0.001. **(M-O)** Knockdown *tkv* in the developing ovarian soma disrupts EC membrane extension. One-day-old control (M), *bab1>tkv*$^{RNAi\ (N)}$ (N) and *c587>tkv*$^{RNAi\ (N)}$ (O) with 1B1 (red, fusomes), LamC (red, terminal filament and cap cell nuclear envelopes), and Fax (green, EC). Scale bar, 10 μm. Arrows in M indicate membrane extension of ECs. M'-N' only show the Fax channel. The genotype of M is *UAS-tkv*^{RNAi}/+.

References:

Ashburner, M., (2005). Drosophila: A laboratory Handbook. . Cold Spring Harbor Laboratory Press, Cold Spring Harbor, NY.

Chen, D., McKearin, D.M., (2003). A discrete transcriptional silencer in the bam gene determines asymmetric division of the Drosophila germline stem cell. Development 130, 1159.

Lai, C.M., Lin, K.Y., Kao, S.H., Chen, Y.N., Huang, F., Hsu, H.J., (2017). Hedgehog signaling establishes precursors for germline stem cell niches by regulating cell adhesion. J Cell Biol 216, 1439-1453.

Luo, L., Wang, H., Fan, C., Liu, S., Cai, Y., (2015). Wnt ligands regulate Tkv expression to constrain Dpp activity in the Drosophila ovarian stem cell niche. J Cell Biol 209, 595-608.

Ritsick, D.R., Edens, W.A., Finnerty, V., Lambeth, J.D., (2007). Nox regulation of smooth muscle contraction. Free Radical Biology and Medicine 43, 31-38.

Song, X., Call, G.B., Kirilly, D., Xie, T., (2007). Notch signaling controls germline stem cell niche formation in the Drosophila ovary. Development 134, 1071-1080.

Tseng, C.Y., Kao, S.H., Wan, C.L., Cho, Y., Tung, S.Y., Hsu, H.J., (2014). Notch signaling mediates the age-associated decrease in adhesion of germline stem cells to the niche. PLoS Genet 10, e1004888.

Yan, Y.L., Postlethwait, J.H., (1990). Vitellogenesis in Drosophila: sequestration of a yolk polypeptide/invertase fusion protein into developing oocytes. Dev Biol 140, 281-290.

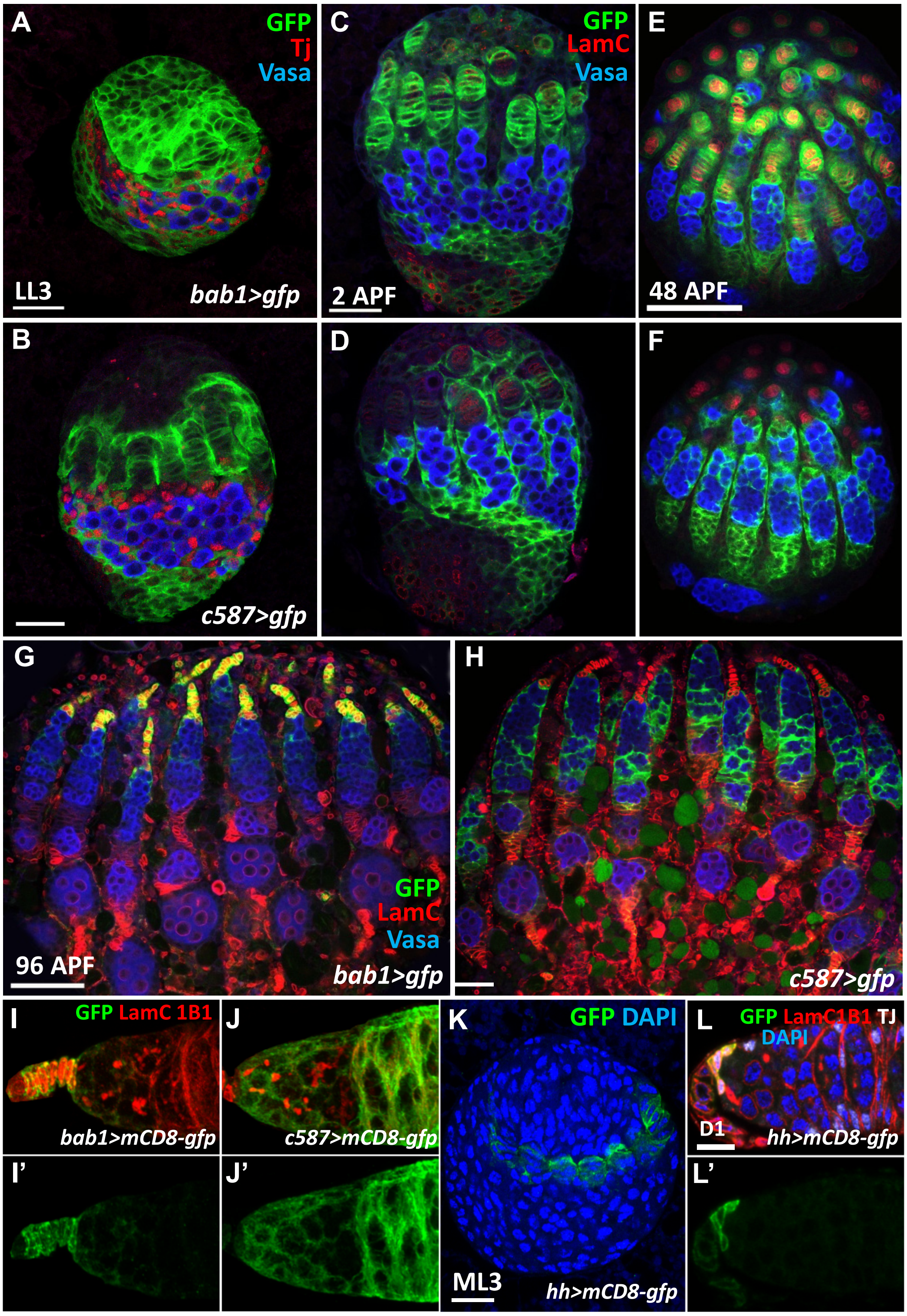

Supplementary Figure. 1

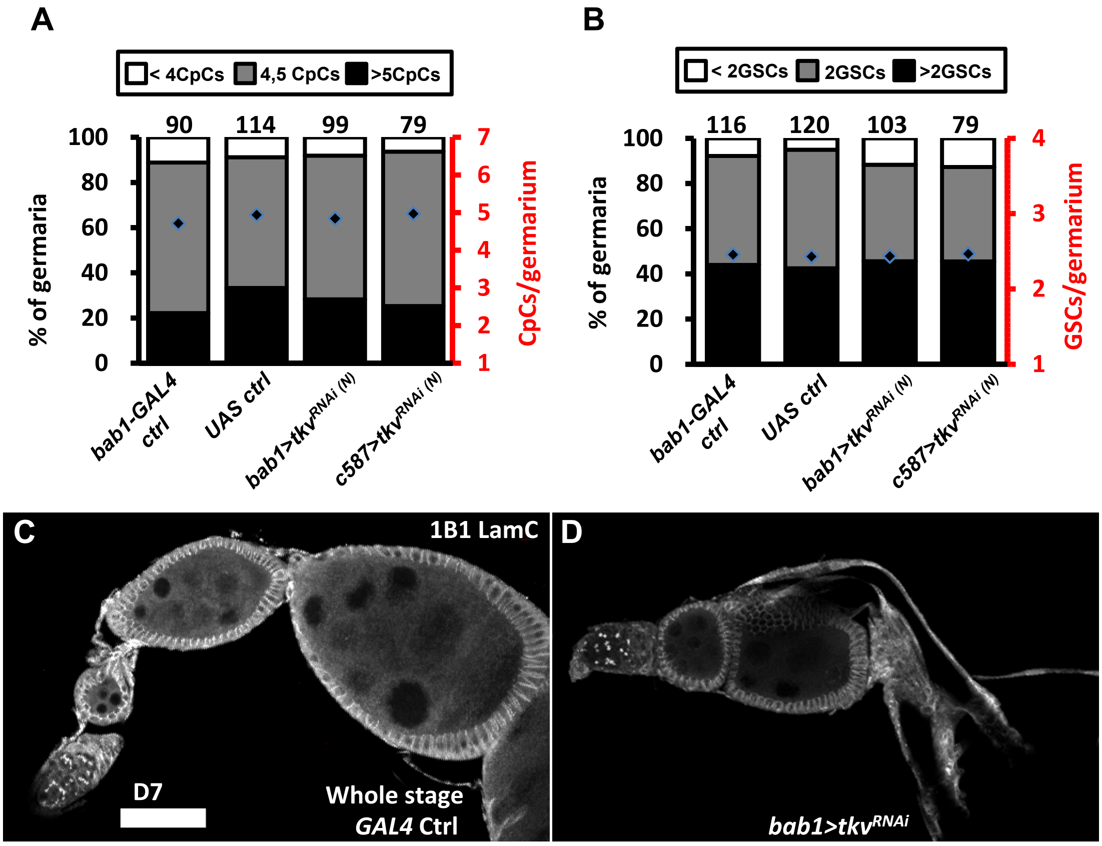

Supplementary Figure. 2

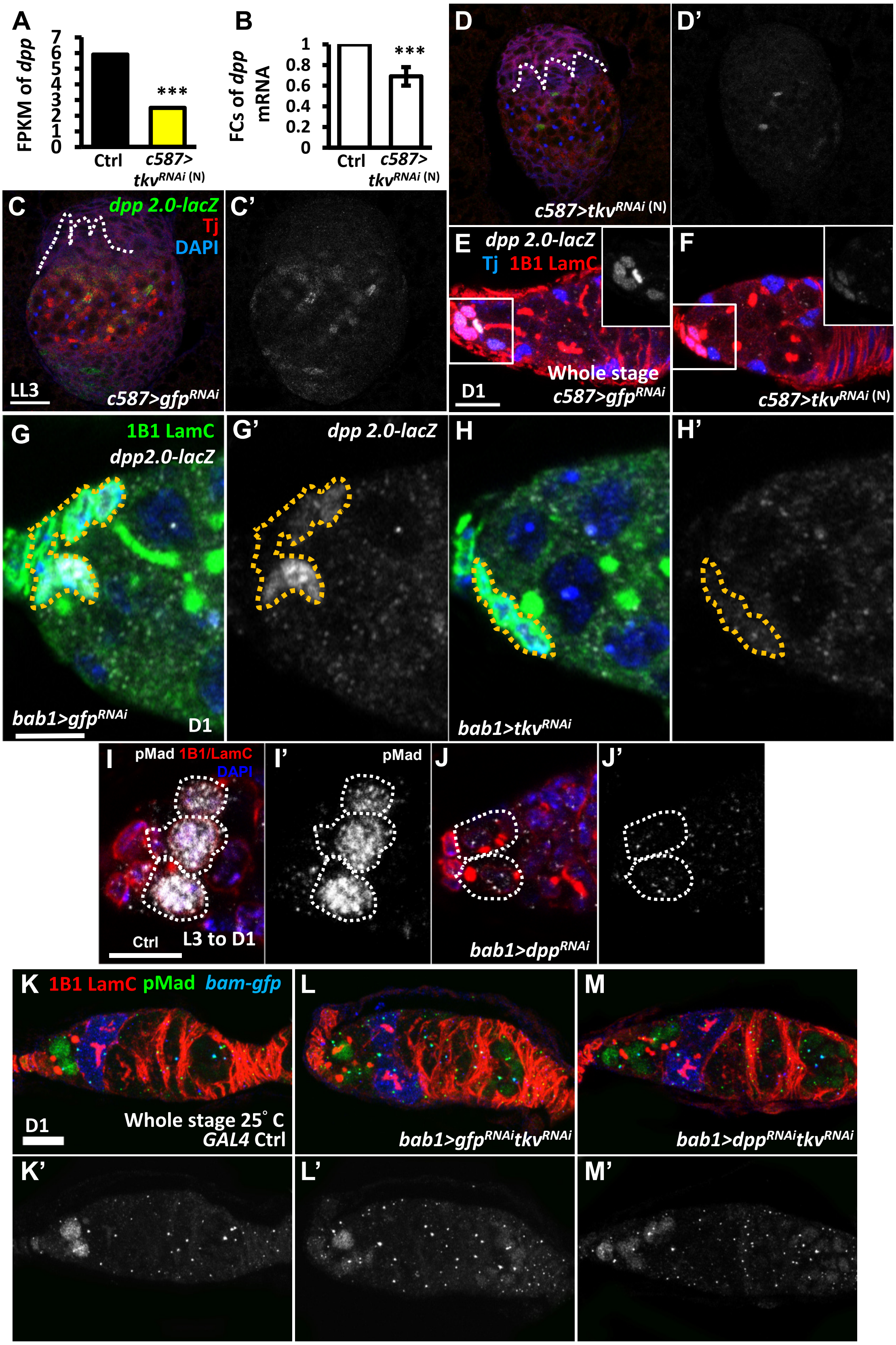

Supplementary Figure. 3

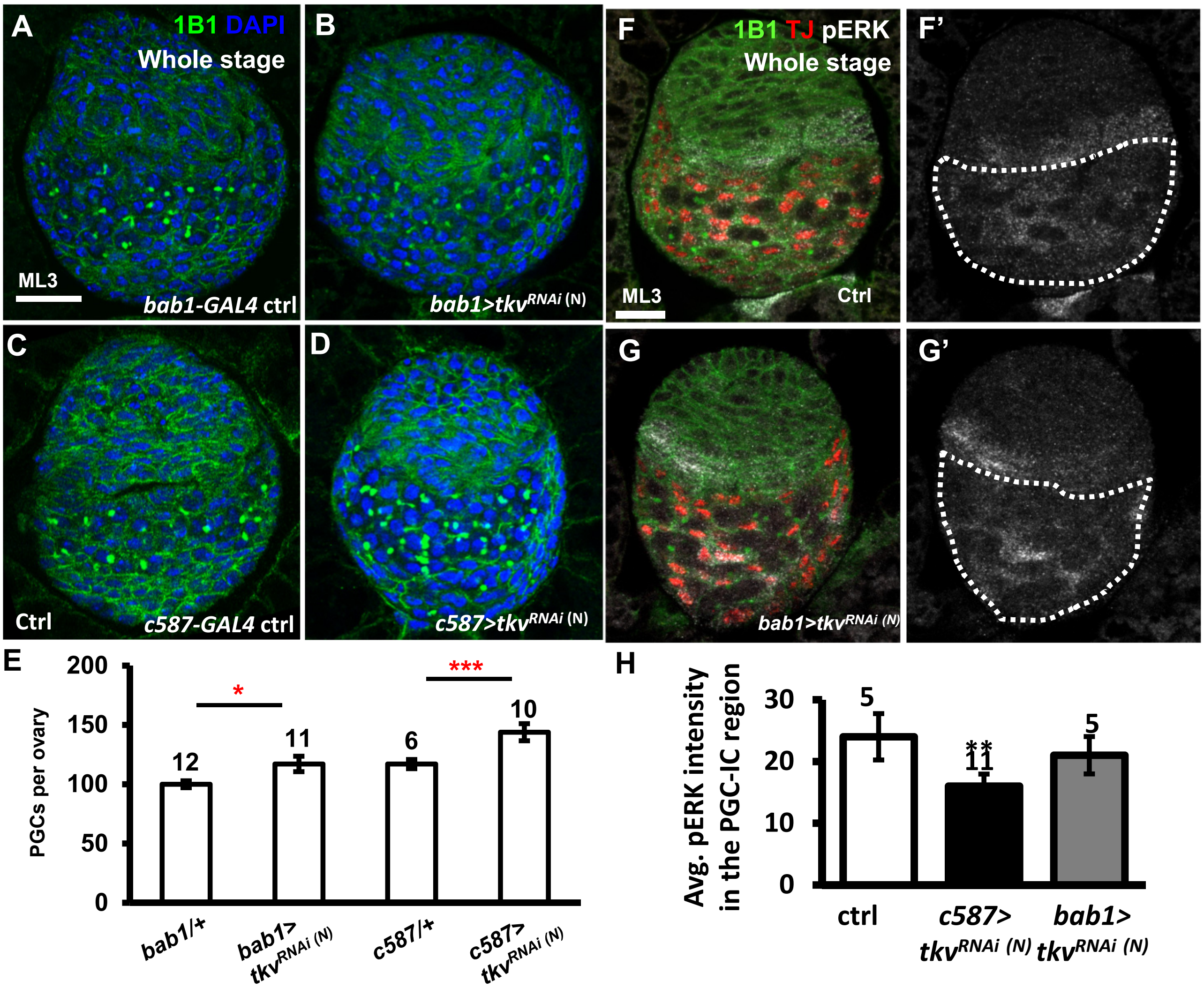

Supplementary Figure. 4

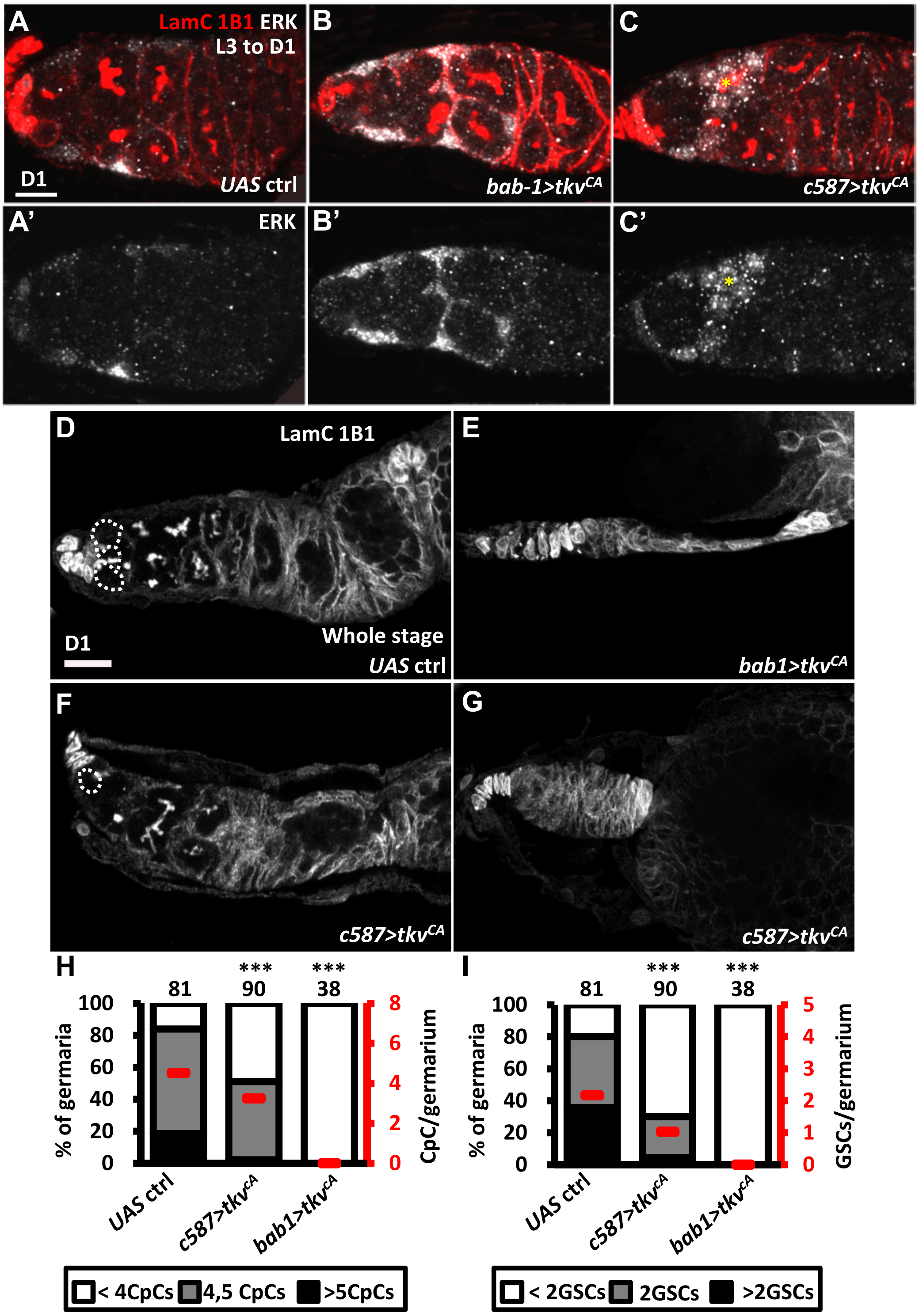

Supplementary Figure. 5

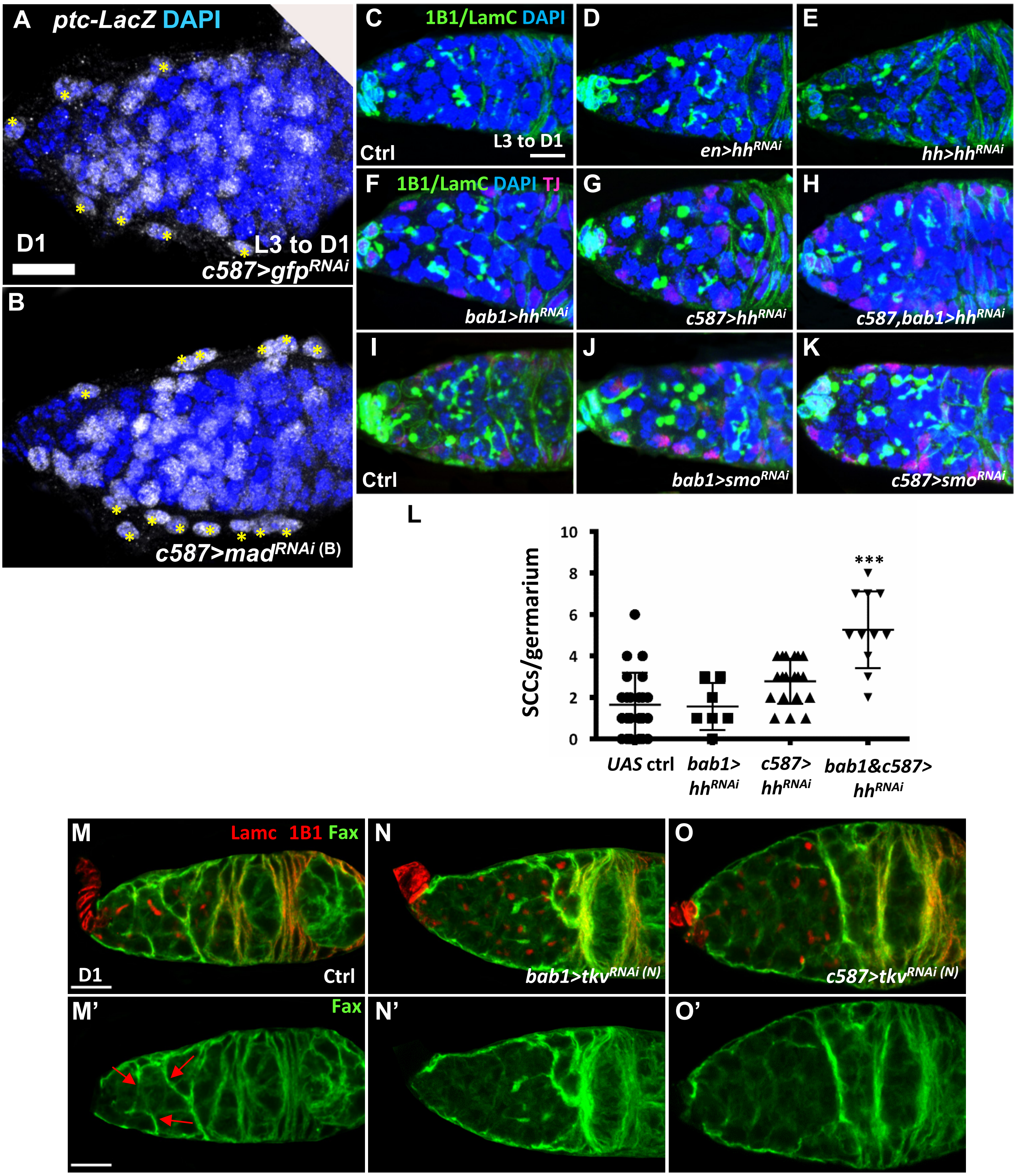

Supplementary Figure. 6
