# Supplementary material for: Smad-Independent BMP Signaling in Somatic Cells Limits the Size of the Germline Stem Cell Pool
Source: Stem Cell Reports. 2018 Aug 16;11(3):811–27. doi: 10.1016/j.stemcr.2018.07.008 (PMC6135924; doi:10.1016/j.stemcr.2018.07.008)
Supplement: Document S2. Article plus Supplemental Information [file mmc2.pdf]

Stem Cell Reports
Article

ISSCR

OPEN ACCESS

Smad-Independent BMP Signaling in Somatic Cells Limits the Size of the Germline Stem Cell Pool

Chen-Yuan Tseng,[1,6] Yu-Han Su,[1] Shun-Min Yang,[1] Kun-Yang Lin,[1,2,3] Chun-Ming Lai,[1,2,3] Elham Rastegari,[1,2,3] Oyundari Amartuvshin,[1] Yueh Cho,[1,2,3,7] Yu Cai,[4,5] and Hwei-Jan Hsu[1,2,3,*]

[1]Institute of Cellular and Organismic Biology, Academia Sinica, Taipei 11529, Taiwan
[2]Molecular and Biological Agricultural Sciences Program, Taiwan International Graduate Program, Academia Sinica and National Chung-Hsing University, Taipei 11529, Taiwan
[3]Graduate Institute of Biotechnology and Biotechnology Center, National Chung-Hsing University, Taichung 40227, Taiwan
[4]Temasek Life Science Laboratory, National University of Singapore, Singapore 117604, Singapore
[5]Department of Biological Sciences, National University of Singapore, Singapore 117543, Singapore
[6]Present address: Department of Biochemistry and Molecular Pharmacology, New York University School of Medicine, New York, NY 10016, USA
[7]Present address: Institute of Plant and Microbial Biology, Academia Sinica, Taipei 11529, Taiwan
*Correspondence: cohsu@gate.sinica.edu.tw
https://doi.org/10.1016/j.stemcr.2018.07.008

SUMMARY

In developing organisms, proper tuning of the number of stem cells within a niche is critical for the maintenance of adult tissues; however, the involved mechanisms remain largely unclear. Here, we demonstrate that Thickveins (Tkv), a type I bone morphogenetic protein (BMP) receptor, acts in the *Drosophila* developing ovarian soma through a Smad-independent pathway to shape the distribution of BMP signal within the niche, impacting germline stem cell (GSC) recruitment and maintenance. Somatic Tkv promotes Egfr signaling to silence transcription of Dally, which localizes BMP signals on the cell surface. In parallel, Tkv promotes Hh signaling, which promotes escort cell cellular protrusions and upregulates expression of the *Drosophila* BMP homolog, Dpp, forming a positive feedback loop that enhances Tkv signaling and strengthens the niche boundary. Our results reveal a role for non-canonical BMP signaling in the soma during GSC establishment and generally illustrate how complex, cell-specific BMP signaling mediates niche-stem cell interactions.

INTRODUCTION

The stem cell niche recruits an appropriate number of stem cells during organogenesis, and maintains stem cell homeostasis throughout the lifespan of an organism (Morrison and Spradling, 2008). However, the mechanisms that regulate the number of stem cells recruited to a niche remain unclear. To further understand this, we used the *Drosophila* ovary as a model because of its relatively simple architecture during developmental and adult stages, as well as its well-characterized germline stem cells (GSCs) and stem cell niche (Fuller and Spradling, 2007; Li and Xie, 2005; Moore et al., 1998).

Each adult ovary contains 16–20 ovarioles, which are the functional units of egg production. The anterior-most structure of the ovariole is called the germarium (Figure 1A, right panel). At the anterior tip of the germarium, a stem cell maintenance niche is formed by terminal filament (TF) cells, cap cells (the major component), and the anterior-most escort cells (ECs). This niche normally supports either two or three GSCs (Kirilly and Xie, 2007). Within each GSC is a special membrane-rich organelle, called the fusome, which is located adjacent to the interface between the GSC and cap cells. Each division of a GSC gives raise a cystoblast (CB), which undergoes four rounds of division to become 2-, 4-, 8-, and then 16-cell cysts. Each cell within the cyst is interconnected via a branched fusome. ECs that do not contact GSCs act as a differentiated cell niche that wraps germ cell cysts with long cellular processes to promote further germ cell differentiation (Kirilly et al., 2011; Morris and Spradling, 2011). Subsequently, cysts become surrounded by a monolayer of follicle cells, bud off from the germarium, and then develop into mature eggs (Margolis and Spradling, 1995).

In the late-third-instar larvae (LL3) stage (Figure 1A, left panel), the GSC niche becomes established within the gonad and recruits GSCs from a pool of virtually identical undifferentiated primordial germ cells (PGCs), which carry rounded fusomes and associate with somatic intermingled cells (ICs). These cells were previously shown to require Hh signaling to maintain interactions with somatic cells (Lai et al., 2017). The anterior-most ICs differentiate into cap cells (Hsu and Drummond-Barbosa, 2011; Lai et al., 2017; Song et al., 2007), which provide E-cadherin-mediated anchorage and stemness signals to select nearby PGCs for the GSC pool (Song et al., 2002). The remaining ICs become ECs that promote PGC differentiation (Lai et al., 2017).

Bone morphogenetic protein (BMP) signaling is highly conserved and controls a variety of developmental processes, as wells as stem cell maintenance during tissue homeostasis (Hamaratoglu et al., 2014; Wang et al., 2014). BMP signals may act through canonical, Smad-dependent, or non-canonical pathways. In the Smad-dependent signaling pathway, BMPs bind to a heterotetrameric

This is an open access article under the CC BY license (http://creativecommons.org/licenses/by/4.0/).

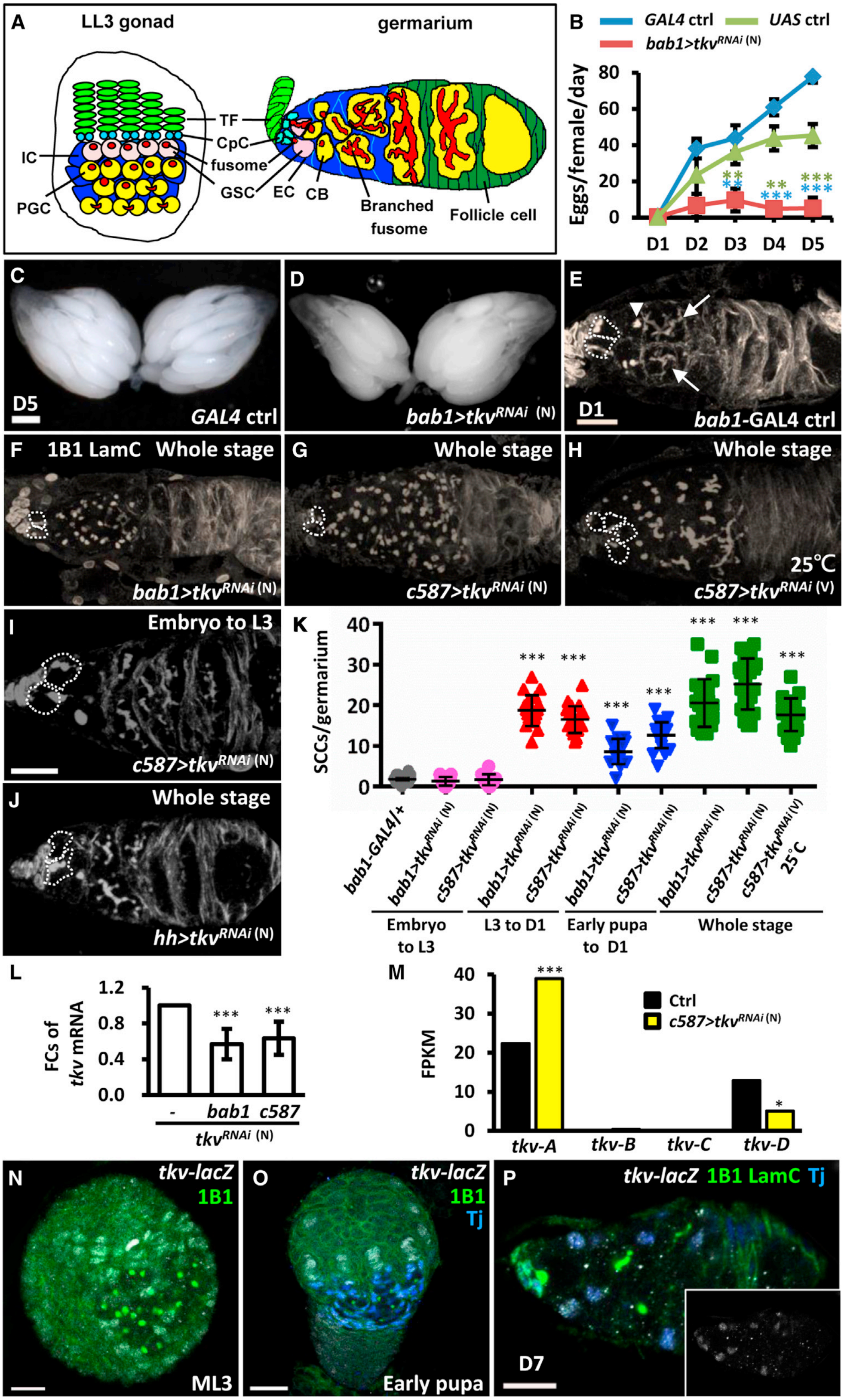

(legend on next page)

complex of type I and type II receptors. After ligand binding, type II receptors transphosphorylate type I receptors, and the activated type I receptors phosphorylate receptor-regulated Smads (R-Smads). Phosphorylated R-Smads will then associate with a common partner (Co)-Smad, and the complex will translocate to the nucleus, where it regulates gene expression. In the *Drosophila* ovary, the BMP homolog, Decapentaplegic (Dpp), is the major niche-derived stemness factor for GSC recruitment and maintenance. GSCs express Saxophone (Sax) and Thickveins (Tkv) as type I receptors and Punt as a type II receptor. To restrict delivery of the Dpp signal to GSCs, niche cap cells also express Division abnormally delayed (Dally), which is a glypican protein that binds and stabilizes Dpp on the extracellular matrix. After binding to receptors on GSCs, the Dpp signal is transmitted to Mothers against Dpp (Mad, R-Smad), which forms a complex with Medea (Med, Co-Smad) to silence transcription of Bag of marbles (Bam), a differentiation factor. While the canonical signaling pathway is the only previously identified mechanism by which Dpp regulates GSCs in the *Drosophila* ovary, BMP signaling is widely known to modulate gene expression in other biological systems via various non-canonical pathways, including the mitogen-activated protein kinase cascade (Wang et al., 2014).

In this study, we found that, in addition to its known role in maintaining GSC identity via Smad signaling, Tkv plays a crucial role in gonadal somatic ICs to confine the Dpp signaling zone for GSC recruitment via a non-Smad pathway during ovary development. Tkv was present in TF and ICs of larval gonads, while in the adult ovary, Tkv was observed in TF, cap and ECs of the germarium. Silencing *tkv* expression in larval gonadal somatic cells resulted in the appearance of ectopic GSCs at the adult; however, gene silencing in TF cells did not. Instead of signaling through BMP canonical proteins, genetic and RNA sequencing (RNA-seq) analyses revealed that ICs expressing Tkv had activated Hh and Egfr signaling in parallel to limit GSC number. As such, evidence of Hh and Egfr signaling was absent in gonads carrying *tkv*-knockdown (*tkvKD*) somatic cells. Moreover, compensatory stimulation of either Hh or Egfr signaling partially prevented the formation of ectopic GSCs in *tkvKD* background, but disruption of either signaling pathway did not affect signaling through the other pathway. Based on these findings, we conclude that Dpp signaling in the GSC niche shapes the Dpp tissue distribution via non-canonical modulation of Hh and Egfr signaling to limit the number of GSCs recruited to the niche.

RESULTS

Tkv-Expressing Somatic Cells Promote Germ Cell Differentiation for Functional Reproduction

To identify genes in the soma that control germ cell homeostasis during ovary development, we performed a genetic screen using transgenic *UAS-RNAi* lines from the National Institute of Genetics (N). *RNAi* expression was

Figure 1. Tkv Expression in the Soma Controls Germ Cell Differentiation for Egg Production

(A) Cross-sectional diagrams show a late-L3 (LL3) larval gonad (left) and an adult germarium (right). TF, terminal filament cells; PGC, primordial germ cell containing spectrosomes (round-shaped fusome); IC, intermingled cells; GSC, germline stem cell. PGCs in close proximity to the niche become GSCs, while those further away from the niche initiate differentiation programs (yellow). Dividing PGCs are identified by the presence bar-shaped fusomes. At the end of the LL3 stage, niche cap cells (CpCs, blue) begin to form. During the pupal stage, ICs are incorporated into the germarium and named ECs. GSC progeny, cystoblast (CB) undergoes four rounds of incomplete division to form 16-cell cysts; each cell within the cyst is interconnected with a branched fusome.

(B) The average number of eggs produced in a day (D) is shown for newly eclosed *GAL4* control (ctrl), *UAS* control, and *bab1>tkv*$^{RNAi\ (N)}$ females from days 1–5.

(C and D) Day 5 control (C) and *bab1>tkv*$^{RNAi\ (N)}$ (D) ovaries.

(E–J) One-day-old *bab1-GAL4* control (E), *bab1>tkv*$^{RNAi\ (N)}$ (whole-stage knockdown) (F), *c587>tkv*$^{RNAi\ (N)}$ (whole-stage knockdown) (G), *c587>tkv*$^{RNAi\ (V)}$ (25°C, whole-stage knockdown) (H), *c587>tkv*$^{RNAi\ (N)}$ (embryo to mid-L3 [ML3] knockdown) (I), and *hh > tkv*$^{RNAi\ (N)}$ (whole-stage knockdown) (J) germaria with 1B1 (gray, fusomes) and LamC (gray, TF and CpC nuclear envelopes). The arrowhead indicates a spectrosome (round-shaped fusome), and the arrows indicate branched fusomes.

(K) Number of spectrosome-containing cells (SCCs) in the germaria of control and *tkvKD* flies driven by *bab1-GAL4* or *c587-GAL4* from embryo to ML3, ML3 to newly eclosed (D1), early pupal to D1 or whole stage.

(L) qRT-PCR analysis (fold changes [FCs]) of total *tkv* mRNA in 1-day-old control, *bab1>tkv*$^{RNAi\ (N)}$ and *c587>tkv*$^{RNAi\ (N)}$ germaria.

(M) RNA-seq-based gene expression values (fragments per kilobase of transcript per million mapped reads [FPKM]) for *tkv* isoforms, *tkv-A-D*, in 1-day-old control and *c587>tkv*$^{RNAi\ (N)}$ germaria. Statistics analysis was from two biological replicates.

(N–P) ML3 (N), early pupa (O), and 7-day-old germarium (P) with *tkv-lacZ* (gray), 1B1 (green), Tj (blue, ICs in O and ECs in P), and LamC (green) labeling. Dashed circles mark GSCs. The insert plane in (P) shows only the *tkv-lacZ* channel.

Scale bars, 1 mm (C) and 10 μm (E, I, and N–P). Error bars are SE and in (B) and (L) were from three independent experiments; *p < 0.05, **p < 0.01, ***p < 0.001. Knockdown experiments were carried out at 29°C, unless otherwise indicated. Genotypes of control flies are *bab1-GAL4*/+ or *UAS-tkv*$^{RNAi\ (N)}$/+.

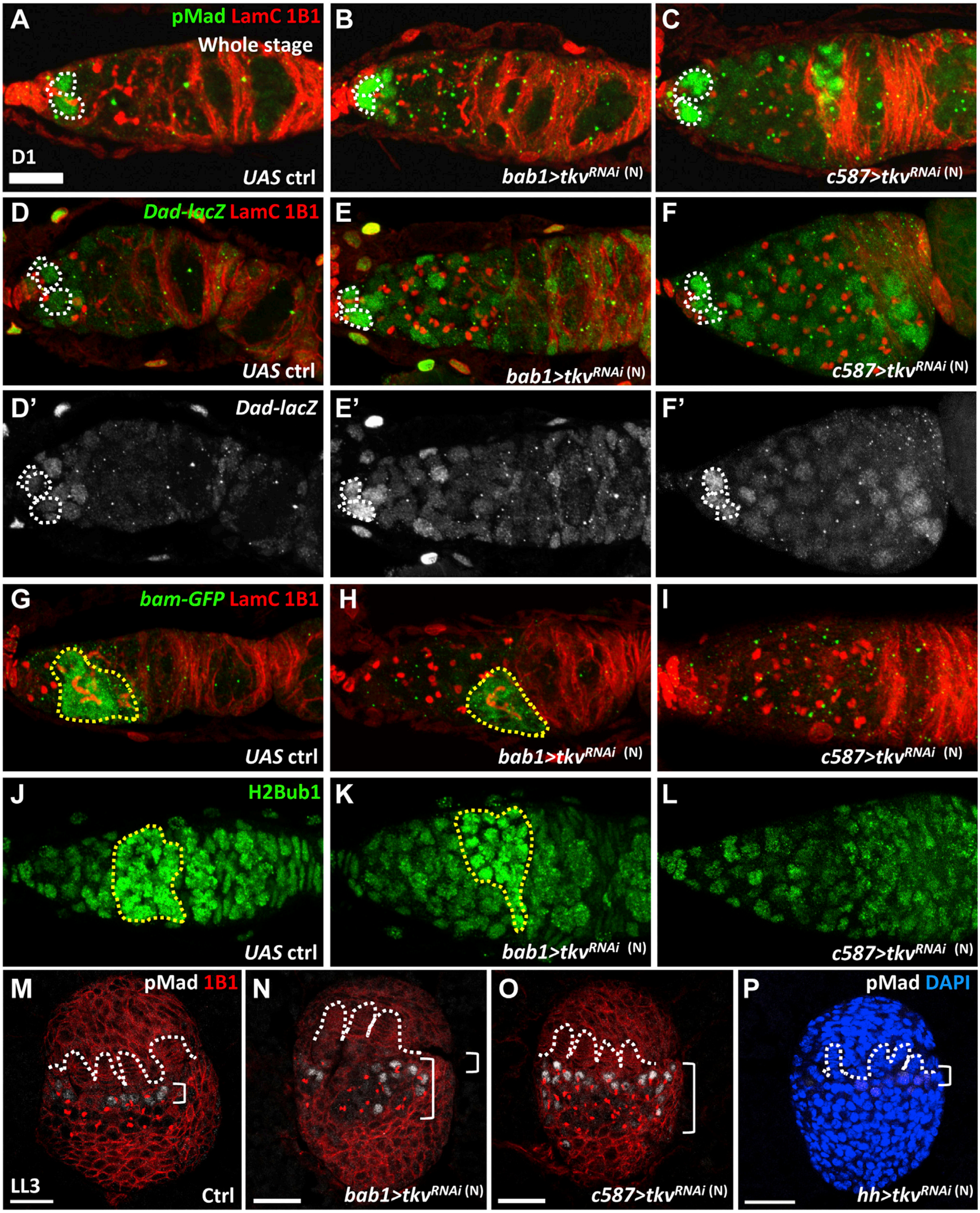

(legend on next page)

driven by *bab1-GAL4*, which is expressed in all somatic cells, but specifically in TF cells, cap cells, and anterior ECs at the late pupal and adult stages (Figures S1A, S1C, S1E, S1G, and S1I) (Lai et al., 2017). We found that somatic knockdown of Tkv impaired egg production in the adult (Figure 1B), without affecting overall ovary morphology (Figures 1C and 1D). These results indicate that Tkv-mediated signaling in the soma is required for functional reproduction.

Because no obvious morphological defects were observed in *tkvKD* ovaries, we more closely examined germ cells in somatic *tkvKD* germaria. Control germaria (n = 16) of newly eclosed (D1) flies carried two to three GSCs with anterior anchoring of fusomes that were directly adjacent to niche cap cells. Each germarium also carried 2 ± 1.1 CBs, the immediate daughter cells of GSCs, which were identified by the presence of a spherical fusome (spectrosome-containing cells), but were positioned distal to niche cap cells (Figure 1E). Interestingly, knockdown of *tkv* throughout development using *bab1-GAL4* caused an accumulation of SCCs (20 ± 5.6, n = 20 germaria) (Figures 1F and 1K), suggesting a failure of germ cell differentiation. Similar phenomena were observed with another somatic driver, *c587-GAL4* (Figures 1G and 1K), which was also expressed in almost all somatic cells but restricted in ECs (Figures S1B, S1D, S1F, and S1J), or by an independent tkv^{RNAi} line (Figures 1H and 1K). However, knockdown of *tkv* before the mid-L3 stage using *bab1* or *c587-GAL4*, or using the TF driver, *hh-GAL4* (Figures S1K and S1L), did not result in SCC accumulation (Figures 1I–1K). Notably, knockdown of *tkv* in the soma throughout development also did not affect GSC and niche cap cell numbers, and egg chamber morphology (Figure S2).

Tkv-D Functions in ICs to Promote Germ Cell Differentiation

Although qRT-PCR results showed that *tkv* mRNA transcripts were reduced in 1-day-old $bab1>tkv^{RNAi\ (N)}$ and $c587>tkv^{RNAi\ (N)}$ ovaries (Figure 1L), RNA-seq results revealed that among four *tkv* transcript variants only the level of *tkv-D* transcripts was reduced in somatic *tkvKD* germaria (Figure 1M). Interestingly, *tkv-B* and *tkv-C* expression levels were extremely low, and *tkv-A* mRNA was increased approximately 2-fold in somatic *tkvKD* germaria compared with control (Figure 1M). This result suggests that *tkv-D* may be mainly expressed in the soma, while *tkv-A* may be predominately expressed in germ cells. Furthermore, the germ cell expression of *tkv-A* appears to have somehow been affected by *tkv-D* knockdown in somatic cells. The four different *tkv* transcripts share identical sequences in the coding region and 3′ UTR, while the 5′ UTRs were variable (see Supplemental Information), suggesting that transcription of *tkv-A* and *tkv-D* isoforms may be under the control of different regulatory elements. We further examined *tkv-D* expression during ovary development using a transcriptional reporter, *P2-LacZ*, in which the LacZ reporter is inserted behind the promoter of *Tkv-B*, *-C*, and *-D* (Luo et al., 2015). We found that *P2-lacZ* was highly expressed in TFs and ICs at mid-L3 and early pupal stages (Figures 1N and 1O), but expression was restricted in cap cells and ECs of adult germaria (Figure 1P). These results indicate that Tkv-D functions in ICs for proper germ cell differentiation.

Disruption of Tkv in the Developing Soma Expands BMP Signaling Territory and Leads to Formation of Ectopic GSCs

We further investigated the fate of ectopic SCCs in somatic *tkvKD* germaria. In GSCs, the binding of BMP ligands induces the phosphorylation of Mad (pMad), which translocates to the nucleus, where it activates expression of *daughter against Dpp* (*dad*) and suppresses *bam* transcription (Harris and Ashe, 2011). Therefore, we examined the levels of pMad, Dad (revealed by *dad-lacZ*), and Bam (revealed by *bam::bam-GFP* [Chen and McKearin, 2003]) in 1-day-old control and $bab1>tkv^{RNAi\ (N)}$ and $c587>tkv^{RNAi\ (N)}$ germaria. In the control germaria (Figure 2A), pMad was observed in GSCs (Figure 2A), while, in somatic *tkvKD* germaria, pMad expression levels were increased in GSCs and also detected in germ cells outside the niche (Figures 2B and 2C). Similarly, *dad-lacZ* expression was high in GSCs and CBs in the control germarium (Figure 2D), but, in somatic *tkvKD*

Figure 2. Somatic Tkv Constrains Canonical BMP Signaling to GSCs

(A–F) One-day-old control (ctrl) (A and D), $bab1>tkv^{RNAi\ (N)}$ (B and E), and $c587>tkv^{RNAi\ (N)}$ germaria (C and F) with 1B1 (red, fusomes), LamC (red, TF and cap cell nuclear envelopes), pMad in (A)–(C) (green), and *Dad-lacZ* in (D)–(F) (green, a BMP signaling reporter). (D′)–(F′) show only *Dad-lacZ* expression in gray. Dashed white circles mark GSCs.

(G–I) One-day-old control (G), $bab1>tkv^{RNAi\ (N)}$ (H), and $c587>tkv^{RNAi\ (N)}$ germaria (I) with 1B1 (red), LamC (red), and Bam-GFP (green, differentiating cysts marked by dashed yellow circles).

(J–L) One-day-old control (J), $bab1>tkv^{RNAi\ (N)}$ (K), and $c587>tkv^{RNAi\ (N)}$ germaria (L) with histone H2B mono-ubiquitination (H2Bub1) (green, differentiating cysts marked by dashed yellow circles).

(M–P) Late-L3 (LL3) control (M), $bab1>tkv^{RNAi\ (N)}$ (N), $c587>tkv^{RNAi\ (N)}$ (O), and $hh > tkv^{RNAi\ (N)}$ larval gonads (P) with pMad (gray), 1B1 in (G)–(I) (red, fusomes), and DAPI in (J) (blue). Dashed lines mark TFs, brackets indicate the region containing GSCs. Scale bar, 10 μm. The genotype of controls in (A), (D), (G), (J), and (M) is $UAS\text{-}tkv^{RNAi\ (N)}/+$.

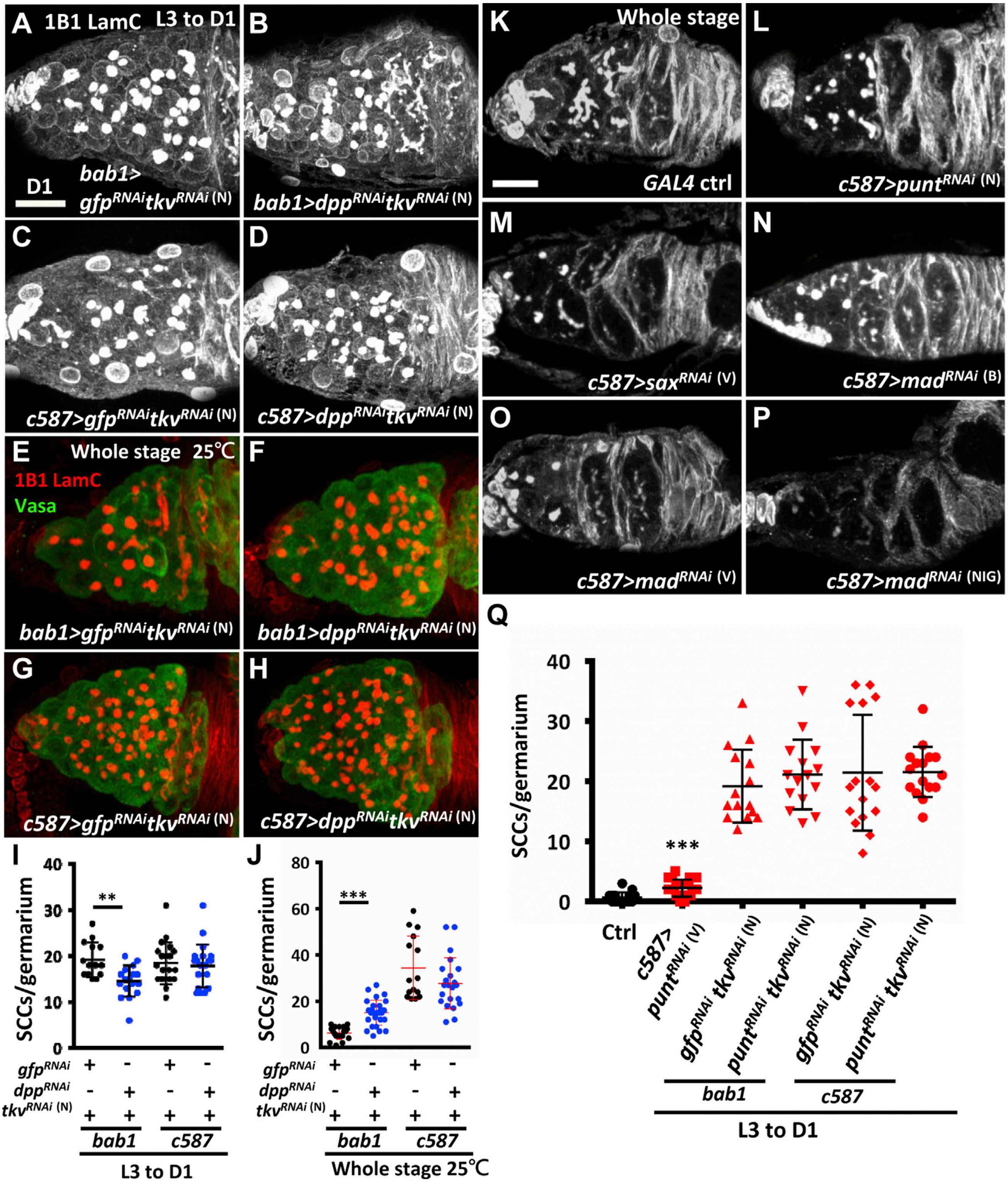

Figure 3. Dpp-Tkv-Mediated Signaling in the Soma Limits GSC Number via a Smad-Independent Pathway

(A–D) One-day-old germaria with *gfp*^{RNAi}*tkv*$^{RNAi\ (N)}$ (A and C) and *dpp*^{RNAi}*tkv*$^{RNAi\ (N)}$ (B and D) knockdown by *bab1-GAL4* (A and B) or *c587-GAL4* (C and D) from ML3 to adult (D1) stage. Germaria were stained for 1B1 (gray, fusomes) and LamC (gray, TF and cap cell nuclear envelopes). Co-knockdown of *dpp* and *tkv* in the niche by *bab1-GAL4* reduced the number of SCCs.

(legend continued on next page)

germaria, expression was greatly increased in GSCs and detected in most germ cells (Figures 2E and 2F). In both control and somatic *tkvKD* germaria, differentiated germ cells with branched fusomes exhibited *bam-GFP* expression, but this expression was absent in SCCs from somatic *tkvKD* germaria (Figures 2G–2I). It was previously reported that monoubiquitinated histone H2B (H2Bub1) is absent from GSCs and pre-CBs (König and Shcherbata, 2015). To further characterize the aberrant SCCs in somatic *tkvKD* germaria, we examined the expression of H2Bub1. Surprisingly, in control germaria, we detected H2Bub1 expression in GSCs, and its expression reached the highest levels in the differentiating germline (Figure 2J). In somatic *tkvKD* germaria (Figures 2K and 2L), GSCs and germ cells outside of the niche displayed similar H2Bub1 expression levels. These results indicate that the SCCs accumulated in *tkvKD* germaria may be GSCs or immediate GSC progeny with active BMP signaling and suppressed Bam expression maintaining them in an undifferentiated state.

We also observed an expansion of pMad-positive germ cells in *bab1>tkv*$^{RNAi\ (N)}$ and *c587>tkv*$^{RNAi\ (N)}$ ovaries at the LL3 stage (Figures 2N and 2O), compared with control or *hh > tkv*^{RNAi} gonads (Figures 2M and 2P). These results suggest that BMP signaling is elevated and spreads to germ cells outside of the niche when Tkv is eliminated from ovarian somatic cells during development.

SCC Accumulation Is Not Due to Excessive Dpp Production

Because of the widespread BMP signaling in the accumulated SCCs, we sought to test whether Dpp, a major BMP ligand, was upregulated in *tkvKD* ovaries. Therefore, we examined *dpp* expression in control and *c587>tkv*$^{RNAi\ (N)}$ or *bab1>tkv*$^{RNAi\ (N)}$ germaria by qRT-PCR, RNA-seq, and a *dpp* transcriptional reporter, *dpp2.0-lacZ* (Luo et al., 2017). By all three methods, we found that *dpp* expression was decreased in *tkvKD* ICs and adult cap cells (Figures S3A–S3H). We also disrupted *dpp* expression in somatic *tkvKD* germaria using a *dpp*^{RNAi} line. Functional *dpp* knockdown in this line was validated by pMad staining (Figures S3I and S3J). Co-knockdown of *dpp* and *tkv* by *c587-GAL4* did not reduce the number of SCCs, and co-knockdown of *dpp* and *tkv* by *bab1-GAL4* only slightly reduced SCC number (Figures 3A–3D and 3I). Thus, we conclude that elevation of Dpp levels is not a primary cause of SCC accumulation in somatic *tkvKD* germaria, although Dpp may be distributed outside of the niche. Further, the differential expression of cell types between the two *GAL4* lines to rescue SCC accumulation supports the previously proposed model that niche cap cells are the major source of Dpp production (Liu et al., 2015), regardless of whether the expression of *dpp 2.0-lacZ* is present in ICs of larval gonads (see Figure S3).

Non-canonical Dpp-Tkv Signaling in the Soma Promotes Germ Cell Differentiation

In the canonical BMP signaling pathway, Punt or Sax forms a complex with Tkv and transmits Dpp signals to Mad by phosphorylation (Hamaratoglu et al., 2014). To dissect the Tkv-mediated signaling pathway that functions in the soma to promote germ cell differentiation, we first asked if Dpp is required. We moderately reduced *dpp* and *tkv* expression by driving *RNAi* expression at a lower temperature (25°C instead of 29°C). Knockdown of *tkv* alone, using *bab1-GAL4*, still resulted in few SCCs (6.2 ± 2.7, n = 20). Interestingly, simultaneous knockdown of *tkv* and *dpp* using *bab1-GAL4* dramatically increased the number of SCCs (15.1 ± 5.4, n = 31, $p < 0.001$) (Figures 3E, 3F, and 3J), which were p-Mad-positive germ cells (Figures S3K–S3M). This synergistic effect on SCC accumulation suggests that the two factors function in the same general pathway. In contrast, disruption of *tkv* with or without *dppKD* using *c587-GAL4* did not produce any differences in SCC number (Figures 3G, 3H, and 3J). This result indicates that Dpp is mainly produced by niche cap cells, and activates Tkv in the soma to promote germ cell differentiation. We also disrupted expressions of *punt*, *sax*, or *mad* in the soma. Knockdown of *punt* only resulted in a small increase of SCC number, compared with controls (Figures 3K, 3L, and 3Q); co-knockdown of *punt* and *tkv* in the soma also appeared to have slightly increased SCC number, but the difference did not reach statistical significance. In addition, knockdown of *sax* or *mad* did not produce observable SCC accumulation (Figures 3M–3P). Since the accumulation of SCCs was dependent on *dpp* expression, but not the expression of canonical signaling molecules, the results

(E–H) One-day-old germaria with *gfp*^{RNAi}*tkv*$^{RNAi\ (N)}$ (E and G) and *dpp*^{RNAi}*tkv*$^{RNAi\ (N)}$ (F and H) knockdown by *bab1-GAL4* (E and F) or *c587-GAL4* (G and H) throughout development at 25°C. Germaria were stained for 1B1 (red), LamC (red), and Vasa (green, germ cells).
(I and J) Number of SCCs per germarium in the indicated RNAi knockdown genotypes, either from ML3 to D1 (I) or throughout all developmental stages (25°C) (J).
(K–P) One-day-old control (ctrl) germaria (K) and those with *punt*$^{RNAi\ (L)}$ (L), *Sax*$^{RNAi\ (V)}$ (M), and *mad*$^{RNAi\ (B,\ V,\ and\ NIG)}$ (N–P) knockdown by *c587-GAL4* throughout all developmental stages. Germaria were stained for 1B1 and LamC as in (A)–(D).
(Q) Number of SCCs per germarium in the indicated RNAi knockdown genotypes from ML3 to D1. Knockdown experiments were carried out at 29°C, except when otherwise indicated. The genotype of the control in (K) and (Q) is *c587-GAL4/+*. Scale bars, 10 μm. Error bars indicate SE; $^{**}p < 0.01$, $^{***}p < 0.001$.

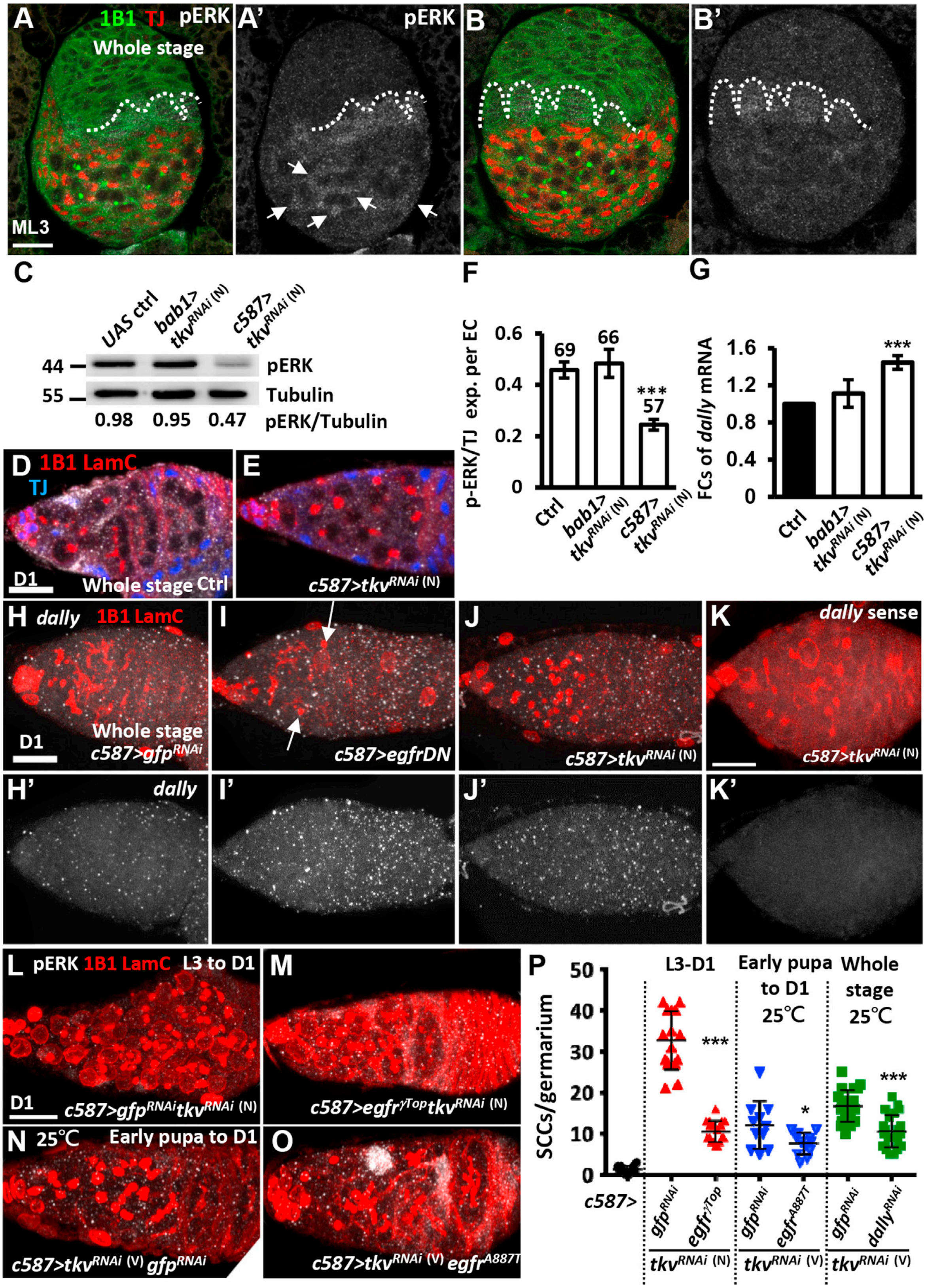

(legend on next page)

suggest that Dpp-Tkv signaling controls germ cell differentiation via a non-canonical BMP signaling mechanism.

Tkv Restricts GSC Recruitment via the Egfr-Dally Regulatory Axis

Egfr signaling in ICs negatively regulates the number of PGCs in order to balance the soma and germ cell populations (Gilboa and Lehmann, 2006). In addition, Egfr signaling is known to suppress expression of *dally* (encodes a glypican that facilitates distribution of Dpp) to sharpen the BMP gradient in the larval gonad, such that the first row of PGCs (next to TFs) will be recruited and maintained as GSCs (Guo and Wang, 2009; Matsuoka et al., 2013). We found that somatic *tkvKD* gonads in mid-L3 exhibited a small increase in PGC number (Figures S4A–S4E), without obvious changes in the number of ICs (Tj-positive cells in Figures 4A and 4B). Egfr signaling, as indicated by phospho (p)ERK staining (Gabay et al., 1997), was reduced in ICs of mid-L3 *c587>tkv*$^{RNAi\ (N)}$ gonads (Figures 4A–4C and S4F–S4H), and in ECs of the adult *c587>tkv*$^{RNAi\ (N)}$ germarium (Figures 4D–4F). Surprisingly, pERK expression was not affected in *bab1>tkv*$^{RNAi\ (N)}$ ovaries (Figures 4C, 4F, and S4F–S4H). We speculate that this difference was due to *GAL4* expression differences between the two lines, as expression of *c587-GAL4* was higher in ICs from late-L3 to adult stages when compared with *bab1-GAL4* (see Figure S1). In contrast, overexpressing a constitutively active form of *tkv* (*tkv*^{CA}) in somatic gonadal precursors after the mid-L3 stage driven by either *bab1*- or *c587-GAL4* caused an elevation of Egfr signaling in adult ECs (Figures S5A–S5C). In addition, germaria with *tkv*^{CA} overexpression, driven by either *bab1-GAL4* or *c587-GAL4* from mid-L3 to adult (data not shown) or throughout development (Figures S5D–S5I), were devoid of GSCs and exhibited an associated loss of cap cells. This observation was in agreement with the idea that high Egfr signaling depletes the PGC pool (Matsuoka et al., 2013). However, the reduction of cap cells may also contribute to the observed loss of GSCs. Nevertheless, this result suggests that Tkv-mediated signaling restricts GSC number, at least partially through Egfr signaling.

As expected, *dally* transcript levels, which are negatively regulated by Egfr signaling (Liu et al., 2010), were increased in *c587>tkv*$^{RNAi\ (N)}$ ovaries as compared with control and *bab1>tkv*$^{RNAi\ (N)}$ (Figure 4G). We confirmed this qRT-PCR result with *in situ* hybridization in 1-day-old germaria. Compared with control (Figure 4H), *dally* mRNA was increased in the germaria that overexpressed a dominant-negative form of Egfr (*egfr*^{DN}) or *tkvKD* driven by *c587-GAL4* (Figures 4I and 4J). Probing *c587>tkv*$^{RNAi\ (N)}$ germaria with *dally* sense probes did not show any signal (Figures 4K), confirming the specificity of *dally* anti-sense probes used in this experiment.

To directly test if Egfr signaling acts downstream of Tkv to limit GSC number, we knocked down *tkv* with concurrent overexpression of a constitutively active form of Egfr, *egfr*$^{\lambda Top}$ (Queenan et al., 1997) with *c587-GAL4* from L3 to adult stages, and examined the SCC number in 1-day-old germaria (Figures 4L, 4M, and 4P). Our results showed that SCC accumulation was prevented by forcing Egfr signaling (Figure 4P). Knockdown of *tkv* with coincident overexpression of another constitutive active form of Egfr, *egfr*^{A887T} (Lesokhin et al., 1999), from pupal to adult stages (Figures 4N and 4O), or co-knockdown of *tkv* and *dally* throughout developmental stages by *c587-GAL4* also significantly reduced SCC number (Figure 4P). These

Figure 4. Dpp Signaling in the Soma Limits the GSC Number via Egfr Signaling

(A and B) ML3 control (ctrl) (A) and *c587>tkv*$^{RNAi\ (N)}$ (B) gonads with 1B1 (green, fusomes), Tj (red, ICs), and phospho (p)ERK (gray). Dashed line indicates TFs. (A′) and (B′) show only pERK channel. Arrows in (A) point to ICs with strong pERK signals.

(C) Representative pERK (42/44 kDa) western blots of control, *bab1>tkv*$^{RNAi\ (N)}$, and *c587>tkv*$^{RNAi\ (N)}$ ML3 gonads. α-Tubulin (55 kDa) was used as an internal control. Molecular weight markers are indicated to the left of the blots. Ratio of pERK to α-tubulin expression is shown below the blot.

(D and E) One-day-old control (ctrl) and *c587>tkv*$^{RNAi\ (N)}$ germaria with 1B1 (red), LamC (red, cap cell nuclear envelopes), Tj (blue, ECs), and pERK (gray) labeling.

(F) Ratio of pERK to Tj expression per EC is shown for 1-day-old control, *bab1>tkv*$^{RNAi\ (N)}$, and *c587>tkv*$^{RNAi\ (N)}$ flies. Number of ECs analyzed are shown above each bar.

(G) qRT-PCR analysis revealed FCs of *dally* mRNA in 1-day-old control, *bab1>tkv*$^{RNAi\ (N)}$, and *c587>tkv*$^{RNAi\ (N)}$ germaria.

(H–K) One-day-old *c587>gfp*^{RNAi} (H), *c587>egfrDN* (I), and *c587>*tkv$^{RNAi\ (N)}$ germaria (J and K) with 1B1 (red), LamC (red), anti-sense *dally* RNA probe in (H)–(J) (gray), and sense *dally* probe in (K) (gray). Arrows point to spectrosomes. In (H′)–(K′) only the *in situ* staining for *dally* is shown.

(L–O) One-day-old *c587>gfp*^{RNAi}*tkv*$^{RNAi\ (N)}$ (L), *c587>egfr*$^{\gamma Top}$*tkv*$^{RNAi\ (N)}$ (M), *c587> tkv*$^{RNAi\ (V)}$ *gfp*^{RNAi} (N), and *c587> tkv*$^{RNAi\ (V)}$*egfr*^{A887T} germaria (O) with 1B1 (red), LamC (red), and pERK (gray).

(P) Number of SCCs in control germaria and those expressing *tkv*^{RNAi} plus gfp^{RNAi}, *egfr*$^{\gamma Top}$, *egfr*^{A88T}, or *dally*^{RNAi} driven by *c587-GAL4* from ML3 to D1, pupa to D1 stages, or throughout development (whole stage). Knockdown experiments were carried out at 29°C, except where otherwise indicated. The genotype of the control in (A) and (D) is *c587-GAL4/+*. Scale bars, 20 μm (A) and 10 μm (D, H, K, and L). Error bars show SE and in (G) is from at least three independent experiments: $^{*}p < 0.05$, $^{***}p < 0.001$.

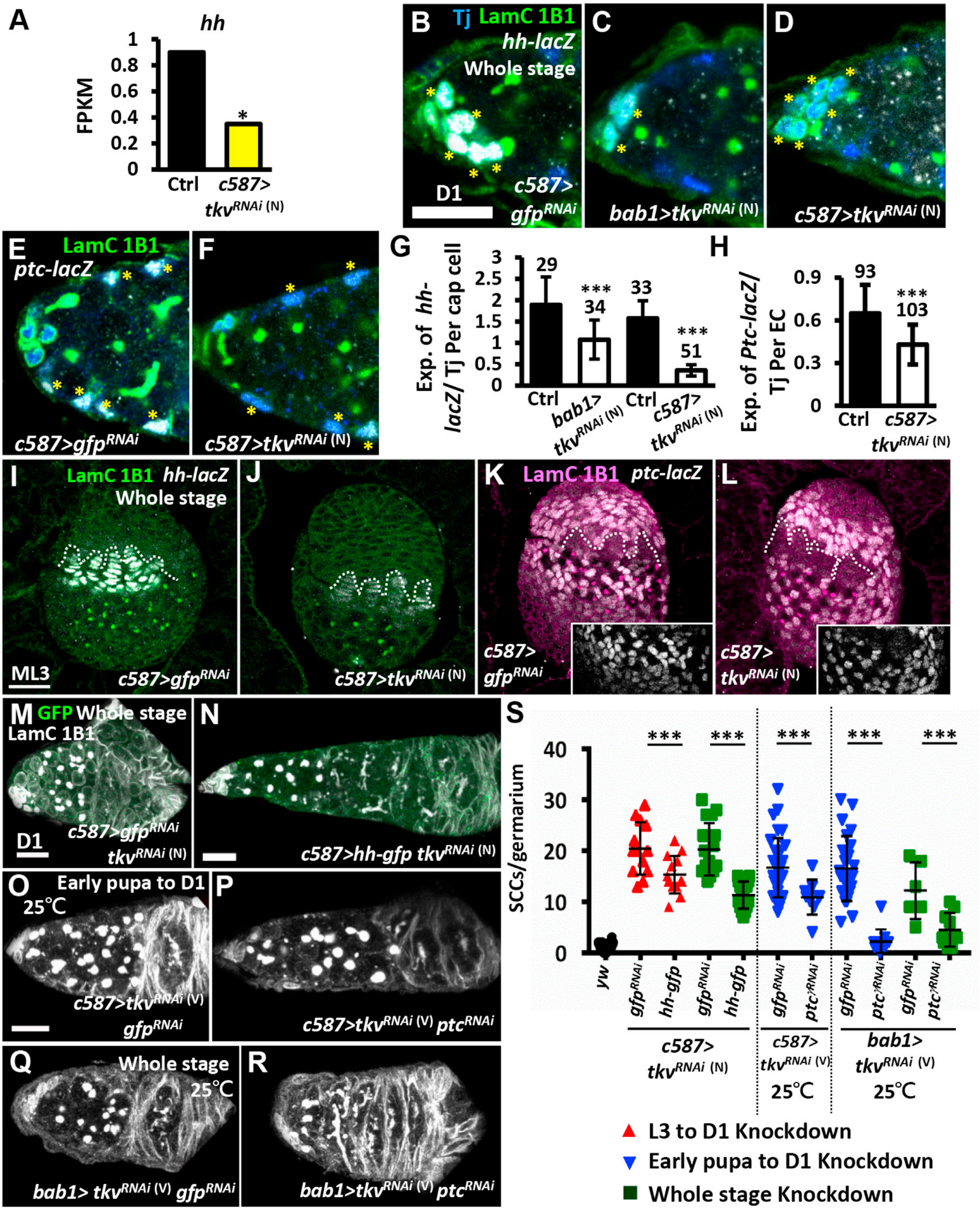

(legend on next page)

results demonstrate that Tkv signaling in the ovarian soma promotes Egfr signaling to suppress Dally expression and thereby shape the localization of Dpp signals to limit GSC number.

Somatic Tkv Signaling Promotes Germ Cell Differentiation via Hh Signaling

Knockdown of *tkv* using *bab1-GAL4* results in SCC accumulation but does not alter Egfr signaling, suggesting that additional Tkv-downstream effectors independently control germ cell differentiation. By analyzing RNA-seq results, we found that transcripts of *hh* were significantly reduced in *c587>tkv*$^{RNAi\ (N)}$ germaria (Figure 5A), reminiscent of a study that showed Hh signaling functions in ECs for germ cell differentiation (Lu et al., 2015). We verified that Hh signaling was reduced using *hh-lacZ*, a transcriptional reporter (Forbes et al., 1996; Lai et al., 2017), and *ptc-lacZ*, an Hh signaling reporter (Chen and Struhl, 1996; Lai et al., 2017). Results showed that *hh-lacZ* was significantly decreased in niche cap cells of somatic *tkvKD* germaria at day 1 (Figures 5B–5D and 5G). Consistently, *ptc-lacZ* was also dramatically reduced in ECs of somatic *tkvKD* germaria (Figures 5E, 5F, and 5H). Reductions of *hh-lacZ* and *ptc-lacZ* expression were also observed in TFs and ICs, respectively, of mid-L3 gonads from somatic *tkvKD* larvae (Figures 5I–5L). The reduction of Hh signaling was not due to decreased canonical Dpp signaling in the soma, as *ptc-lacZ* expression levels in ECs were comparable in control and the germaria with somatic *mad*-knockdown from the L3 to adult stages (Figures S6A and S6B). Disruption of Hh signaling from L3 to adult stages, by knockdown of *hh* or *smoothened* (*smo*, the Hh receptor) in the soma, also caused SCC accumulation (Figures S6C–S6L). These results suggest the involvement of Hh signaling in the developing soma for germ cell differentiation.

It has been reported that Hh signaling suppresses *dpp* transcription in adult ECs and promotes EC membrane extension to force germ cell differentiation (Huang et al., 2017; Lu et al., 2015). We observed defective membrane extension by ECs (derived from ICs) in germaria of newly eclosed flies (Figures S6M–S6O). However, we did not see increased *dpp* transcripts in ECs; instead, *dpp* transcript levels were reduced in cap cells of *bab1>tkv*$^{RNAi\ (N)}$ ovaries (see Figures S3G and S3H), which was in agreement with studies that show that Hh signaling regulates *dpp* transcription in the wing disc (Aza-Blanc et al., 1997).

We directly tested the role of Hh signaling in this process by supplying Hh to somatic *tkvKD* gonads and subsequently examining SCC number in 1-day-old germaria. We found that expression of Hh-GFP fusion protein in the *tkvKD* gonads either from L3 to adult stages, or throughout development, significantly reduced SCC accumulation compared with germaria with *tkvKD* alone (Figures 5M, 5N, and 5S). Forcing Hh signaling in *c587>tkv*$^{RNAi\ (N)}$ gonads by knockdown of *ptc* (encodes a Smo suppressor) from early pupal to adult stages, or during all developmental stages, also partially suppressed SCC accumulation (Figures 5O, 5P, and 5S). Notably, neither overexpression of Egfr nor activation of Hh signaling in *c587>tkv*^{RNAi} gonads could completely suppress SCC accumulation (see also Figure 4), implying that both Hh and Egfr signaling are required downstream of Tkv for germ cell differentiation. Some germaria with *bab1-GAL4*-driven *ptc* and *tkv* co-knockdown from early pupal to adult stages, or throughout all developmental stages, exhibited SCC numbers that were comparable with those of *gfpKD* controls (Figures 5Q–5S). This result implies that defective Hh signaling is primarily responsible for SCC accumulation in *bab1>tkv*$^{RNAi\ (V)}$ ovaries, wherein Egfr signaling was not affected. We also noticed that the blunted EC membrane protrusions in somatic *bab1>tkv*$^{RNAi\ (V)}$ germaria were

Figure 5. Dpp Signaling in the Soma Limits GSC Number via Hh Signaling

(A) RNA-seq-based gene expression values (FPKM) for *hh* in the 1-day-old control and *c587>tkv*$^{RNAi\ (N)}$ germaria. Statistics analysis was from two biological replicates.

(B–F) One-day-old *c587>gfp*^{RNAi} (B and E), *bab1-tkv*$^{RNAi\ (N)}$ (C), and *c587>tkv*$^{RNAi\ (N)}$ germaria (D and F) with staining for 1B1 (green, fusomes), LamC (green, cap cell nuclear envelopes), Tj (blue, ECs), *hh-lacZ* in (B–D) (gray), and *ptc-lacZ* in (E and F) (gray). Asterisks in (B)–(D) indicate cap cells, and in (E) and (F) indicate ECs.

(G) Ratio of *hh-lacZ* to Tj in cap cells of control, *bab1-tkv*$^{RNAi\ (N)}$, and *c587>tkv*$^{RNAi\ (N)}$ germaria.

(H) Ratio of *ptc-lacZ* to Tj in ECs of control and *c587>tkv*$^{RNAi\ (N)}$ germaria. Number of cells analyzed are shown above each bar.

(I–L) ML3 *c587>gfp*^{RNAi} (I and K) and *c587>tkv*$^{RNAi\ (N)}$ gonads (J and L) with 1B1 and LamC, green in (I and J), magenta in (K and J), *hh-lacZ* (gray) in (I) and (J), and *ptc-lacZ* (gray) in (K) and (L). Dashed line indicates TFts; inserts show intermingled cell regions with only the *ptc-lacZ* channel.

(M–R) One-day-old germaria expressing *gfp*^{RNAi} *tkv*$^{RNAi\ (N)}$ (M), *hh-gfp tkv*$^{RNAi\ (N)}$ (N), *tkv*$^{RNAi\ (V)}$*gfp*^{RNAi} (O and Q), and *tkv*$^{RNAi\ (V)}$*ptc*^{RNAi} (P and R) driven by *c587-GAL4* (M–P) or *bab1-GAL4* (Q and R). Germaria with GFP (green), 1B1, and LamC (gray) are shown.

(S) Number of SCCs per germarium at day 1 of flies with indicated *RNAi* expression from ML3 to D1, early pupa to D1 stages, or throughout development (whole stage). Knockdown experiments were carried out at 29°C, except where otherwise indicated. The genotype of the control in (A) is *UAS-tkv*$^{RNAi\ (N)}$/+. The genotypes of the controls in (E and H) are *bab1-GAL4/+* or *c587-GAL4/+*. Scale bars, 10 μm (B, M, N, and Q) and 20 μm (I). Error bars indicate SE; $^{*}p < 0.01$, $^{***}p < 0.001$.

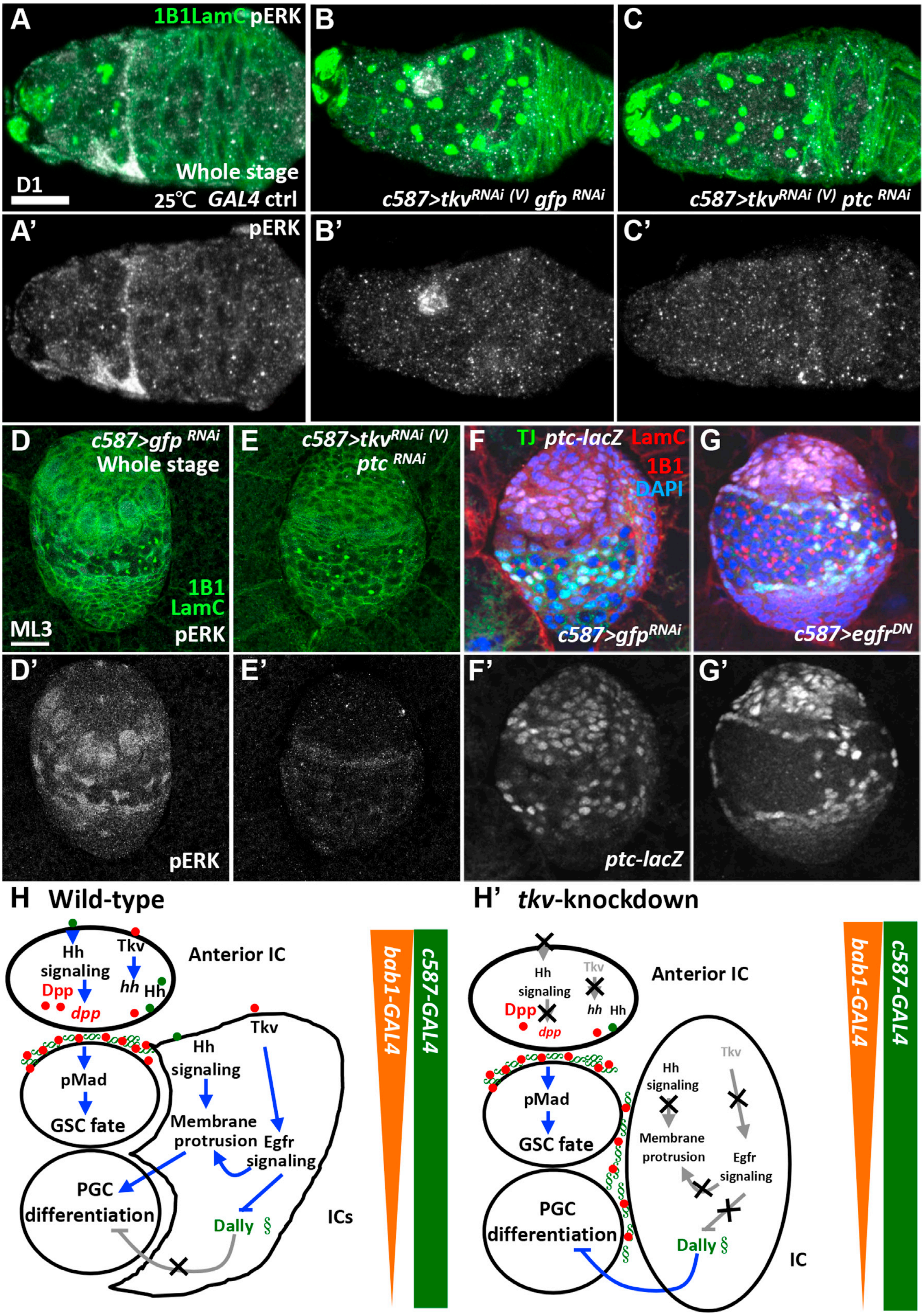

(legend on next page)

rescued by *ptcKD* (Figures 5Q and 5R). Together, these results show that Hh signaling in the soma is controlled by Tkv and promotes membrane extension of ECs for germ cell differentiation. At the same time, Tkv-mediated Hh signaling upregulates somatic *dpp* expression in niche precursors, forming a positive feedback loop to enhance the Dpp signal.

Egfr and Hh Signaling Are Regulated in Parallel by Tkv in the Soma

We next asked if Egfr and Hh signaling crosstalk to control GSC number. Egfr signaling activity, as revealed by pERK staining, was strongly present in ECs of controls (Figure 6A) and dramatically reduced in the somatic *tkvKD* germarium (Figure 6B), but pERK levels were not rescued in germaria with *tkv* and *ptc* co-knockdown throughout developmental stages (Figure 6C). A similar set of observations were made in mid-L3 somatic *tkvKD* gonads with or without *ptcKD* (Figures 6D and 6E). Correspondingly, Hh signaling activity, as revealed by *ptc-lacZ*, was not altered in Egfr signaling-defective gonads (Figures 6F and 6G). Thus, we conclude that Tkv signaling in ICs independently regulates Hh and Egfr signaling to limit GSC number by stimulating differentiation in PGCs that are not selected as GSCs by the niche.

DISCUSSION

A properly sized stem cell pool is critical for maintaining tissue homeostasis, but the mechanisms that regulate the number of stem cells in a niche are not fully understood. Here, we report that non-canonical BMP signaling independently promotes Hh and Egfr signaling in the developing soma to restrict BMP signaling territory for GSC specification. In the developing ovary (Figure 6H), *dpp* mRNA is mainly produced by anterior somatic gonadal cells (Zhu and Xie, 2003), including niche precursors, such as TF cells and anterior ICs. Coincident expression of Dally is highly expressed in the extracellular matrix of niche precursors, and helps to enrich the Dpp signal within the niche (Matsuoka et al., 2013). Dpp signaling is then activated through a canonical Smad-dependent pathway in PGCs that are adjacent to the niche, allowing the cells to adopt a GSC fate. Dpp signal is also received in Tkv-expressing somatic cells, but coordinates with Egfr and Hh signaling to constrain Dpp signals located in the niche. In ICs, Tkv activates Egfr signaling to suppress Dally, limiting the spread of Dpp signals outside of the niche. In addition, Egfr signaling is also known to promote EC cellular protrusion, which contributes to germ cell differentiation (Banisch et al., 2017). On the other hand, Tkv-mediated signaling promotes *hh* transcription in anterior ICs (putative niche precursors [Lai et al., 2017]). This action promotes Hh signaling, which has the dual effects of controlling EC membrane extension to facilitate germ cell differentiation. In anterior ICs, Hh signaling promotes Dpp expression to strengthen Tkv signaling. When Tkv is eliminated from the soma (Figure 6H′), Egfr signaling in ICs is reduced, resulting in the upregulation of Dally and a subsequent loss of control over Dpp distribution. At the same time, Hh signaling is reduced in niche precursors and ICs, which diminishes Dpp expression in the niche precursors and blunts membrane extension in ICs (called ECs only after germarium is formed). These reductions of Egfr and Hh signaling in the developing soma result in the formation of ectopic GSCs in the germarium.

The *Drosophila* Ovarian Soma Utilizes Non-canonical Dpp Signaling to Restrict the Boundary of Niche Activity

Stemness factors must be restricted to the niche during organ development to recruit and maintain an appropriate

Figure 6. Somatic Tkv Controls Hh and Egfr in Parallel to Maintain GSC Number

(A–C) One-day-old control (ctrl) (A), *c587>tkv*$^{RNAi\ (V)}$ *gfp*^{RNAi} (B) and *c587>tkv*$^{RNAi\ (V)}$ *ptc*^{RNAi} germaria (C) with 1B1 (green, fusomes), LamC (green, cap cell nuclear envelopes) and pERK (gray) labeling. Only pERK staining is shown in (A′)–(C′).

(D and E) ML3 *c587>gfp*^{RNAi} (D) and *c587>tkv*$^{RNAi\ (V)}$*ptc*^{RNAi} gonads (E) with 1B1 (green), LamC (green), and pERK (gray) labeling. (D′) and (E′) show pERK channel only.

(F and G) ML3 *c587>gfp*^{RNAi} (F) and *c587>egfr*^{DN}gonads (G) with staining for 1B1 (red), LamC (red), Tj (green, ICs), *ptc-lacZ* (gray), and DAPI (blue, DNA). (F′) and (G′) show *ptc-lacZ* channel only. Knockdown experiments were carried out at 29°C, except where otherwise indicated. Whole stage (throughout development) indicates the time window for transgene expression. The genotype of the control in (A) is *c587-GAL4/+*. Scale bars, 10 μm (A) and 20 μm (D).

(H and H′) Model of somatic Tkv regulation of Hh and Egfr signaling to limit the GSC pool. In the wild-type larval ovary (H), Tkv-mediated signaling elevates *hh* transcripts and activates Hh signaling in anterior of ICs to enhance Dpp expression, which strengthens Tkv-mediated signaling in both GSCs and somatic cells. Hh signaling is also activated in posterior ICs to drive PGC differentiation, at least in part by promoting membrane extension of ICs. Tkv-mediated signaling in ICs also positively regulates Egfr signaling, which diminishes Dally transcripts to broaden Dpp distribution and promotes IC membrane extension. In the somatic *tkvKD* larval ovary (H′), Hh and Egfr signaling is attenuated, and Dally is upregulated, thereby expanding Dpp signal distribution and leading to overpopulation of GSCs. *bab1-GAL4* is strongly expressed in anterior of gonadal somatic cells but gradually restricted in the GSC niche; *c587-GAL4* is expressed in all somatic cells but gradually exclusively expressed in ECs.

number of stem cells. Dpp can act as a long-range morphogen and is produced by the niche at high levels, but the signal is only intended for neighboring GSCs, located up to one-cell diameter from the niche. It has been previously shown that Egfr signaling in ICs diminishes Dpp signaling outside the niche (Matsuoka et al., 2013). In this process, PGCs produce Spitz, which activates Egfr signaling in ICs to suppress expression of Dally. Since Dally coordinates Dpp signals to localize on the cell surface, Dpp signaling is reduced outside of the niche when Dally is suppressed (Guo and Wang, 2009; Matsuoka et al., 2013). Here, we report that Tkv, encoded by the *tkv-A* isoform, induces canonical Dpp signaling in PGCs within the niche to specify a GSC fate, while *tkv-D*-encoded Tkv mediates non-canonical Dpp signaling through Egfr and Hh in the soma to prevent Dpp leaking from the niche. Although we do not know how Tkv affects *hh* transcription, our genetic data suggest that Tkv acts upstream of Egfr, since overexpressing a constitutively active form of Egfr suppressed *tkvKD*-induced SCC accumulation. However, our RNA-seq results revealed that transcript levels of *egfr* and genes encoding Egfr ligand (Spitz) (Tio et al., 1994) and modulators (Argos and Gone early) (Klein et al., 2004; Matsuoka et al., 2014) were not affected in somatic *tkvKD* ovaries. We speculate that non-canonical Dpp signaling may control Egfr at a post-transcriptional level, or it may influence the competence of ICs to respond to the Spitz signal.

Luo et al. (2015) previously reported that Tkv acts as a receptor sink in ECs of the adult germaria (derived from ICs) to sequester excess Dpp that is found outside the niche. Similar to our findings, components of canonical Dpp signaling were reported to be unnecessary in this process. However, there are several key differences that make the two studies complementary, providing a more nuanced understanding of the role of Tkv in defining the niche. First, the timing of *tkv* knockdown was different. Luo et al. suppressed *tkv* expression in ECs at the adult stage, while we manipulated *tkv* expression in the soma during developmental stages. Second, the molecular action of Tkv in limiting GSC number is different. In the previous study, knockdown of *tkv* in adult ECs did not affect Egfr signaling or *dally* expression, and overexpressing the extracellular domain of Tkv significantly reduced SCC number in *tkv*-knockdown germaria. Therefore, the authors concluded that expression of Tkv in adult ECs serves as a sink to sequester Dpp signals that may spread outside of the niche. In contrast, our results showed that Egfr signaling is dramatically reduced, and that *dally* becomes ectopically expressed in ICs/ECs when *tkv* expression is eliminated in the ovarian soma during development. These events were shown to be functionally important, because activation of Egfr signaling or knockdown of *dally* expression in the *tkvKD* ovarian soma during development were sufficient to reduce SCC number. Lastly, we also found that, in the developing soma, Tkv promoted Hh signaling, which may control membrane extension of ECs for germ cell differentiation. Knockdown of *tkv* in the soma of larval ovaries resulted in decreased Hh signaling, and forcing Hh signaling in *tkvKD* somatic cells reduced SCC accumulation. Therefore, we conclude that, during developmental stages and at the adult stage, ovarian somatic cells most likely use different strategies to prevent Dpp leakage from the niche in order to maintain a proper number of GSCs. However, we could not rule out the possibility that Tkv may promote IC or EC proper differentiation, which is required for GSC progeny differentiation. In this case, SCC accumulation in the germaria developed from the somatic *tkvKD* gonad is a consequence of failed differentiation of ICs/or ECs.

Hh and BMP Signaling in the Soma Are Intertwined in Determining GSC Fate

In this study, we have shown that somatic knockdown of *tkv* in developing ovaries results in formation of ectopic GSCs. In addition, somatic *tkvKD* ovaries exhibit a reduction of *hh* transcript level, which may lead to a reduction of *dpp* transcript levels. These results suggest that Hh signaling is sandwiched by upstream and downstream Dpp signaling in the control of GSC number; as such, Dpp signaling activates transcription of *hh* to promote *dpp* transcription, which in turn activates Dpp signaling in the soma. In adult female flies, both promotion and suppression of Dpp have been reported as effects of Hh signaling in ECs (Huang et al., 2017; Lu et al., 2015; Rojas-Rios et al., 2012). In male flies, Hh is mainly produced in the testes by hub cells in the GSC niche (Zhang et al., 2013). This signal is received by adjacent cyst somatic stem cells, which are also in the GSC niche (Amoyel et al., 2013; Zhang et al., 2013), and further enhances transcription of *dpp*, leading to the activation of Dpp signaling in GSCs to maintain an undifferentiated state. However, the role of Hh signaling in somatic gonadal precursors of the developing testis has not been reported.

In mammals, testicular GSCs directly adhere to the Sertoli cells that constitute the GSC niche (Payne et al., 2010). The Sertoli cells express Desert hedgehog (Dhh), which controls spermatogenesis (Bitgood et al., 1996). During development, Dhh signaling specifies the fetal Leydig cell lineage that produces testosterone for masculinization of a male fetus (Svechnikov et al., 2010; Yao et al., 2002). Despite the known role of Dhh, it is not clear if somatic cells regulate GSCs via BMP and Hh signaling pathways in mammalian testes and ovaries. However, a previous report suggests that cancer stem cells in ovarian cancer may be derived from GSCs (Kim et al., 2014), and cancer-associated mesenchymal stem cells (the niche for cancer

stem cells) express a high level of BMP4, which promotes tumor growth by increasing the number of cancer stem cells. The BMP4 signals from cancer-associated mesenchymal stem cells further activate expression of *HH* in cancer stem cells, and that action drives more BMP4 production from the mesenchymal cells, forming a positive feedback loop that confers resistance to chemotherapeutics (Coffman et al., 2016). Together, these studies show that in the niche, complex interactions between BMP and Hh signaling govern GSC and cancer stem cell numbers, and therefore further studies on BMP-Hh regulation may allow us to understand how both GSCs and cancer stem cells are formed, eventually benefiting cancer therapy.

EXPERIMENTAL PROCEDURES

Fly stocks were maintained at 22°C–25°C on standard medium, unless otherwise indicated. *yw* was used as a wild-type control. *hh-lacZ* and *ptc-lacZ* were used to monitor *hh* transcription and Hh signaling activity (Chen and Struhl, 1996; Forbes et al., 1996). *dpp2.0-lacZ* was used to examine transcriptional activity of *dpp* (Luo et al., 2017). *P2-lacZ* consists of a fragment of the *tkv* promoter (2L 5,237,025–5,245,570 base pairs) containing the first exons of *tkv-B*, *-C*, and *–D* transcripts, followed by a *lacZ* reporter. This construct was used to monitor *tkv* expression (Luo et al., 2015). *UAS-RNAi* lines against *tkv* (N no. 14026-R3 and V no. 3059), *punt* (V no. 107071), *mad* (B no. 31315 and V no. 12635), *hh* (N no. 4637-R2), *smo* (B no. 43134), *dally* (N no. 4974-R1), *dpp* (N no. 9885-R2), *ptc* (N no. 2411R1), and *GFP* (B no. 9331 and B no. 9330) were obtained from National Institute of Genetics (N), Vienna Drosophila Resource Center (V), or Bloomington Drosophila Stock Center (B). The efficiency of the dpp^{RNAi} line was examined in this study (see Figures S4I and S4J), and the efficiencies of other *RNAi* lines have been reported previously (Figeac et al., 2010; Lai et al., 2017; Lesokhin et al., 1999; Okano et al., 1994). *UASp-mCD8-gfp*, *UAS-hh-GFP*, $UAS\text{-}tkv^{CA}$ (B no. 36537), $UAS\text{-}egfr^{DN}$ (B no. 5364), $UAS\text{-}egfr^{A887T}$ (B no. 9533), and $UAS\text{-}egfr^{\lambda Top\ 3.1}$ (a gift from Dr. Henry Sun, Academia Sinica, Taiwan) have been described previously (Bolivar et al., 2006; Guo and Wang, 2009; Kao et al., 2015; Lai et al., 2017; Li et al., 2003; Queenan et al., 1997; Torroja et al., 2004; Zhu and Xie, 2003). Flies expressing RNAi or other transgenes driven by *bab1-GAL4* or *c587-GAL4* also carried $tub\text{-}GAL80^{ts}$ to control GAL4 expression; those flies were cultured at 18°C to silence GAL4 expression and were cultured at 29°C to allow GAL4 expression (McGuire et al., 2004). Other genetic tools are described in flybase (http://flybase.org).

Other detail experimental procedures are shown in Supplemental Information.

SUPPLEMENTAL INFORMATION

Supplemental Information includes Supplemental Experimental Procedures and six figures and can be found with this article online at https://doi.org/10.1016/j.stemcr.2018.07.008.

AUTHOR CONTRIBUTIONS

C.-Y.T., H.J.H., and Y.-H.S. conceived and designed the experiments. C.-M.L., C.-Y.T., Y. Cho, S.-M.Y, K.-Y.L., and Y.-H.S. performed the experiments. Y. Cai provided fly lines and reagents, discussed and interpreted results, and troubleshot experiments. C.-Y.T. and H.-J.H. analyzed the data and wrote the manuscript. O.A., K.-Y.L., and E.R. finalized the revised experiments.

ACKNOWLEDGMENTS

We thank D. Godt, E.C. Lai, and the Developmental Studies Hybridoma Bank for antibodies and the Bloomington Stock Center, the Vienna *Drosophila* RNAi Center, and the National Institute of Genetics for *Drosophila* stocks. We also thank Dr. M. Calkins for English editing. This work was supported by intramural funding from the Institute of Cellular and Organismic Biology, Academia Sinica, Taiwan (grant 022347), an Academia Sinica thematic grant (grant 034003), and the Ministry of Science and Technology, Taiwan (grant 104-2311-B-001-029-MY3).

Received: February 3, 2018
Revised: July 18, 2018
Accepted: July 19, 2018
Published: August 16, 2018

REFERENCES

Amoyel, M., Sanny, J., Burel, M., and Bach, E.A. (2013). Hedgehog is required for CySC self-renewal but does not contribute to the GSC niche in the testis. Development *140*, 56.

Aza-Blanc, P., Ramirez-Weber, F.A., Laget, M.P., Schwartz, C., and Kornberg, T.B. (1997). Proteolysis that is inhibited by hedgehog targets Cubitus interruptus protein to the nucleus and converts it to a repressor. Cell *89*, 1043–1053.

Banisch, T.U., Maimon, I., Dadosh, T., and Gilboa, L. (2017). Escort cells generate a dynamic compartment for germline stem cell differentiation via combined Stat and Erk signalling. Development *144*, 1937.

Bitgood, M.J., Shen, L., and McMahon, A.P. (1996). Sertoli cell signaling by Desert hedgehog regulates the male germline. Curr. Biol. *6*, 298–304.

Bolivar, J., Pearson, J., Lopez-Onieva, L., and Gonzalez-Reyes, A. (2006). Genetic dissection of a stem cell niche: the case of the *Drosophila* ovary. Dev. Dyn. *235*, 2969–2979.

Chen, D., and McKearin, D.M. (2003). A discrete transcriptional silencer in the bam gene determines asymmetric division of the *Drosophila* germline stem cell. Development *130*, 1159.

Chen, Y., and Struhl, G. (1996). Dual roles for patched in sequestering and transducing Hedgehog. Cell *87*, 553–563.

Coffman, L.G., Choi, Y.J., McLean, K., Allen, B.L., di Magliano, M.P., and Buckanovich, R.J. (2016). Human carcinoma-associated mesenchymal stem cells promote ovarian cancer chemotherapy resistance via a BMP4/HH signaling loop. Oncotarget *7*, 6916–6932.

Figeac, N., Jagla, T., Aradhya, R., Da Ponte, J.P., and Jagla, K. (2010). *Drosophila* adult muscle precursors form a network of interconnected

cells and are specified by the rhomboid-triggered EGF pathway. Development *137*, 1965–1973.

Forbes, A.J., Lin, H., Ingham, P.W., and Spradling, A.C. (1996). Hedgehog is required for the proliferation and specification of ovarian somatic cells prior to egg chamber formation in *Drosophila*. Development *122*, 1125.

Fuller, M.T., and Spradling, A.C. (2007). Male and female *Drosophila* germline stem cells: two versions of immortality. Science *316*, 402–404.

Gabay, L., Seger, R., and Shilo, B.-Z. (1997). In situ activation pattern of drosophila EGF receptor pathway during development. Science *277*, 1103.

Gilboa, L., and Lehmann, R. (2006). Soma-germline interactions coordinate homeostasis and growth in the *Drosophila* gonad. Nature *443*, 97–100.

Guo, Z., and Wang, Z. (2009). The glypican Dally is required in the niche for the maintenance of germline stem cells and short-range BMP signaling in the *Drosophila* ovary. Development *136*, 3627–3635.

Hamaratoglu, F., Affolter, M., and Pyrowolakis, G. (2014). Dpp/BMP signaling in flies: from molecules to biology. Semin. Cell Dev. Biol. *32*, 128–136.

Harris, R.E., and Ashe, H.L. (2011). Cease and desist: modulating short-range Dpp signalling in the stem-cell niche. EMBO Rep. *12*, 519–526.

Hsu, H.J., and Drummond-Barbosa, D. (2011). Insulin signals control the competence of the *Drosophila* female germline stem cell niche to respond to notch ligands. Dev. Biol. *350*, 290–300.

Huang, J., Reilein, A., and Kalderon, D. (2017). Yorkie and Hedgehog independently restrict BMP production in escort cells to permit germline differentiation in the ovary. Development *144*, 2584.

König, A., and Shcherbata, H.R. (2015). Soma influences GSC progeny differentiation via the cell adhesion-mediated steroid-let-7-Wingless signaling cascade that regulates chromatin dynamics. Biol. Open *4*, 285–300.

Kao, S.H., Tseng, C.Y., Wan, C.L., Su, Y.H., Hsieh, C.C., Pi, H., and Hsu, H.J. (2015). Aging and insulin signaling differentially control normal and tumorous germline stem cells. Aging Cell *14*, 25–34.

Kim, K.H., Kang, Y.J., Jo, J.O., Ock, M.S., Moon, S.H., Suh, D.S., Yoon, M.S., Park, E.S., Jeong, N., Eo, W.K., et al. (2014). DDX4 (DEAD box polypeptide 4) colocalizes with cancer stem cell marker CD133 in ovarian cancers. Biochem. Biophys. Res. Commun. *447*, 315–322.

Kirilly, D., Wang, S., and Xie, T. (2011). Self-maintained escort cells form a germline stem cell differentiation niche. Development *138*, 5087–5097.

Kirilly, D., and Xie, T. (2007). The *Drosophila* ovary: an active stem cell community. Cell Res. *17*, 15–25.

Klein, D.E., Nappi, V.M., Reeves, G.T., Shvartsman, S.Y., and Lemmon, M.A. (2004). Argos inhibits epidermal growth factor receptor signalling by ligand sequestration. Nature *430*, 1040.

Lai, C.M., Lin, K.Y., Kao, S.H., Chen, Y.N., Huang, F., and Hsu, H.J. (2017). Hedgehog signaling establishes precursors for germline stem cell niches by regulating cell adhesion. J. Cell Biol. *216*, 1439–1453.

Lesokhin, A.M., Yu, S.-Y., Katz, J., and Baker, N.E. (1999). Several levels of EGF receptor signaling during photoreceptor specification in wild-type, ellipse, and null mutant *Drosophila*. Dev. Biol. *205*, 129–144.

Li, L., and Xie, T. (2005). Stem cell niche: structure and function. Annu. Rev. Cell Dev. Biol. *21*, 605–631.

Li, M.A., Alls, J.D., Avancini, R.M., Koo, K., and Godt, D. (2003). The large Maf factor Traffic Jam controls gonad morphogenesis in *Drosophila*. Nat. Cell Biol. *5*, 994–1000.

Liu, M., Lim, T.M., and Cai, Y. (2010). The *Drosophila* female germline stem cell lineage acts to spatially restrict DPP function within the niche. Sci. Signal. *3*, ra57.

Liu, Z., Zhong, G., Chai, P.C., Luo, L., Liu, S., Yang, Y., Baeg, G.-H., and Cai, Y. (2015). Coordinated niche-associated signals promote germline homeostasis in the *Drosophila* ovary. J. Cell Biol. *211*, 469–484.

Lu, T., Wang, S., Gao, Y., Mao, Y., Yang, Z., Liu, L., Song, X., Ni, J., and Xie, T. (2015). COP9-Hedgehog axis regulates the function of the germline stem cell progeny differentiation niche in the *Drosophila* ovary. Development *142*, 4242–4252.

Luo, L., Siah, C.K., and Cai, Y. (2017). Engrailed acts with Nejire to control decapentaplegic expression in the *Drosophila* ovarian stem cell niche. Development *144*, 3224.

Luo, L., Wang, H., Fan, C., Liu, S., and Cai, Y. (2015). Wnt ligands regulate Tkv expression to constrain Dpp activity in the *Drosophila* ovarian stem cell niche. J. Cell Biol. *209*, 595–608.

Margolis, J., and Spradling, A. (1995). Identification and behavior of epithelial stem cells in the *Drosophila* ovary. Development *121*, 3797–3807.

Matsuoka, S., Gupta, S., Suzuki, E., Hiromi, Y., and Asaoka, M. (2014). Gone early, a novel germline factor, ensures the proper size of the stem cell precursor pool in the *Drosophila* ovary. PLoS One *9*, e113423.

Matsuoka, S., Hiromi, Y., and Asaoka, M. (2013). Egfr signaling controls the size of the stem cell precursor pool in the *Drosophila* ovary. Mech. Dev. *130*, 241–253.

McGuire, S.E., Mao, Z., and Davis, R.L. (2004). Spatiotemporal gene expression targeting with the TARGET and gene-switch systems in *Drosophila*. Sci. STKE *2004*, pl6.

Moore, L.A., Broihier, H.T., Van Doren, M., Lunsford, L.B., and Lehmann, R. (1998). Identification of genes controlling germ cell migration and embryonic gonad formation in *Drosophila*. Development *125*, 667–678.

Morris, L.X., and Spradling, A.C. (2011). Long-term live imaging provides new insight into stem cell regulation and germline-soma coordination in the *Drosophila* ovary. Development *138*, 2207–2215.

Morrison, S.J., and Spradling, A.C. (2008). Stem cells and niches: mechanisms that promote stem cell maintenance throughout life. Cell *132*, 598–611.

Okano, H., Yoshikawa, S., Suzuki, A., Ueno, N., Kaizu, M., Okabe, M., Takahashi, T., Matsumoto, M., Sawamoto, K., and Mikoshiba, K. (1994). Cloning of a *Drosophila melanogaster* homologue of the mouse type-I bone morphogenetic proteins-2/-4 receptor: a potential decapentaplegic receptor. Gene *148*, 203–209.

Payne, C.J., Gallagher, S.J., Foreman, O., Dannenberg, J.-H., DePinho, R.A., and Braun, R.E. (2010). Sin3a is required by Sertoli cells to establish a niche for undifferentiated spermatogonia, germ cell tumors, and spermatid elongation. Stem Cells *28*, 1424–1434.

Queenan, A.M., Ghabrial, A., and Schupbach, T. (1997). Ectopic activation of torpedo/Egfr, a *Drosophila* receptor tyrosine kinase, dorsalizes both the eggshell and the embryo. Development *124*, 3871.

Rojas-Rios, P., Guerrero, I., and Gonzalez-Reyes, A. (2012). Cytoneme-mediated delivery of hedgehog regulates the expression of bone morphogenetic proteins to maintain germline stem cells in *Drosophila*. PLoS Biol. *10*, e1001298.

Song, X., Call, G.B., Kirilly, D., and Xie, T. (2007). Notch signaling controls germline stem cell niche formation in the *Drosophila* ovary. Development *134*, 1071–1080.

Song, X., Zhu, C.H., Doan, C., and Xie, T. (2002). Germline stem cells anchored by adherens junctions in the *Drosophila* ovary niches. Science *296*, 1855–1857.

Svechnikov, K., Landreh, L., Weisser, J., Izzo, G., Colón, E., Svechnikova, I., and Söder, O. (2010). Origin, development and regulation of human Leydig cells. Horm. Res. Paediatr. *73*, 93–101.

Tio, M., Ma, C., and Moses, K. (1994). spitz, a *Drosophila* homolog of transforming growth factor-α, is required in the founding photoreceptor cells of the compound eye facets. Mech. Dev. *48*, 13–23.

Torroja, C., Gorfinkiel, N., and Guerrero, I. (2004). Patched controls the Hedgehog gradient by endocytosis in a dynamin-dependent manner, but this internalization does not play a major role in signal transduction. Development *131*, 2395–2408.

Wang, R.N., Green, J., Wang, Z., Deng, Y., Qiao, M., Peabody, M., Zhang, Q., Ye, J., Yan, Z., Denduluri, S., et al. (2014). Bone morphogenetic protein (BMP) signaling in development and human diseases. Genes Dis. *1*, 87–105.

Yao, H.H.-C., Whoriskey, W., and Capel, B. (2002). Desert Hedgehog/Patched 1 signaling specifies fetal Leydig cell fate in testis organogenesis. Genes Dev. *16*, 1433–1440.

Zhang, Z., Lv, X., Jiang, J., Zhang, L., and Zhao, Y. (2013). Dual roles of Hh signaling in the regulation of somatic stem cell self-renewal and germline stem cell maintenance in *Drosophila* testis. Cell Res. *23*, 573–576.

Zhu, C.-H., and Xie, T. (2003). Clonal expansion of ovarian germline stem cells during niche formation in *Drosophila*. Development *130*, 2579–2588.

Stem Cell Reports, Volume *11*

Supplemental Information

Smad-Independent BMP Signaling in Somatic Cells Limits the Size of the Germline Stem Cell Pool

Chen-Yuan Tseng, Yu-Han Su, Shun-Min Yang, Kun-Yang Lin, Chun-Ming Lai, Elham Rastegari, Oyundari Amartuvshin, Yueh Cho, Yu Cai, and Hwei-Jan Hsu

Supplementary information

Four *tkv* transcripts share identical coding region sequences.

Black squares mark the translational start codon of *tkv* transcripts.

```
                 10        20        30        40        50        60        70        80        90       100
        ....|....|....|....|....|....|....|....|....|....|....|....|....|....|....|....|....|....|....|....|
tkv A   ---------------------------------------------------------------------------------------------------- 1
tkv B   ---------------------------------------------------------------------------------------------------- 1
tkv C   ---------------------------------------------------------------------------------------------------- 1
tkv D   TTGCACATTCGTCGATACGTTCTTCGCGAGTCGTGACGTATCCACCGGATTGGAAAAATAAACCATATTTTGAGATACAATCCAAAGAATCGTCGATTTT 100

                110       120       130       140       150       160       170       180       190       200
        ....|....|....|....|....|....|....|....|....|....|....|....|....|....|....|....|....|....|....|....|
tkv A   ---------------------------------------------------------------------------------------------------- 1
tkv B   ---------------------------------------------------------------------------------------------------- 1
tkv C   ---------------------------------------------------------------------------------------------------- 1
tkv D   GTTTACGCGTGAAATATTTTCGAGTTTCAACCAAAAACCTGTGTGCCTAAGCTGAAATATTGTTAACTAATGAAAAACGAATAATAAAAGTGCATCAACG 200

                210       220       230       240       250       260       270       280       290       300
        ....|....|....|....|....|....|....|....|....|....|....|....|....|....|....|....|....|....|....|....|
tkv A   ---------------------------------------------------------------------------------------------------- 1
tkv B   ---------------------------------------------------------------------------------------------------- 1
tkv C   ---------------------------------------------------------------------------------------------------- 1
tkv D   GCAATCTATAGTTAATGATTTAAATGGGAAATTGCACATATATTTCTATGCTTAAACAAGTGCCAGTGCGAATAAACAAAATAGTGTAAACACTGGGGAA 300

                310       320       330       340       350       360       370       380       390       400
        ....|....|....|....|....|....|....|....|....|....|....|....|....|....|....|....|....|....|....|....|
tkv A   ---------------------------------------------------------------------------------------------------- 1
tkv B   ---------------------------------------------------------------------------------------------------- 1
tkv C   ---------------------------------------------------------------------------------------------------- 1
tkv D   AAACAACCAGAAACACACAGGATATGCCTTAACTGCGGATTGAGTTTCACCTGATTTGTATTGCCACCTTTCGTATATAATTATAATCAACGTGATCACG 400

                410       420       430       440       450       460       470       480       490       500
        ....|....|....|....|....|....|....|....|....|....|....|....|....|....|....|....|....|....|....|....|
tkv A   ---------------------------------------------------------------------------------------------------- 1
tkv B   ----------------------------------TGTATAGCGGTTTC-CCATAATTTACACCACTCATGTTACGCTAAAGTTTT-ATTGAGTTCCATTA 64
tkv C   ---------------------------------------------------------------------------------------------------- 1
tkv D   CGCTCACACCAAGCAAAAGCAAAAGCACATCGGCAGCAGAGCACCAACGCCACCACCGCCAGCAACAGCAGCAGGATCAAGTTTTTGGCGAAACCCATAA 500

                510       520       530       540       550       560       570       580       590       600
        ....|....|....|....|....|....|....|....|....|....|....|....|....|....|....|....|....|....|....|....|
tkv A   ---------------------------------------------------------------------------------------------------- 1
tkv B   ATTTACGACTTTCTATGGTCGTAATTAAGATTATCCTTTGCTCGCGCTCCGCAATTGCCGCCGATTTTCCATGAATGGCAGCTTTTTATCGGACCT---- 160
tkv C   ---------------------------------------------------------------------------------------------------- 1
tkv D   TTTCGCTCATCTTCCTGCTCCTCTTCTTCTTCTTTTTCTTCGCGGGCTCCATGGTGTCGCCGGCTTGACCGCCGCCGCCTGCCTATAAACAAACCCAAGA 600

                610       620       630       640       650       660       670       680       690       700
        ....|....|....|....|....|....|....|....|....|....|....|....|....|....|....|....|....|....|....|....|
tkv A   ---------------------------------------------------------------------------------------------------- 1
tkv B   ---GCACTCGCATAATTCATTTTATCATTTTGACAGATTCGGAA----TGGAATAGCGACTTTCCTCGCCCACCCAACACGCGTACGAGATTTCAATTGC 253
tkv C   -----------------------------------------------------------------------ATTTAATGTATGGTTCGTATA--AGGTGC 27
tkv D   GGAGTCCAACCCCAACTCCAGCTCCAACTTCGACTGCGACGGCGACTCCATAACAACGCGAAGACTCGACGAGATACAACGAATCCAGTTCTCCTGGTGC 700

                710       720       730       740       750       760       770       780       790       800
        ....|....|....|....|....|....|....|....|....|....|....|....|....|....|....|....|....|....|....|....|
tkv A   ----------------------------------------------CAGC------TCT--ATCGGGCACACGCTGTTAAAGTTTATTTGTACTGT---- 42
tkv B   -------------------CCGTCGAGTGGCACACATATATTATACCAGC------TCTCGAAATGAGACGCATCATAAATAATAACTAATGTTTTGATA 328
tkv C   -------------------CGTCCTAGTGCTTCT----TGCGACCCTGGC------AGAGAGATTGATGCGCCTT-CAGCTGGTGGCCCTTGAACT--CC 95
tkv D   TTTTGTTTTGCTTTCGTTGCTGCTGGCTGCCTCGCGTTTATTGTTTTGCTGCATAGCCCGGAAAAAATAAACGTGGCAACGAGGGGATATCGAAGGGGAA 800

                810       820       830       840       850       860       870       880       890       900
        ....|....|....|....|....|....|....|....|....|....|....|....|....|....|....|....|....|....|....|....|
tkv A   T--AACGCGAA------TTGGATTAAAATGTTTTGTTTTTTGTTAGTTTTCGGTGTAAATGTGGTTTTAGTGCACTTAACAACTGGTGAATTCAGTGCGA 134
tkv B   A--GACCCCAA------TGAGCGCCAACCACTTTTTCTGTTTATCAAATTACGTATGAA-GTTTGCTCATTAATTTTGGCAA--AGTGAATTCAGTGCGA 417
tkv C   A--GGCCACA-------TCTGGGACAGCCGACGGTCTGTTTGAAGAGATTGCACTAGAG-G------CGCTGAC----GCGG--AGTGAATTCAGTGCGA 173
tkv D   ACTGGCCCCAAAACACCCGGGATCCCGATACCCTCTCTATATAGAGAGGAAGGCCAACAAAAACAACCAAGAACGATCGGTTGGGGCAACAACAACTTAG 900

                910       920       930       940       950       960       970       980       990      1000
        ....|....|....|....|....|....|....|....|....|....|....|....|....|....|....|....|....|....|....|....|
tkv A   ATGTTCAGCCAGACGTCGAAGC--TATGGCCGCCGCCG--CTTTGAGTGGCATGGAAATGGGATCTGGACCAGGATCAGAGGGATATGAGGATGCCGACA 230
tkv B   ATGTTCAGCCAGACGTCGAAGC--TATGGCCGCCGCCG--CTTTGAGTGGCATGGAAATGGGATCTGGACCAGGATCAGAGGGATATGAGGATGCCGACA 513
tkv C   ATGTTCAGCCAGACGTCGAAGC--TATGGCCGCCGCCG--CTTTGAGTGGCATGGAAATGGGATCTGGACCAGGATCAGAGGGATATGAGGATGCCGACA 269
tkv D   TTGCATTGC-AGTTGTTGCCGCCGTATAAAAATCATCAGCCTTTGGCCCACGTCGTTGCTGTTGTTGTTGTTGCTCTTGTTGTAGCCATCAGACATGGCG 999

               1010      1020      1030      1040      1050      1060      1070      1080      1090      1100
        ....|....|....|....|....|....|....|....|....|....|....|....|....|....|....|....|....|....|....|....|
tkv A   ACGAGAAA-AGCAAGACCGTGGAAAATGCCCGCTCCCTAACCTGCTACTGCGATGGCAGTTGTCCGGACAATGTAAGCAATGGAACCTGCGAGACCAGAC 329
tkv B   ACGAGAAA-AGCAAGACCGTGGAAAATGCCCGCTCCCTAACCTGCTACTGCGATGGCAGTTGTCCGGACAATGTAAGCAATGGAACCTGCGAGACCAGAC 612
tkv C   ACGAGAAA-AGCAAGACCGTGGAAAATGCCCGCTCCCTAACCTGCTACTGCGATGGCAGTTGTCCGGACAATGTAAGCAATGGAACCTGCGAGACCAGAC 368
tkv D   CCGAAATCCAGAAAGAAGAAGGCTCATGCCCGCTCCCTAACCTGCTACTGCGATGGCAGTTGTCCGGACAATGTAAGCAATGGAACCTGCGAGACCAGAC 1099

               1110      1120      1130      1140      1150      1160      1170      1180      1190      1200
```

```
       ....|....|....|....|....|....|....|....|....|....|....|....|....|....|....|....|....|....|....|....|
tkv A  CCGGTGGCAGTTGCTTCAGCGCAGTCCAACAGCTTTACGATGAGACGACCGGGATGTACGAGGAGGAGCGTACATATGGATGCATGCCTCCCGAAGACAA 429
tkv B  CCGGTGGCAGTTGCTTCAGCGCAGTCCAACAGCTTTACGATGAGACGACCGGGATGTACGAGGAGGAGCGTACATATGGATGCATGCCTCCCGAAGACAA 712
tkv C  CCGGTGGCAGTTGCTTCAGCGCAGTCCAACAGCTTTACGATGAGACGACCGGGATGTACGAGGAGGAGCGTACATATGGATGCATGCCTCCCGAAGACAA 468
tkv D  CCGGTGGCAGTTGCTTCAGCGCAGTCCAACAGCTTTACGATGAGACGACCGGGATGTACGAGGAGGAGCGTACATATGGATGCATGCCTCCCGAAGACAA 1199

           1210      1220      1230      1240      1250      1260      1270      1280      1290      1300
       ....|....|....|....|....|....|....|....|....|....|....|....|....|....|....|....|....|....|....|....|
tkv A  CGGTGGTTTTCTCATGTGCAAGGTAGCCGCTGTACCCCACCTGCATGGCAAGAACATTGTCTGCTGCGACAAGGAGGACTTCTGCAACCGTGACCTGTAC 529
tkv B  CGGTGGTTTTCTCATGTGCAAGGTAGCCGCTGTACCCCACCTGCATGGCAAGAACATTGTCTGCTGCGACAAGGAGGACTTCTGCAACCGTGACCTGTAC 812
tkv C  CGGTGGTTTTCTCATGTGCAAGGTAGCCGCTGTACCCCACCTGCATGGCAAGAACATTGTCTGCTGCGACAAGGAGGACTTCTGCAACCGTGACCTGTAC 568
tkv D  CGGTGGTTTTCTCATGTGCAAGGTAGCCGCTGTACCCCACCTGCATGGCAAGAACATTGTCTGCTGCGACAAGGAGGACTTCTGCAACCGTGACCTGTAC 1299

           1310      1320      1330      1340      1350      1360      1370      1380      1390      1400
       ....|....|....|....|....|....|....|....|....|....|....|....|....|....|....|....|....|....|....|....|
tkv A  CCCACCTACACACCCAAGCTGACCACACCAGCGCCGGATTTGCCCGTGAGCAGCGAGTCCCTACACACGCTGGCCGTCTTTGGCTCCATCATCATCTCCC 629
tkv B  CCCACCTACACACCCAAGCTGACCACACCAGCGCCGGATTTGCCCGTGAGCAGCGAGTCCCTACACACGCTGGCCGTCTTTGGCTCCATCATCATCTCCC 912
tkv C  CCCACCTACACACCCAAGCTGACCACACCAGCGCCGGATTTGCCCGTGAGCAGCGAGTCCCTACACACGCTGGCCGTCTTTGGCTCCATCATCATCTCCC 668
tkv D  CCCACCTACACACCCAAGCTGACCACACCAGCGCCGGATTTGCCCGTGAGCAGCGAGTCCCTACACACGCTGGCCGTCTTTGGCTCCATCATCATCTCCC 1399

           1410      1420      1430      1440      1450      1460      1470      1480      1490      1500
       ....|....|....|....|....|....|....|....|....|....|....|....|....|....|....|....|....|....|....|....|
tkv A  TGTCCGTGTTTATGCTGATCGTGGCTAGCTTATGTTTCACCTACAAGCGACGCGAGAAGCTGCGCAAGCAGCCACGTCTCATCAACTCAATGTGCAACTC 729
tkv B  TGTCCGTGTTTATGCTGATCGTGGCTAGCTTATGTTTCACCTACAAGCGACGCGAGAAGCTGCGCAAGCAGCCACGTCTCATCAACTCAATGTGCAACTC 1012
tkv C  TGTCCGTGTTTATGCTGATCGTGGCTAGCTTATGTTTCACCTACAAGCGACGCGAGAAGCTGCGCAAGCAGCCACGTCTCATCAACTCAATGTGCAACTC 768
tkv D  TGTCCGTGTTTATGCTGATCGTGGCTAGCTTATGTTTCACCTACAAGCGACGCGAGAAGCTGCGCAAGCAGCCACGTCTCATCAACTCAATGTGCAACTC 1499

           1510      1520      1530      1540      1550      1560      1570      1580      1590      1600
       ....|....|....|....|....|....|....|....|....|....|....|....|....|....|....|....|....|....|....|....|
tkv A  ACAGCTGTCGCCTTTGTCACAACTGGTGGAACAGAGTTCGGGCTCCGGATCGGGATTACCATTGCTGGTGCAAAGAACCATTGCCAAGCAGATTCAGATG 829
tkv B  ACAGCTGTCGCCTTTGTCACAACTGGTGGAACAGAGTTCGGGCTCCGGATCGGGATTACCATTGCTGGTGCAAAGAACCATTGCCAAGCAGATTCAGATG 1112
tkv C  ACAGCTGTCGCCTTTGTCACAACTGGTGGAACAGAGTTCGGGCTCCGGATCGGGATTACCATTGCTGGTGCAAAGAACCATTGCCAAGCAGATTCAGATG 868
tkv D  ACAGCTGTCGCCTTTGTCACAACTGGTGGAACAGAGTTCGGGCTCCGGATCGGGATTACCATTGCTGGTGCAAAGAACCATTGCCAAGCAGATTCAGATG 1599

           1610      1620      1630      1640      1650      1660      1670      1680      1690      1700
       ....|....|....|....|....|....|....|....|....|....|....|....|....|....|....|....|....|....|....|....|
tkv A  GTGCGACTGGTGGGCAAAGGACGATATGGCGAGGTCTGGCTGGCCAAATGGCGCGATGAGCGGGTGGCCGTCAAGACCTTCTTTACGACCGAAGAGGCTT 929
tkv B  GTGCGACTGGTGGGCAAAGGACGATATGGCGAGGTCTGGCTGGCCAAATGGCGCGATGAGCGGGTGGCCGTCAAGACCTTCTTTACGACCGAAGAGGCTT 1212
tkv C  GTGCGACTGGTGGGCAAAGGACGATATGGCGAGGTCTGGCTGGCCAAATGGCGCGATGAGCGGGTGGCCGTCAAGACCTTCTTTACGACCGAAGAGGCTT 968
tkv D  GTGCGACTGGTGGGCAAAGGACGATATGGCGAGGTCTGGCTGGCCAAATGGCGCGATGAGCGGGTGGCCGTCAAGACCTTCTTTACGACCGAAGAGGCTT 1699

           1710      1720      1730      1740      1750      1760      1770      1780      1790      1800
       ....|....|....|....|....|....|....|....|....|....|....|....|....|....|....|....|....|....|....|....|
tkv A  CTTGGTTCCGCGAGACTGAAATCTATCAGACAGTGCTGATGCGACACGACAATATCTTGGGCTTCATTGCCGCCGATATCAAGGGTAATGGTAGCTGGAC 1029
tkv B  CTTGGTTCCGCGAGACTGAAATCTATCAGACAGTGCTGATGCGACACGACAATATCTTGGGCTTCATTGCCGCCGATATCAAGGGTAATGGTAGCTGGAC 1312
tkv C  CTTGGTTCCGCGAGACTGAAATCTATCAGACAGTGCTGATGCGACACGACAATATCTTGGGCTTCATTGCCGCCGATATCAAGGGTAATGGTAGCTGGAC 1068
tkv D  CTTGGTTCCGCGAGACTGAAATCTATCAGACAGTGCTGATGCGACACGACAATATCTTGGGCTTCATTGCCGCCGATATCAAGGGTAATGGTAGCTGGAC 1799

           1810      1820      1830      1840      1850      1860      1870      1880      1890      1900
       ....|....|....|....|....|....|....|....|....|....|....|....|....|....|....|....|....|....|....|....|
tkv A  ACAGATGTTGCTGATCACCGACTACCACGAGATGGGCAGCCTACACGATTACCTCTCAATGTCGGTGATCAATCCGCAGAAGCTGCAATTGCTGGCGTTT 1129
tkv B  ACAGATGTTGCTGATCACCGACTACCACGAGATGGGCAGCCTACACGATTACCTCTCAATGTCGGTGATCAATCCGCAGAAGCTGCAATTGCTGGCGTTT 1412
tkv C  ACAGATGTTGCTGATCACCGACTACCACGAGATGGGCAGCCTACACGATTACCTCTCAATGTCGGTGATCAATCCGCAGAAGCTGCAATTGCTGGCGTTT 1168
tkv D  ACAGATGTTGCTGATCACCGACTACCACGAGATGGGCAGCCTACACGATTACCTCTCAATGTCGGTGATCAATCCGCAGAAGCTGCAATTGCTGGCGTTT 1899

           1910      1920      1930      1940      1950      1960      1970      1980      1990      2000
       ....|....|....|....|....|....|....|....|....|....|....|....|....|....|....|....|....|....|....|....|
tkv A  TCGCTGGCCTCCGGATTGGCCCACCTGCACGACGAGATTTTCGGAACCCCTGGCAAACCAGCTATCGCTCATCGCGATATCAAGAGCAAGAACATTTTGG 1229
tkv B  TCGCTGGCCTCCGGATTGGCCCACCTGCACGACGAGATTTTCGGAACCCCTGGCAAACCAGCTATCGCTCATCGCGATATCAAGAGCAAGAACATTTTGG 1512
tkv C  TCGCTGGCCTCCGGATTGGCCCACCTGCACGACGAGATTTTCGGAACCCCTGGCAAACCAGCTATCGCTCATCGCGATATCAAGAGCAAGAACATTTTGG 1268
tkv D  TCGCTGGCCTCCGGATTGGCCCACCTGCACGACGAGATTTTCGGAACCCCTGGCAAACCAGCTATCGCTCATCGCGATATCAAGAGCAAGAACATTTTGG 1999

           2010      2020      2030      2040      2050      2060      2070      2080      2090      2100
       ....|....|....|....|....|....|....|....|....|....|....|....|....|....|....|....|....|....|....|....|
tkv A  TCAAGCGGAATGGGCAGTGCGCTATTGCTGACTTCGGGCTGGCAGTGAAGTACAACTCGGAACTGGATGTCATTCACATTGCACAGAATCCACGTGTCGG 1329
tkv B  TCAAGCGGAATGGGCAGTGCGCTATTGCTGACTTCGGGCTGGCAGTGAAGTACAACTCGGAACTGGATGTCATTCACATTGCACAGAATCCACGTGTCGG 1612
tkv C  TCAAGCGGAATGGGCAGTGCGCTATTGCTGACTTCGGGCTGGCAGTGAAGTACAACTCGGAACTGGATGTCATTCACATTGCACAGAATCCACGTGTCGG 1368
tkv D  TCAAGCGGAATGGGCAGTGCGCTATTGCTGACTTCGGGCTGGCAGTGAAGTACAACTCGGAACTGGATGTCATTCACATTGCACAGAATCCACGTGTCGG 2099

           2110      2120      2130      2140      2150      2160      2170      2180      2190      2200
       ....|....|....|....|....|....|....|....|....|....|....|....|....|....|....|....|....|....|....|....|
tkv A  CACTCGACGCTACATGGCTCCAGAAGTATTGAGTCAGCAGCTGGATCCCAAGCAGTTTGAAGAGTTCAAACGGGCTGATATGTATTCAGTGGGTCTCGTT 1429
tkv B  CACTCGACGCTACATGGCTCCAGAAGTATTGAGTCAGCAGCTGGATCCCAAGCAGTTTGAAGAGTTCAAACGGGCTGATATGTATTCAGTGGGTCTCGTT 1712
tkv C  CACTCGACGCTACATGGCTCCAGAAGTATTGAGTCAGCAGCTGGATCCCAAGCAGTTTGAAGAGTTCAAACGGGCTGATATGTATTCAGTGGGTCTCGTT 1468
tkv D  CACTCGACGCTACATGGCTCCAGAAGTATTGAGTCAGCAGCTGGATCCCAAGCAGTTTGAAGAGTTCAAACGGGCTGATATGTATTCAGTGGGTCTCGTT 2199

           2210      2220      2230      2240      2250      2260      2270      2280      2290      2300
       ....|....|....|....|....|....|....|....|....|....|....|....|....|....|....|....|....|....|....|....|
tkv A  CTGTGGGAGATGACCCGTCGCTGCTACACACCCGTATCGGGCACCAAGACGACCACCTGCGAGGACTACGCCCTGCCCTATCACGATGTGGTGCCCTCGG 1529
tkv B  CTGTGGGAGATGACCCGTCGCTGCTACACACCCGTATCGGGCACCAAGACGACCACCTGCGAGGACTACGCCCTGCCCTATCACGATGTGGTGCCCTCGG 1812
tkv C  CTGTGGGAGATGACCCGTCGCTGCTACACACCCGTATCGGGCACCAAGACGACCACCTGCGAGGACTACGCCCTGCCCTATCACGATGTGGTGCCCTCGG 1568
tkv D  CTGTGGGAGATGACCCGTCGCTGCTACACACCCGTATCGGGCACCAAGACGACCACCTGCGAGGACTACGCCCTGCCCTATCACGATGTGGTGCCCTCGG 2299

           2310      2320      2330      2340      2350      2360      2370      2380      2390      2400
       ....|....|....|....|....|....|....|....|....|....|....|....|....|....|....|....|....|....|....|....|
tkv A  ATCCCACGTTCGAGGACATGCACGCTGTTGTGTGCGTAAAGGGTTTCCGGCCGCCGATACCATCACGCTGGCAGGAGGATGATGTACTCGCCACCGTATC 1629
```

```
tkv B  ATCCCACGTTCGAGGACATGCACGCTGTTGTGTGCGTAAAGGGTTTCCGGCCGCCGATACCATCACGCTGGCAGGAGGATGATGTACTCGCCACCGTATC 1912
tkv C  ATCCCACGTTCGAGGACATGCACGCTGTTGTGTGCGTAAAGGGTTTCCGGCCGCCGATACCATCACGCTGGCAGGAGGATGATGTACTCGCCACCGTATC 1668
tkv D  ATCCCACGTTCGAGGACATGCACGCTGTTGTGTGCGTAAAGGGTTTCCGGCCGCCGATACCATCACGCTGGCAGGAGGATGATGTACTCGCCACCGTATC 2399

               2410      2420      2430      2440      2450      2460      2470      2480      2490      2500
       ....|....|....|....|....|....|....|....|....|....|....|....|....|....|....|....|....|....|....|....|
tkv A  CAAGATCATGCAGGAGTGCTGGCACCCGAATCCCACCGTTCGGCTGACTGCCCTGCGCGTAAAGAAGACGCTGGGGCGACTGGAAACAGACTGTCTAATC 1729
tkv B  CAAGATCATGCAGGAGTGCTGGCACCCGAATCCCACCGTTCGGCTGACTGCCCTGCGCGTAAAGAAGACGCTGGGGCGACTGGAAACAGACTGTCTAATC 2012
tkv C  CAAGATCATGCAGGAGTGCTGGCACCCGAATCCCACCGTTCGGCTGACTGCCCTGCGCGTAAAGAAGACGCTGGGGCGACTGGAAACAGACTGTCTAATC 1768
tkv D  CAAGATCATGCAGGAGTGCTGGCACCCGAATCCCACCGTTCGGCTGACTGCCCTGCGCGTAAAGAAGACGCTGGGGCGACTGGAAACAGACTGTCTAATC 2499

               2510      2520      2530      2540      2550      2560      2570      2580      2590      2600
       ....|....|....|....|....|....|....|....|....|....|....|....|....|....|....|....|....|....|....|....|
tkv A  GATGTGCCCATTAAGATTGTCTAAGCAGCTGTTTGTAGTCTCGTTTTAGGTTTAACCTAAGTTGCATTTAGTTTTCTGTTTTTGTTCAACTTCTGTTGAA 1829
tkv B  GATGTGCCCATTAAGATTGTCTAAGCAGCTGTTTGTAGTCTCGTTTTAGGTTTAACCTAAGTTGCATTTAGTTTTCTGTTTTTGTTCAACTTCTGTTGAA 2112
tkv C  GATGTGCCCATTAAGATTGTCTAAGCAGCTGTTTGTAGTCTCGTTTTAGGTTTAACCTAAGTTGCATTTAGTTTTCTGTTTTTGTTCAACTTCTGTTGAA 1868
tkv D  GATGTGCCCATTAAGATTGTCTAAGCAGCTGTTTGTAGTCTCGTTTTAGGTTTAACCTAAGTTGCATTTAGTTTTCTGTTTTTGTTCAACTTCTGTTGAA 2599

               2610      2620      2630      2640      2650      2660      2670      2680      2690      2700
       ....|....|....|....|....|....|....|....|....|....|....|....|....|....|....|....|....|....|....|....|
tkv A  TACGCAGTTCCGACCTTTTGTCCGGAAATTCATAGTTAGGTTCTAATCGTAAGTTGCTTGTACGTGAGCGGAGTAAAGCAGAGGATCCTTCAGAGGTGCC 1929
tkv B  TACGCAGTTCCGACCTTTTGTCCGGAAATTCATAGTTAGGTTCTAATCGTAAGTTGCTTGTACGTGAGCGGAGTAAAGCAGAGGATCCTTCAGAGGTGCC 2212
tkv C  TACGCAGTTCCGACCTTTTGTCCGGAAATTCATAGTTAGGTTCTAATCGTAAGTTGCTTGTACGTGAGCGGAGTAAAGCAGAGGATCCTTCAGAGGTGCC 1968
tkv D  TACGCAGTTCCGACCTTTTGTCCGGAAATTCATAGTTAGGTTCTAATCGTAAGTTGCTTGTACGTGAGCGGAGTAAAGCAGAGGATCCTTCAGAGGTGCC 2699

               2710      2720      2730      2740      2750      2760      2770      2780      2790      2800
       ....|....|....|....|....|....|....|....|....|....|....|....|....|....|....|....|....|....|....|....|
tkv A  TATACTACGGTTACTTAAACCGTCGCACTGATTGTAAGCTAGAGTTTAGATATAAGTTGGCAGGAGCTAGTTACTAAGGCCCCTTGCAAAATCTTTGGTT 2029
tkv B  TATACTACGGTTACTTAAACCGTCGCACTGATTGTAAGCTAGAGTTTAGATATAAGTTGGCAGGAGCTAGTTACTAAGGCCCCTTGCAAAATCTTTGGTT 2312
tkv C  TATACTACGGTTACTTAAACCGTCGCACTGATTGTAAGCTAGAGTTTAGATATAAGTTGGCAGGAGCTAGTTACTAAGGCCCCTTGCAAAATCTTTGGTT 2068
tkv D  TATACTACGGTTACTTAAACCGTCGCACTGATTGTAAGCTAGAGTTTAGATATAAGTTGGCAGGAGCTAGTTACTAAGGCCCCTTGCAAAATCTTTGGTT 2799

               2810      2820      2830      2840      2850      2860      2870      2880      2890      2900
       ....|....|....|....|....|....|....|....|....|....|....|....|....|....|....|....|....|....|....|....|
tkv A  AGAAAAATGCTTTAACTATTTGAGAAATATAGGGAGTATTTTGGATGACACCTATTCATGTCGATGTACATAAATATGAGATCAAAACAAAAAATCAATC 2129
tkv B  AGAAAAATGCTTTAACTATTTGAGAAATATAGGGAGTATTTTGGATGACACCTATTCATGTCGATGTACATAAATATGAGATCAAAACAAAAAATCAATC 2412
tkv C  AGAAAAATGCTTTAACTATTTGAGAAATATAGGGAGTATTTTGGATGACACCTATTCATGTCGATGTACATAAATATGAGATCAAAACAAAAAATCAATC 2168
tkv D  AGAAAAATGCTTTAACTATTTGAGAAATATAGGGAGTATTTTGGATGACACCTATTCATGTCGATGTACATAAATATGAGATCAAAACAAAAAATCAATC 2899

               2910      2920      2930      2940      2950      2960      2970      2980      2990      3000
       ....|....|....|....|....|....|....|....|....|....|....|....|....|....|....|....|....|....|....|....|
tkv A  GAAAGAATCGAAATTTATATGTAACATACATTAATTTTTGTGCCAGCTTTTGTACGCTTTAACTAAACGAGTTAAGTCGCCCATTCTTTGACCCCCGAGG 2229
tkv B  GAAAGAATCGAAATTTATATGTAACATACATTAATTTTTGTGCCAGCTTTTGTACGCTTTAACTAAACGAGTTAAGTCGCCCATTCTTTGACCCCCGAGG 2512
tkv C  GAAAGAATCGAAATTTATATGTAACATACATTAATTTTTGTGCCAGCTTTTGTACGCTTTAACTAAACGAGTTAAGTCGCCCATTCTTTGACCCCCGAGG 2268
tkv D  GAAAGAATCGAAATTTATATGTAACATACATTAATTTTTGTGCCAGCTTTTGTACGCTTTAACTAAACGAGTTAAGTCGCCCATTCTTTGACCCCCGAGG 2999

               3010      3020      3030      3040      3050      3060      3070      3080      3090      3100
       ....|....|....|....|....|....|....|....|....|....|....|....|....|....|....|....|....|....|....|....|
tkv A  CTTTAGCCTCAACATAGAACATGTCCTTATATGTTTTGTTTGTATCTAGTGTTTAAGTGTAAACTTTGCAGACTTTGTGCAAGTAAATGCTGCCTTTTGT 2329
tkv B  CTTTAGCCTCAACATAGAACATGTCCTTATATGTTTTGTTTGTATCTAGTGTTTAAGTGTAAACTTTGCAGACTTTGTGCAAGTAAATGCTGCCTTTTGT 2612
tkv C  CTTTAGCCTCAACATAGAACATGTCCTTATATGTTTTGTTTGTATCTAGTGTTTAAGTGTAAACTTTGCAGACTTTGTGCAAGTAAATGCTGCCTTTTGT 2368
tkv D  CTTTAGCCTCAACATAGAACATGTCCTTATATGTTTTGTTTGTATCTAGTGTTTAAGTGTAAACTTTGCAGACTTTGTGCAAGTAAATGCTGCCTTTTGT 3099

               3110      3120      3130      3140      3150      3160      3170      3180      3190      3200
       ....|....|....|....|....|....|....|....|....|....|....|....|....|....|....|....|....|....|....|....|
tkv A  AAAATCCACGGAATTAGTTGCCTTAAGACAAAGTGTAAAGACAGGAGCCAACAGACAGGAGCCCGACTCCCTTCCTCCCCTTTTAGTAGCACATATTTTT 2429
tkv B  AAAATCCACGGAATTAGTTGCCTTAAGACAAAGTGTAAAGACAGGAGCCAACAGACAGGAGCCCGACTCCCTTCCTCCCCTTTTAGTAGCACATATTTTT 2712
tkv C  AAAATCCACGGAATTAGTTGCCTTAAGACAAAGTGTAAAGACAGGAGCCAACAGACAGGAGCCCGACTCCCTTCCTCCCCTTTTAGTAGCACATATTTTT 2468
tkv D  AAAATCCACGGAATTAGTTGCCTTAAGACAAAGTGTAAAGACAGGAGCCAACAGACAGGAGCCCGACTCCCTTCCTCCCCTTTTAGTAGCACATATTTTT 3199

               3210      3220      3230      3240      3250      3260      3270      3280      3290      3300
       ....|....|....|....|....|....|....|....|....|....|....|....|....|....|....|....|....|....|....|....|
tkv A  GTATTTATAACCAGAGATATATAGAACTGGCAGTTGCGTATCTCGAGAAGTGCACTTGTGTCGGCTCTTTTGTTTTGTGTAAATAGCAAAGATGTTTATA 2529
tkv B  GTATTTATAACCAGAGATATATAGAACTGGCAGTTGCGTATCTCGAGAAGTGCACTTGTGTCGGCTCTTTTGTTTTGTGTAAATAGCAAAGATGTTTATA 2812
tkv C  GTATTTATAACCAGAGATATATAGAACTGGCAGTTGCGTATCTCGAGAAGTGCACTTGTGTCGGCTCTTTTGTTTTGTGTAAATAGCAAAGATGTTTATA 2568
tkv D  GTATTTATAACCAGAGATATATAGAACTGGCAGTTGCGTATCTCGAGAAGTGCACTTGTGTCGGCTCTTTTGTTTTGTGTAAATAGCAAAGATGTTTATA 3299

               3310      3320      3330      3340      3350      3360      3370      3380      3390      3400
       ....|....|....|....|....|....|....|....|....|....|....|....|....|....|....|....|....|....|....|....|
tkv A  GGCCGCTAACCATACATATATATAGTATATATATTATATATTATCGTTATAAGCGATTCTGTAAATACTATATATGTATCTTTATCTGTAACCGTTTAAC 2629
tkv B  GGCCGCTAACCATACATATATATAGTATATATATTATATATTATCGTTATAAGCGATTCTGTAAATACTATATATGTATCTTTATCTGTAACCGTTTAAC 2912
tkv C  GGCCGCTAACCATACATATATATAGTATATATATTATATATTATCGTTATAAGCGATTCTGTAAATACTATATATGTATCTTTATCTGTAACCGTTTAAC 2668
tkv D  GGCCGCTAACCATACATATATATAGTATATATATTATATATTATCGTTATAAGCGATTCTGTAAATACTATATATGTATCTTTATCTGTAACCGTTTAAC 3399

               3410      3420      3430      3440      3450      3460      3470      3480      3490      3500
       ....|....|....|....|....|....|....|....|....|....|....|....|....|....|....|....|....|....|....|....|
tkv A  TTATATCTTATCGTTCTTATCATGTAACCTTGTTTAAGCTAGAGTTAGATGACTAGCCTGTAAGAGCAACAAGTAAACTATATGAACTAGATGGATATGT 2729
tkv B  TTATATCTTATCGTTCTTATCATGTAACCTTGTTTAAGCTAGAGTTAGATGACTAGCCTGTAAGAGCAACAAGTAAACTATATGAACTAGATGGATATGT 3012
tkv C  TTATATCTTATCGTTCTTATCATGTAACCTTGTTTAAGCTAGAGTTAGATGACTAGCCTGTAAGAGCAACAAGTAAACTATATGAACTAGATGGATATGT 2768
tkv D  TTATATCTTATCGTTCTTATCATGTAACCTTGTTTAAGCTAGAGTTAGATGACTAGCCTGTAAGAGCAACAAGTAAACTATATGAACTAGATGGATATGT 3499

               3510      3520      3530      3540      3550      3560      3570      3580      3590      3600
       ....|....|....|....|....|....|....|....|....|....|....|....|....|....|....|....|....|....|....|....|
tkv A  ATCTTTCAGTTTTTTTTCTGTTTCATTATTACTGCTCGCAGACCACAAGAACTAAAGTTTTGTATTAAAGCGGTTAGTTAATTTATTTAAAAATAATAAA 2829
tkv B  ATCTTTCAGTTTTTTTTCTGTTTCATTATTACTGCTCGCAGACCACAAGAACTAAAGTTTTGTATTAAAGCGGTTAGTTAATTTATTTAAAAATAATAAA 3112
tkv C  ATCTTTCAGTTTTTTTTCTGTTTCATTATTACTGCTCGCAGACCACAAGAACTAAAGTTTTGTATTAAAGCGGTTAGTTAATTTATTTAAAAATAATAAA 2868
```

```
tkv D  ATCTTTCAGTTTTTTTTCTGTTTCATTATTACTGCTCGCAGACCACAAGAACTAAAGTTTTGTATTAAAGCGGTTAGTTAATTTATTTAAAAATAATAAA 3599

           3610
       ....|....|....|..
tkv A  AAATTATAATTAAACCC 2846
tkv B  AAATTATAATTAAACCC 3129
tkv C  AAATTATAATTAAACCC 2885
tkv D  AAATTATAATTAAACCC 3616
```

Experimental Procedures

Developmental staging of larvae and pupae

The developmental stages of *Drosophila* were morphologically defined as previously described (Ashburner, 2005). Flies were transferred in a fresh vial to lay eggs at 25°C for 6 h and were then removed. The vial was kept at 25°C, and newly hatched larvae were collected as first-instar larvae [~20 h after egg laying (AEL)]. Larvae climbing up and down from food were considered mid-third instar larvae (ML3, ~96 h AEL); at this stage, the GSC niche is forming and recruiting GSCs (Song et al., 2007). Larvae that moved out of food, but for which pupation had not started were considered late-L3 wandering larvae (LL3, ~108 h AEL). When wandering larvae stopped moving, they turning to white pupae were considered to be early pupae (~120 h AEL).

For functional studies of Tkv in the ovarian soma, *tkv* was first knocked down throughout development. To further dissect the developmental timing at which functional Tkv is required in the soma, *tkv* was knocked down either before ML3, after ML3, or after early pupal stage. These stages were easily identified according to the above criteria. Phenotypes were examined in adult germaria of newly eclosed flies in order to avoid any further effects that may occur in adults.

Western blot

Immunobloting was performed as previously described (Tseng et al., 2014). Thirty pairs of LL3 female gonads were dissected and lysed in RIPA buffer (20 mM Tris-HCl pH 7.5, 150 mM NaCl, 1 mM EGTA, 1% NP-40) supplemented with 2X Complete Proteinase Inhibitor Cocktail (Roche) on ice for 1 h. Protein lysates were collected from the supernatant after centrifugation at 4°C; for each sample, 25 μg of lysate was used for analysis. Rabbit anti-phospho-ERK1/2 (Thr202/Thr204) (Cell Signaling Technology, Cat#4370, 1:1000) and rabbit anti-α-tubulin (Sigma T9026, 1:5000) were used as primary antibodies; horseradish peroxidase (HRP)-conjugated goat anti-rabbit IgG (Jackson ImmunoResearch, 1:10,000) was used as secondary antibody. Signals were detected by chemiluminescence with a Western LightningTM Plus-ECL kit (PerkinElmer).

RNA extraction and Quantitative real-time PCR

Total RNA was extracted from 20 pairs of one-day-old ovaries with the GENEzol™ TriRNA Pure kit (Geneaid), according to the manufacturer's manual. Total RNA (1 μg) was reverse transcribed with the Transcriptor First Strand cDNA Synthesis kit (Roche). Steady-state mRNA levels were determined using the LightCycler 480 Probes Master combined with a Universal ProbeLibrary (Roche).

The primer set and probe for each gene were designed from the Roche Universal ProbeLibrary assay design center (https://lifescience.roche.com/global_en.message.html#reference-gene-assays) and are listed below. *tkv*: probe: # 121, 5′-GCCGAAATCCAGAAAGAAGA-3′, 5′-GCAGGTTCCATTGCTTACATT-3′ *dpp*: probe #77, 5′-GTGCGAAGTTTTACACACAAAGA-3′ and 5′-CGCCTTCAGCTTCTCGTC-3′
gbb: probe #59, 5′-CGCTGGAACTCTCGAAATAAA-3′ and 5′-CCACTTGCGATAGCTTCAGA-3′
dally: probe #78, 5′-AGTGGGACTTACAGCGAA
AAAG-3′ and 5′-AGCAGTTGCTCGAACATTCC-3′

RpL19: probe #128, 5′-GAGCGTATTGCCACCAGGA-3′ and 5′- CGATCTCGTCCTCCTTAGCA-3′;

RpL32: probe #117, 5′-CGGATCGATATGCTAAGCTGT-3′ and 5′- CGACGCACTCTGTTGTCG-3′.

RNA sequencing analysis

Forty pairs of ovaries were collected and dissected from one-day-old female flies that were cultured at 29°C throughout all developmental stages. The genotypes of the flies were *c587-GAL4/+; UAS-tkv*$^{RNAi(NIG)}$*/+*, *UAS-tkv*$^{RNAi(NIG)}$*/+; bab1-Gal4/+*, or *UAS-tkv*$^{RNAi(NIG)}$*/+*. Two biological replicate were used in this analysis. Total RNA was extracted by Trizol reagent (Invitrogen) according to the manufacturer's instructions. RNA was quantified by absorbance at 260 nm using a ND-1000 spectrophotometer (Nanodrop Techonology) and quality was assessed using a Bioanalyzer 2100 (Agilent Technology) with a RNA 6000 labchip kit (Agilent Technology). All RNA-seq procedures were carried out according to the manufacturer's protocols from Illumina. Library construction for all samples was accomplished with Agilent's SureSelect Strand Specific RNA library Preparation Kit for 75SE (Paired-End) sequencing on a Solexa platform. The sequence was directly determined using sequencing-by-synthesis technology with a TruSeq SBS kit. Raw sequences were obtained from the Illumina Pipeline software bcl2fastq v2.0 and expected to generate 12.5M (million reads) per sample. The sequences were then filtered to obtain qualified reads. Trimmomatic software was implemented to trim or remove the reads according to the quality score. The gene expression level was calculated as FPKM (Fragment Per Kilobase of transcript per Million mapped reads). For differential expression analysis, CummeRbund was used to perform statistical analysis of gene expression profiles. The reference gene annotations were retrieved from Flybase. Data was deposited in the NCBI GEO under the accession number GSE117251.

Immunohistochemistry and fluorescence microscopy

For immunostaining, gonads and ovaries were dissected, fixed and immunostained at designated stages as described previously (Lai et al., 2017; Tseng et al., 2014). In brief, ovaries were dissected in Grace's insect medium (Lonza) and fixed with 5.3% paraformaldehyde/Grace's insect medium for 13 min with gentle agitation at room temperature. Ovaries were washed in PBST (0.1% Triton X-100 in PBS) 20 min three times, and teased apart in PBST, after which the tissue was incubated with blocking solution (GOAL Bio) for 3 h at room temperature or 4°C overnight. Ovaries were incubated with primary antibodies (diluted in blocking solution) for 3 h at room temperature or 4°C overnight, followed by three or four PBST washes for 30 min each. Then ovaries were incubated with secondary antibodies (diluted in blocking solution) for 3 h at room temperature or 4°C overnight, followed by three to four washes with PBST for 30 min each. The primary antibodies were as follows: mouse anti-Hts (1B1) (Drosophila Studies Hybridoma bank, DSHB, 7H9, 1:50), mouse anti-Lamin (Lam) C (DSHB LC28.26, 1:50), guinea pig anti-Traffic Jam (1:5000; a gift from Dr. Dorothea Godt, University of Toronto, Canada), rabbit anti-Fax (gift from Dr. Eric C. Liebl, 1:1500), mouse anti-β-gal (Promega #Z3783, 1:500), rabbit anti-Vasa (Santa Cruz Sc-30210, 1:500), rabbit anti-GFP (Torry Pines GTX113617, 1:1000), rabbit anti-phospho (p)-ERK (Cell Signaling #4370, 1:200), and rabbit anti-pMad (phospho S423+S425) (Abcam #52903, 1:500). Alexa Fluor 488-, 568- or 633-conjugated goat species-specific secondary antibodies (Molecular Probes, 1:1000) were subsequently used. Samples were stained with 0.5 μg/ml DAPI (Sigma), mounted in 80% glycerol containing 20 μg/mL N-propyl gallate (Sigma), and analyzed with a Zeiss LSM 700 confocal microscope.

GSCs were identified as those cells with a fusome, which was labeled with 1B1 and was juxtaposed to the direct interface between the GSC and the cap cell, which was labeled by LamC (Tseng et al., 2014). Germ cells with round-shaped fusomes and without direct cap cell contacts were counted as SCCs. For measuring ptc-lacZ and hh-lacZ expression, Image J was used to calculate the average fluorescence intensity (arbitrary units) in confocal Z-sections at the largest EC or cap cell nuclear diameter. Statistical analysis was performed using Student's t-test.

***In situ* hybridization**

Fluorescent RNA *in situ* was performed as previously reported (Luo et al., 2015), with slight modifications. In brief, ovaries were dissected in Graces insect medium (Lonza) and fixed with 4% formaldehyde (FA) in PBS-DEPC with 1% DMSO at 4°C overnight. On the second day, ovaries were washed with PBST (0.1% Tween 20 in PBS) for 5 min four times, then dehydrated through a series of ethanol solutions (25%, 50%, 75% and 100%) diluted in PBS for 5 mins in each solution. The dehydrated ovaries were stored at -20°C for at least one overnight period. Ovaries were then rehydrated through a series of ethanol solutions, and treated with proteinase K (50 μg/ml in PBST; Sigma-Aldrich) for 5 min at room temperature. After inactivation of proteinase K by 0.2 M glycine, ovaries were refixed in 4% PFA for 30 min, followed by prehybridization in hybridization solution (50% formamide, 5x SSC, 0.1% Tween-20, 50 μg/μl heparin, 100 μg/ml yeast t-RNA and 10 μg/ml salmon sperm DNA) for 1 h at 60°C. Ovaries were then hybridized with Dig-labeled probes overnight at 60°C. Hybridized tissues were washed with 50% and 25% hybridization solution diluted in 2x SSC, each for 5 mins at 65°C. Then the tissues were rinsed with PBST for 10 min two times, incubated with 3% H_2O_2 in PBST to inactivate endogenous perioxidase (POD), and blocked with 2X blocking buffer in maleic acid (Roche) 1 h at room temperature. Ovaries were incubated with anti-Dig-POD (1: 500, Roche # 11207733910) in blocking buffer at 4°C overnight. After washing with PBST for 10 min six times, signals were developed using a TSA plus fluorescence kit (Perkin Elmer). After washing, ovaries were blocked with blocking solution (GOAL Bio) and then further processed using the immunostaining procedure described above. In order to produce *dally* RNA probes, the 3′ UTR region (~758 bp) of *dally* transcripts was amplified from adult fly ovary cDNA (described above) using a primer set that consisted of 5′-CCAGCCCAAGGGATTGAACA-3′ and 5′-GCATTTCGCATTTCGATTACCC-3′. This fragment was subcloned into a pGEM-T Easy vector (Promega) with two different orientations. Sense and antisense RNA probes labeled with digoxigenin-UTP (Roche) were synthesized from 1 μg of PGEM-T-*dally* plasmids digested with NcoI using the ampliCap^{TM} SP6 high yield message marker kit (Cell Script).

Supplementary Figure legends

Supplementary Fig. 1. Expression of *bab1*-, *c587*- and *hh-Gal4* in the larval gonad and adult germarium. Related to Fig. 1. (A-H) *bab1> gfp* (A, C, E and G) and *c587> gfp* ovaries (B, D, F and H) of late-L3 (LL3) (A and B), 2 hours after pupation (APF) **(**C and D), 48 APF (E and F), and 96 APF (G and H) with signals from GFP (green), Tj (red, ICs), Vasa (blue, germ cells) and LamC in C, G and H (red, nuclear envelopes of TF, cap and stalk cells). **(I and J)** One-day (D)-old *bab1>mCD8-gfp* (I) and c*587> mCD8-gfp* germaria (J) with signals from GFP (green), LamC (red) and 1B1 (red, fusomes). **(K and L)** The mid-L3 (ML3) ovary (K) and one-day-old *hh>mCD8-gfp* germarium (L) with GFP (green), DAPI (blue, DAPI), LamC in L (red), and 1B1 in L (red, fusomes) and Tj in L (gray, escort cell, EC). I', J' and L' only show the mCD8-GFP channel. Scale bar in A-F, 20μm. Scale bar in H-J, 10μm.

Supplementary Fig. 2. Knockdown of somatic Tkv does not affect th number of GSCs, niche cap cells, and egg chamber morphology. Related to Fig. 1. (A and B) Number of cap cells (CpCs) **(A)** and GSCs **(B)** in one-day-old *bab1-GAL4* control (ctrl), *UAS* control, *bab-1>tkv^{RNAi}* $^{(N)}$, and *c587>tkv^{RNAi}* $^{(N)}$ germaria. RNAi was expressed throughout developmental stages at 29°C. The number of analyzed germaria is shown above each bar. The genotype of *bab1-GAL4* control is *bab1-GAL4/+*, and *UAS*

control is *UAS-tkv*$^{RNAi\ (N)}$/+. **(C and D)** One-week-old control (ctrl) (C) and *bab1>tkv*^{RNAi} (D) ovarioles were labeled with LamC (gray, TF and cap cell nuclear envelopes) and 1B1 (gray, fusomes and follicle cell membrane). The control ovariole typically contains a string of five to seven progressively older egg chambers, with the youngest cysts in the apical germarial region and more mature chambers near the oviduct. Knockdown of *tkv* causes spectrosome-containing cell accumulation in the germarium, and fewer egg chambers with normal morphology. These *tkvKD* egg chambers were directly followed by one or two nearly mature eggs, implying that these egg chambers and eggs represent escaped germ cells that have undergone differentiation. However, it is not clear why *bab1>tkv*$^{RNAi\ (N)}$ eggs are not laid. One possibility is that *bab1-GAL4* is also is also expressed in the fat body, which controls vitellogenesis (Yan and Postlethwait, 1990), or the ovarian muscle, which controls ovary contraction for egg laying (Ritsick et al., 2007). *RNAi* was expressed throughout developmental stages at 29°C. The genotype in A is *bab1-GAL4*/+; scale bar, 50 μm.

Supplementary Fig. 3. *dpp* transcripts are not increased in *tkvKD* ovaries, *dpp*^{RNAi} efficiency, and somatic Dpp and Tkv are in the same pathway to limit Bmp signals in the niche. Related to Fig. 3. (A) RNA-seq-based gene expression values (FPKM) and **(B)** qRT-PCR analysis yeild fold changes of *dpp* mRNA in one-day (D)-old control (ctrl) and *c587>tkv*^{RNAi} germaria. Statistics analysis in A is from two biological replicate, and the error bar in B denotes standard error from three independent experiments. **(C and D)** Late-L3 (LL3) *c587>gfp*^{RNAi} and *c587>tkv*$^{RNAi\ (N)}$ gonads with *dpp 2.0-lacZ* (green), Tj (red, ICs) and DAPI (blue, DNA). Dashed lines mark terminal filaments. C' and D' only show *dpp 2.0-lacZ* (gray) channel. **(E and F)** One-day-old *c587>gfp*^{RNAi} and *c587>tkv*$^{RNAi\ (N)}$ germaria with *dpp 2.0-lacZ* (gray), Tj (blue, ICs), 1B1 (red, fusomes) and LamC (red, TF and cap cell nuclear envelopes). Inserts show the indicated region of germaria with only the *dpp 2.0-lacZ* (gray) channel. **(G and H)** *dpp 2.0-lacZ* expression in cap cells of one-day-old *bab-1>gfp*^{RNAi} and *bab-1>tkv*$^{RNAi\ (N)}$ germaria. G' and H' show only the *dpp 2.0-lacZ* channel. **(I and J)** One-day-old control (ctrl) (I) and *bab1>dpp*^{RNAi} germaria (J) with staining for phospho (p)-Mad (gray, Dpp signaling), 1B1 (red, fusomes), LamC (red, TF and cap cell nuclear envelopes) and DAPI (blue, DNA). **(K-M)** One-day-old control (K), *bab1>gfp*^{RNAi} *tkv*^{RNAi} (L) and *bab1>dpp*^{RNAi} *tkv*^{RNAi} germaria (M) with p-Mad (green), 1B1 (red), LamC (red), and *bam-gfp* (blue) labeling. RNAi was expressed from late-L3 to adult (I and J) or throughout developmental stages (K-M) at 25°C. Scale bars, 10 μm. The genotype of the control in A and B is *UAS-tkv*$^{RNAi\ (N)}$/+, in I is *bab1-GAL4/+*, and in K is *bab1-GAL4bam-gfp/+*. ***P < 0.001. Noted that SCC accumulation was milder when the *bab1-GAL4* driver bears a copy of *bam-gfp*, which carries the *bam* coding region fused with GFP under the control of the *bam* promoter (Chen and McKearin, 2003). Perhaps this extra copy of *bam* can lead to some minor compensatory effect.

Supplementary Fig. 4. Knockdown of somatic Tkv slightly increases PGC number, but only *c587>tkv*^{RNAi} gonads exhibited decreased pERK expression. Related to Fig. 4. (A-D) Mid-L3 (ML3) control (A and C), *bab-1>tkv*$^{RNAi\ (N)}$ (B) and *c587>tkv*$^{RNAi\ (N)}$ larval ovaries (D) with 1B1 (green, fusomes) and DAPI (blue, DNA). RNAi was expressed throughout developmental stages at 29°C. **(E)** Average number of PGCs per ovary (gonad) of the indicated genotype. The number of analyzed ovaries is shown above each bar. Error bar indicates standard error; *P < 0.05, ***P < 0.001. The genotype of *bab1-GAL4* control is *bab1-GAL4/+*, and *c587-GAL4* control is *c587-GAL4/+*. **(F and G)** Mid-L3 (ML3) control (ctrl) (F) and *bab1>tkv*$^{RNAi\ (N)}$ gonads (G) with 1B1 (green, fusomes), Tj (red, ICs) and phospho (p)-ERK (gray) labeling. Dashed line indicates terminal filaments. F' and G' only show the pERK channel. Plane A is the same data as shown in the main text, Fig. 4A. Dashed line represent the area used for measuring average (avg.) of pERK signals. **(H)** Average intensity of pERK expression in the region of the control, *c587>tkv*$^{RNAi\ (N)}$ and *bab1>tkv*$^{RNAi\ (N)}$ ML3 gonads containing PGCs and ICs. Number of analyzed gonads are shown above each bar. Error bars, S.D. **, P<0.01. The genotype of the control in A and C is *c587-GAL4/+*.

Supplementary Fig. 5. Forcing Tkv-mediated signaling in the soma activates Egfr signaling in ECs and causes GSC loss. Related to Fig. 4. (A-C) One-day (D)-old control (ctrl), and germaria expressing a constitutively active form of *tkv*, *tkv*^{AC}, driven by *bab1-GAL4* (B) and *c587-GAL4* (C) from mid-L3 to D1. Germaria are labeled with 1B1 (red, fusomes), LamC (red, TF and cap cell nuclear envelopes), and phospho (p)-ERK (gray). A'-C' only show the pERK channel; the asterisks in C and C' indicate a germ cell cyst. **(D-G)** One-day-old control (D), *bab1>tkv*^{CA} (whole stage overexpression) (E), and *c587>tkv*^{CA} germaria (whole stage overexpression) (F and G) with 1B1 (gray) and LamC (gray) labeling. **(H and I)** Number of cap cells (CpCs) (H) and GSCs (I) in one-day-old, *UAS* control, *bab-1>tkv*^{CA}, and *c587>tkv*^{CA} germaria. The number of analyzed germaria is shown above each bar. The genotype of *UAS* control is *UAS-tkv*^{CA}/+. Scale bars, 10 μm. ***P < 0.001.

Supplementary Fig. 6. Hh signaling in the developing ovarian soma, not regulated by Med, promotes PGC differentiation, Tkv controls membrane extension of ECs. Related to Fig. 5. (A and B) Knockdown mad in ovarian somatic cells during development does not affect Hh signaling in adult ECs. One-day (D)-old c587>*gfp*^{RNAi} (A) and *c587>mad*$^{RNAi\ (B)}$ (B) with *ptc-lacZ* (gray, Hh signaling reporter) and DAPI. *RNAi* was expressed from L3 to Day1 at 29 °C. Asterisks mark sheath cells. Scale bar is 10 μm. **(C-K)** Knockdown Hh signaling in the developing ovarian soma results in spectrosom-containing cell (SCC) accumulation. One-day (D)-old control (ctrl) (C and I), *en>hh*^{RNAi} (D), *hh>hh*^{RNAi} (E), *bab1>hh*^{RNAi} (F), *c587>hh*^{RNAi} (G), *bab1& c587>hh*^{RNAi} (H), *bab1>smo*^{RNAi} (J) and *c587>smo*^{RNAi} germaria (K) with staining for 1B1 (green, fusomes), LamC (green, terminal filament and cap cell nuclear envelopes), and Tj (in D-I, magenta, EC nuclei), and DAPI (blue, DNA). *RNAi* was expressed from Late-L3 (LL3) to Day1 at 29 °C. Scale bar is 10 μm. The genotype of C is *UAS-hh*^{RNAi}/+, and of I is *UAS-*

smo^{RNAi}/+. **(L)** Average number of SCC in control, *bab1>hh*^{RNAi}, *c587>hh*^{RNAi} and *bab1& c587>hh*^{RNA} geramaria. Error bars indicate standard error; ****P* < 0.001. **(M-O)** Knockdown *tkv* in the developing ovarian soma disrupts EC membrane extension. One-day-old control (M), *bab1>tkv*$^{RNAi\ (N)}$ (N) and *c587>tkv*$^{RNAi\ (N)}$ (O) with 1B1 (red, fusomes), LamC (red, terminal filament and cap cell nuclear envelopes), and Fax (green, EC). Scale bar, 10 µm. Arrows in M indicate membrane extension of ECs. M'-N' only show the Fax channel. The genotype of M is *UAS-tkv*^{RNAi}/+.

References:

Ashburner, M., (2005). Drosophila: A laboratory Handbook. . Cold Spring Harbor Laboratory Press, Cold Spring Harbor, NY.

Chen, D., McKearin, D.M., (2003). A discrete transcriptional silencer in the bam gene determines asymmetric division of the Drosophila germline stem cell. Development 130, 1159.

Lai, C.M., Lin, K.Y., Kao, S.H., Chen, Y.N., Huang, F., Hsu, H.J., (2017). Hedgehog signaling establishes precursors for germline stem cell niches by regulating cell adhesion. J Cell Biol 216, 1439-1453.

Luo, L., Wang, H., Fan, C., Liu, S., Cai, Y., (2015). Wnt ligands regulate Tkv expression to constrain Dpp activity in the Drosophila ovarian stem cell niche. J Cell Biol 209, 595-608.

Ritsick, D.R., Edens, W.A., Finnerty, V., Lambeth, J.D., (2007). Nox regulation of smooth muscle contraction. Free Radical Biology and Medicine 43, 31-38.

Song, X., Call, G.B., Kirilly, D., Xie, T., (2007). Notch signaling controls germline stem cell niche formation in the Drosophila ovary. Development 134, 1071-1080.

Tseng, C.Y., Kao, S.H., Wan, C.L., Cho, Y., Tung, S.Y., Hsu, H.J., (2014). Notch signaling mediates the age-associated decrease in adhesion of germline stem cells to the niche. PLoS Genet 10, e1004888.

Yan, Y.L., Postlethwait, J.H., (1990). Vitellogenesis in Drosophila: sequestration of a yolk polypeptide/invertase fusion protein into developing oocytes. Dev Biol 140, 281-290.

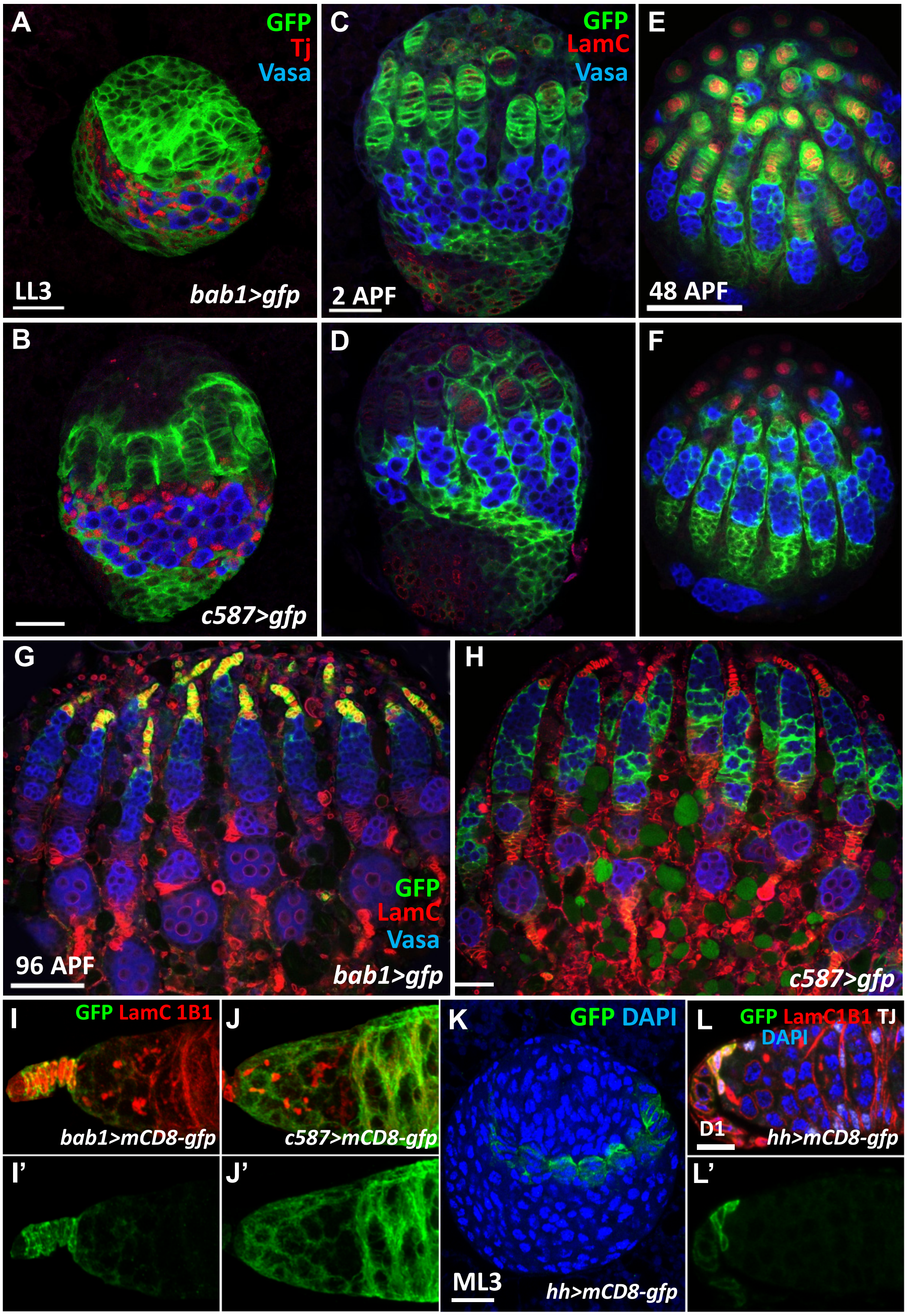

Supplementary Figure. 1

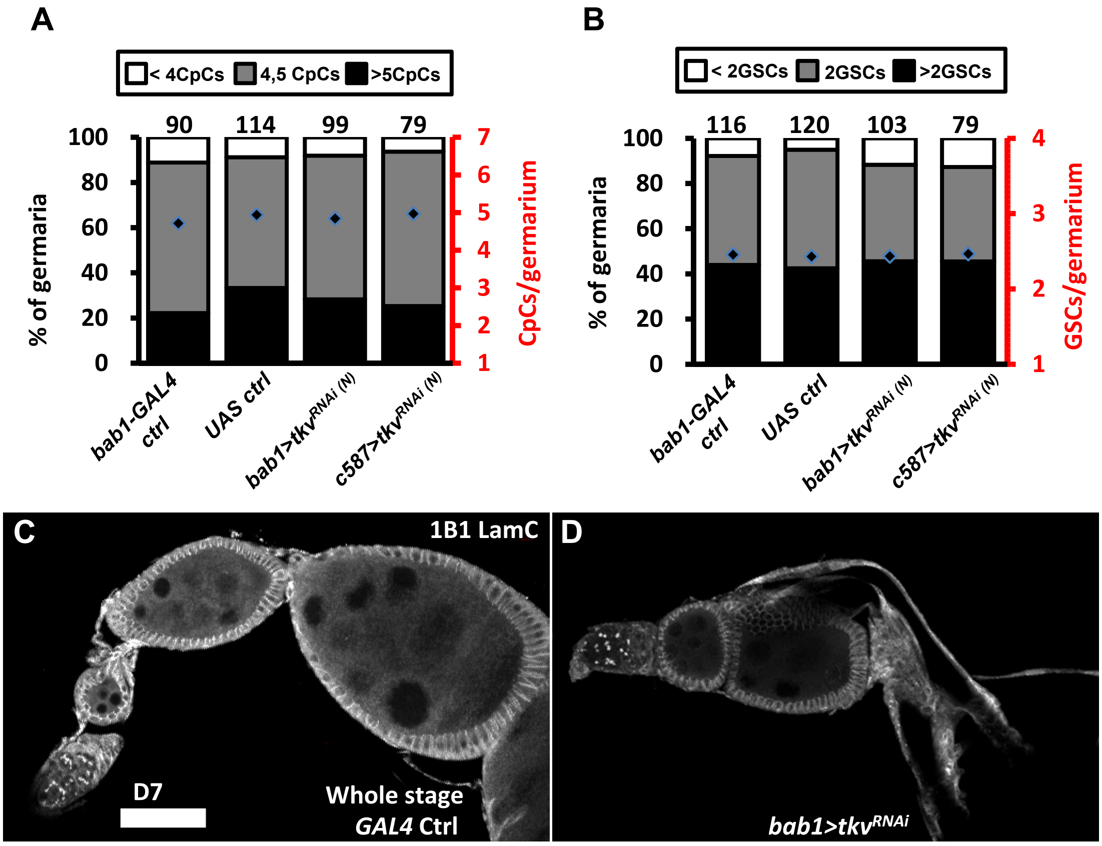

Supplementary Figure. 2

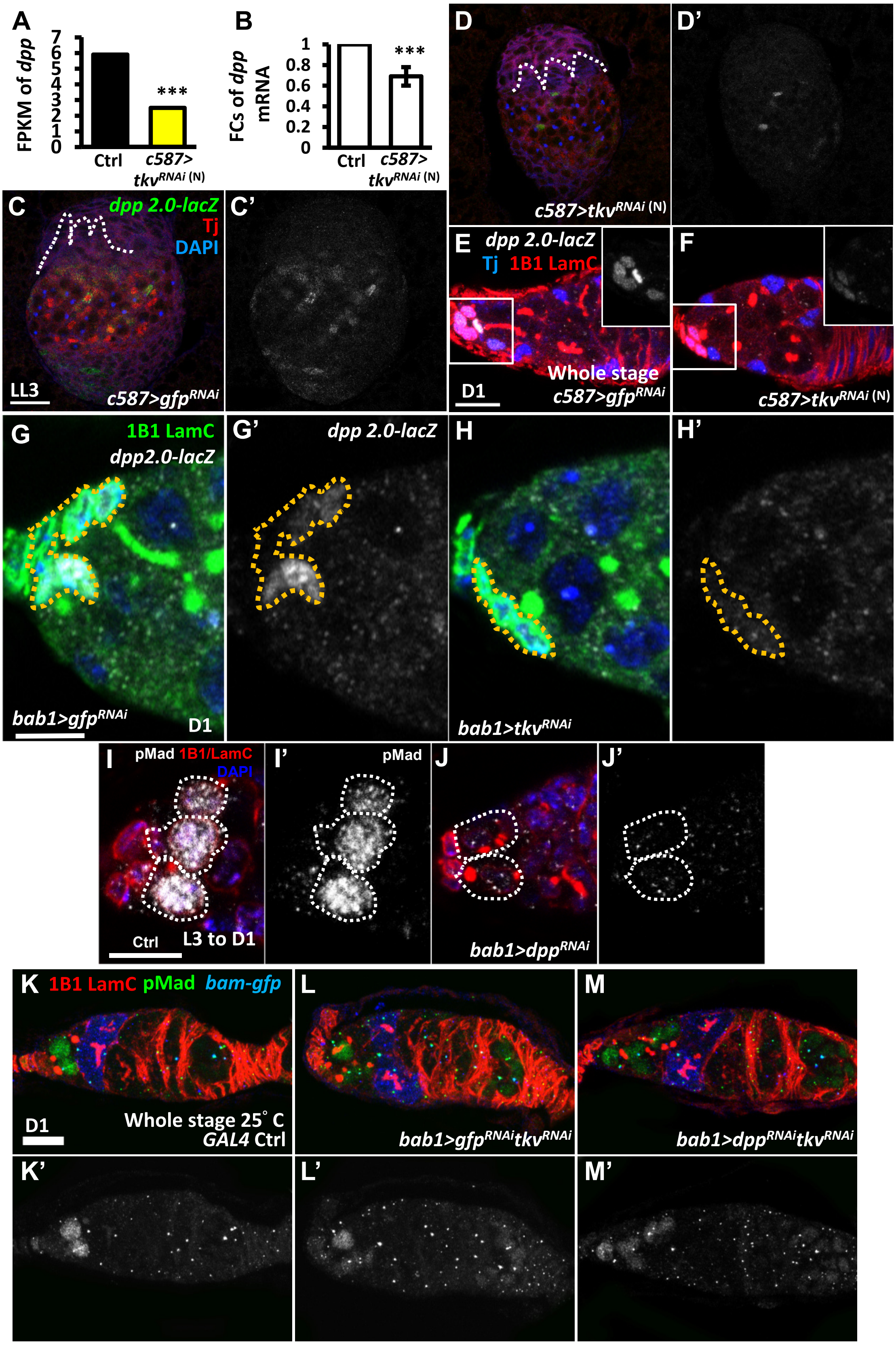

Supplementary Figure. 3

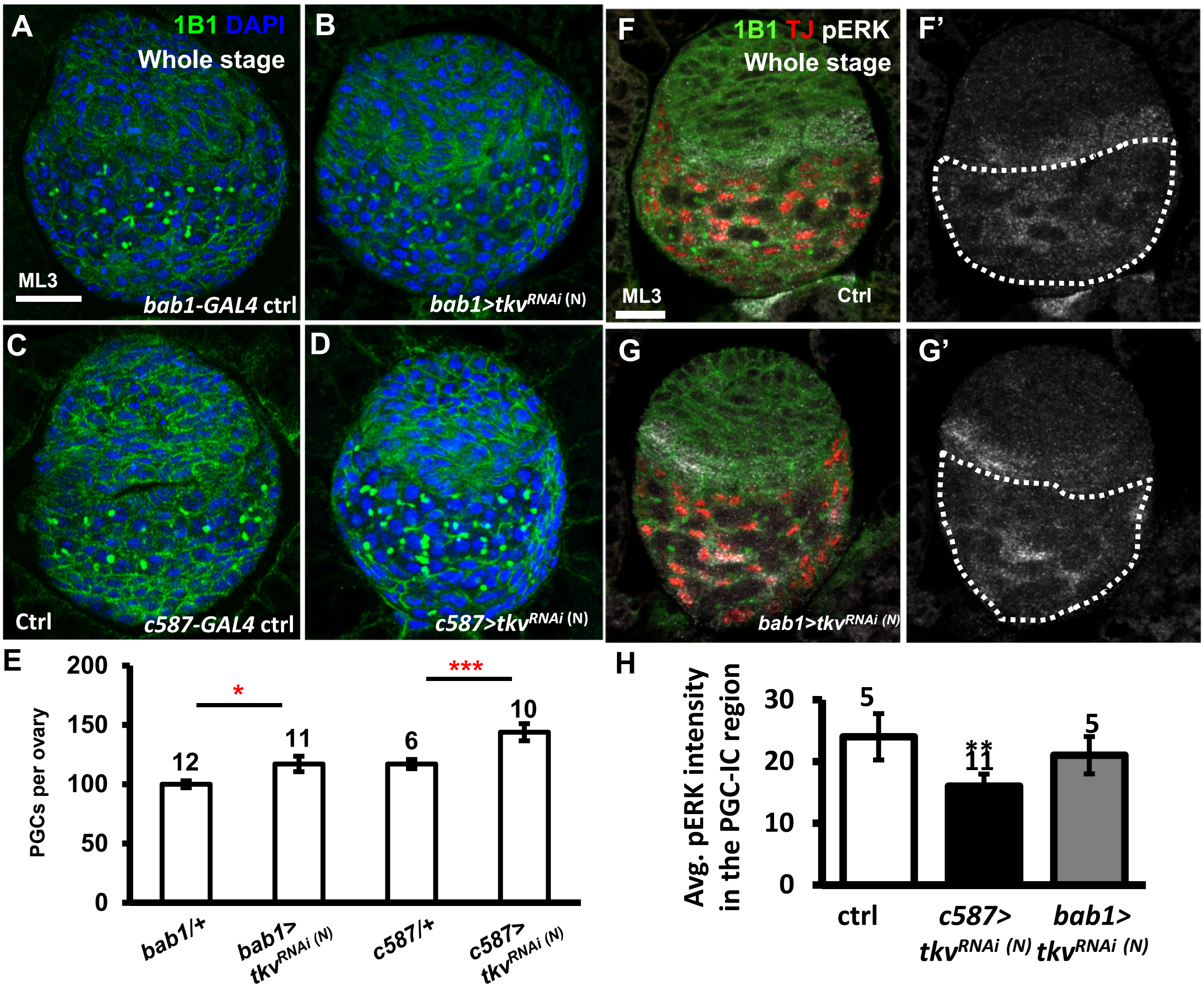

Supplementary Figure. 4

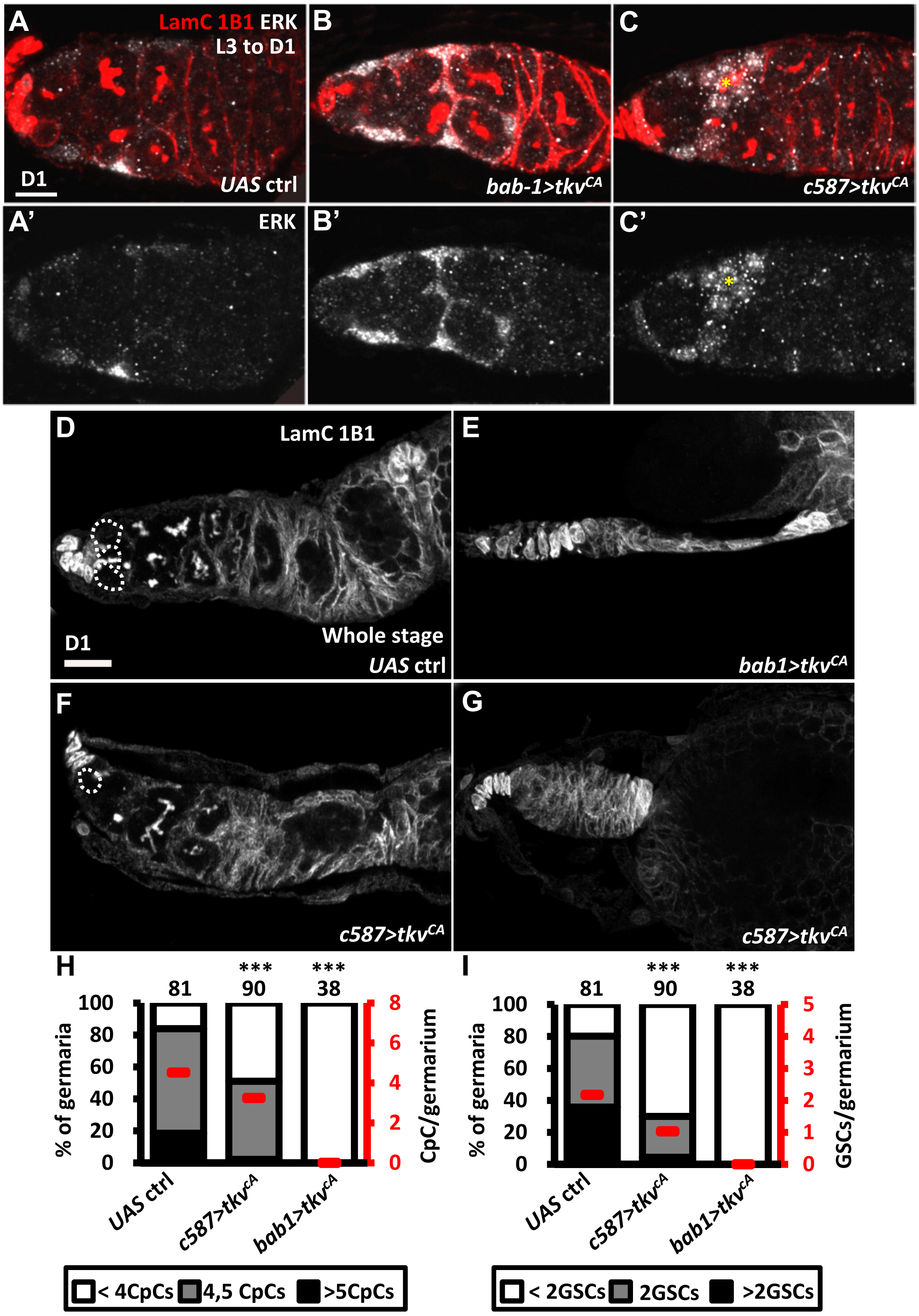

Supplementary Figure. 5

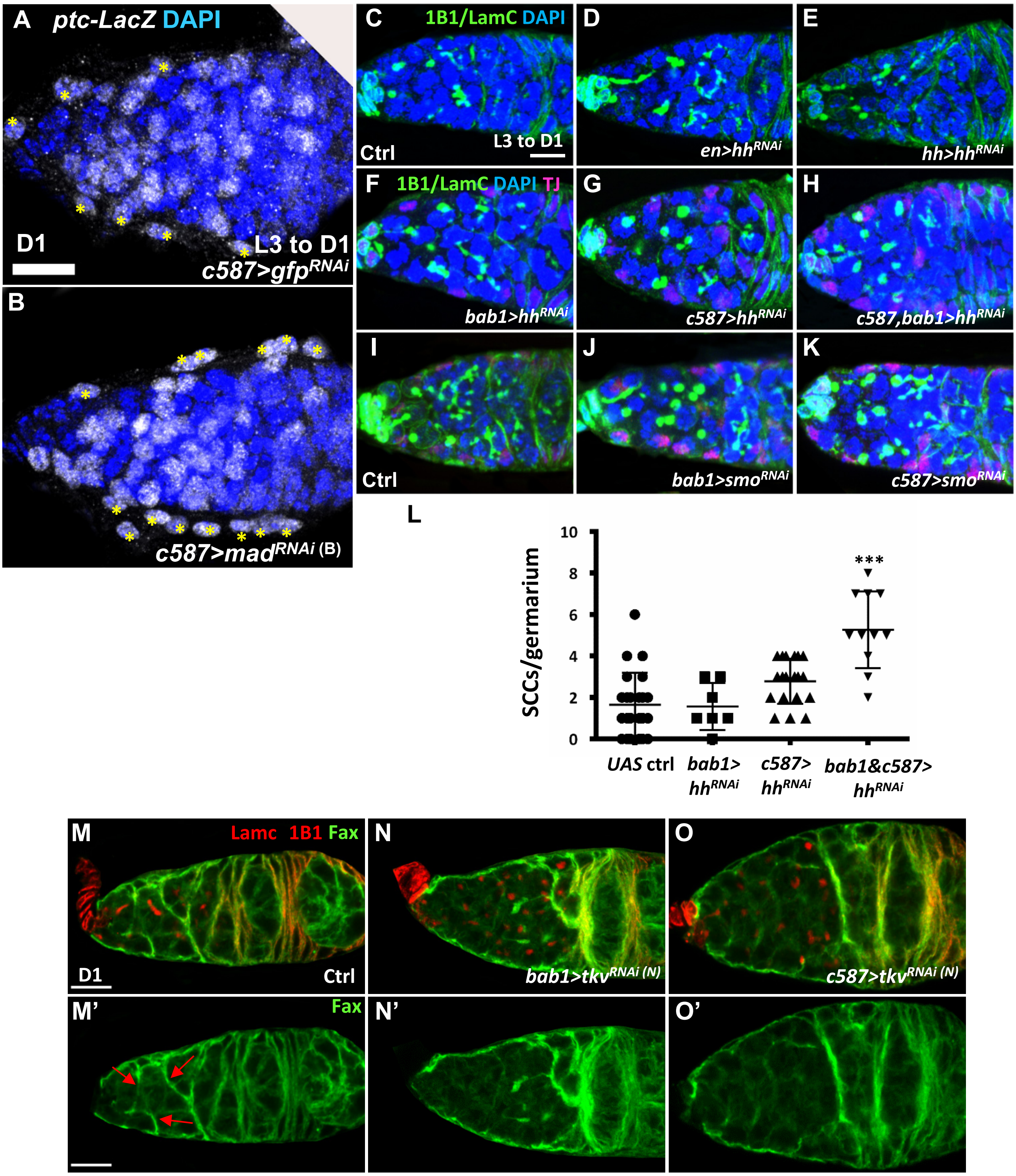

Supplementary Figure. 6
